# A complete human pancreatic cancer genome

**DOI:** 10.64898/2026.05.01.722316

**Published:** 2026-05-10

**Authors:** Justin Wagner, Ayse G. Keskus, Keisuke K. Oshima, T. Rhyker Ranallo-Benavidez, Jennifer McDaniel, Mile Sikic, Dehui Lin, Luis F. Paulin, Adam C. English, Fritz J. Sedlazeck, Elizabeth M. Munding, J. Zachary Sanborn, Andrew Carroll, Pi-Chuan Chang, Daniel E. Cook, Kishwar Shafin, Joep de Ligt, Rayan Hassaine, Daniel Cameron, Severine Catreux, Yeonghun Lee, Lisa Murray, Sean Truong, Christian Brueffer, Aleksey V. Zimin, Erin Cross, Matthew McGowan, Michael Vernich, Andrew S. Liss, Jean-Pierre Kocher, Zachary Stephens, Tanveer Ahmad, Asher Bryant, Nathan Dwarshuis, Hua-Jun He, Zhiyong He, Nathan D. Olson, Francoise Thibaud-Nissen, Dmitry Antipov, Sergey Koren, Adam Phillippy, Rajeeva Lochan Musunuri, Giuseppe Narzisi, Miten Jain, Aaron M. Wenger, Stephen Eacker, Sayed Mohammad Ebrahim Sahraeian, Paul C. Boutros, Yash Patel, Takafumi N. Yamaguchi, Joseph McConnell, Matthew Borchers, Jennifer L. Gerton, Paxton Kostos, Andrea Guarracino, Maryam Jehangir, Hila Benjamin, Mohammed Faizal Eeman Mootor, Yuan Xu, Mobin Asri, Karen H. Miga, Jimin Park, Benedict Paten, Ruibang Luo, Zhenxian Zheng, Jae Young Choi, Linh Nguyen, Pankaj Vats, Dan R. Robinson, Josh N. Vo, Shenghan Gao, Ghulam Murtaza, Christopher E. Mason, Haoyu Cheng, Floris P. Barthel, Chunlin Xiao, Glennis A. Logsdon, Mikhail Kolmogorov, Justin M. Zook

**Affiliations:** 1 National Institute of Standards and Technology, Material Measurement Laboratory, 100 Bureau Dr., Gaithersburg, MD 20899, USA; 2 National Cancer Institute, Cancer Data Science Laboratory, 9000 Rockville Pike, Bethesda, MD 20892, USA; 3 University of Pennsylvania, Department of Genetics, Epigenetics Institute, 3400 Civic Center Blvd., Smilow Center for Translational Research, Philadelphia, PA 19104, USA; 4 Translational Genomics Research Institute, Bioinnovation and Genome Sciences, 445 N 5th St 4th Floor, Phoenix, AZ 85004, USA; 5 A*STAR, Genome Institute of Singapore, 60 Biopolis St, Singapore 138672; 6 Baylor College of Medicine (BCM), Human Genome Sequencing Center (HGSC), One Baylor Plaza, Houston, TX 77030, USA; 7 Baylor College of Medicine (BCM) and Rice University, One Baylor Plaza, Houston, TX 77030, USA; 8 Dovetail Genomics, part of Cantata Bio, Research and Development, 100 Enterprise Way, Suite A101, Scotts Valley, CA 95066, USA; 9 Google Research, Google Inc., 1600 Amphitheatre Pkwy, Mountain View, CA, USA; 10 Hartwig Medical Foundation, Data Services, Science Park 404, 1098 XH, Amsterdam, The Netherlands; 11 Illumina, DRAGEN, 5200 Illumina Way, San Diego, CA 92122, USA; 12 InSilico Consulting AB, Kulgränden 15B, SE-22649 Lund, Sweden; 13 Johns Hopkins University, Department of Biomedical Engineering, Wyman Park Building S237, Baltimore, MD 21205, USA; 14 KromaTiD, 6899 Winchester Cir Suite 100, Boulder, CO 80301, USA; 15 Lund University, Division of Oncology, Department of Clinical Sciences Lund, Medicon Village, Scheeletorget 1, SE-22381 Lund, Sweden; 16 Massachusetts General Hospital and Harvard Medical School, Department of Surgery, Boston, MA 02114, USA; 17 Mayo Clinic, Department of Quantitative Health Sciences, 200 First Street SW, Rochester, MN 55905, USA; 18 National Center for Biotechnology Information, National Library of Medicine, National Institutes of Health, 45 Center Drive, Bethesda, MD 20894, USA; 19 Genome Informatics Section, Center for Genomics and Data Science Research, National Human Genome Research Institute, National Institutes of Health, Bethesda, MD, USA; 20 New York Genome Center (NYGC), New York, NY 10013, USA; 21 Northeastern University, Genome Technology Laboratory, 360 Huntington Ave, Boston, MA 02115, USA; 22 PacBio, 1305 O’Brien Drive, Menlo Park, CA 94025, USA; 23 Phase Genomics Inc., Seattle, WA, USA; 24 Roche Sequencing Solutions, 2881 Scott Blvd, Santa Clara, CA 95050, USA; 25 Sanford Burnham Prebys Medical Discovery Institute, Cancer Center, 10901 North Torrey Pines Road, La Jolla, CA 92037, USA; 26 SA Pathology, Data and Bioinformatics Innovation, Department of Genetics and Molecular Pathology, Frome Rd., Adelaide, South Australia 5000, Australia; 27 Stowers Institute, 1000 E. 50th Street, Kansas City, MO 64110, USA; 28 Ultima Genomics Inc., 4425 Technology Dr, Fremont, CA 94538, USA; 29 University of California Los Angeles (UCLA), Department of Human Genetics, Los Angeles, CA, USA; 30 University of California Santa Cruz (UCSC), Department of Biomolecular Engineering, Santa Cruz, CA, USA; 31 University of California Santa Cruz (UCSC), UC Santa Cruz Genomics Institute, Santa Cruz, CA, USA; 32 University of Hong Kong, School of Computing and Data Science, 301 Chow Yei Ching Bldg., HKU, Pokfulam Rd., Hong Kong; 33 University of Kansas, Department of Ecology and Evolutionary Biology, Haworth Hall, 1200 Sunnyside Ave, Lawrence, KS 66045, USA; 34 NVIDIA Corporation, 2788 San Tomas Expressway, Santa Clara, CA 95051; 35 University of Michigan-Ann Arbor, Department of Pathology, 1500 E Medical Center Dr, Ann Arbor, MI 48109, USA; 36 University of Washington, Department of Genome Sciences, William H. Foege Hall, 3720 15th Ave NE, Seattle, WA 98195, USA; 37 Weill Cornell Medicine, Department of Systems and Computational Biomedicine, 1300 York Ave, New York, NY 10065, USA; 38 Yale School of Medicine, Department of Biomedical Informatics and Data Science, New Haven, CT, USA

## Abstract

Cancer genome sequencing is essential for understanding tumor evolution and advancing precision medicine.^1^ However, reference gaps and germline variants obscure detection of small and large somatic variants and methylation in repetitive regions.^1–3^ It is common for tumor cells to gain or lose chromosome arms due to somatic structural changes that occur inside highly repetitive satellite DNA sequences in the centromeres.^4^ To identify the full spectrum of somatic variants, including complex rearrangements, we construct and curate near-complete, haplotype-resolved assemblies of the most recent common ancestor of an early-passage broadly-consented hypodiploid pancreatic cancer cell line and matched normal tissues. The tumor assembly completely recapitulates all 35 tumor chromosomes observed with karyotyping, with multiple translocation-induced hybrid chromosomes. The hybrid chromosomes contain putative functional dicentric and fused centromeres, nested foldback inversions causing 14 breakpoints with a haplotype switch in a single event, and centromeric satellite tandem duplications up to 136 kbp. Direct comparison of tumor and normal assembly haplotypes uncovers >7,000 variants altering >1 Mbp of sequence in repetitive regions that have been hidden by reference gaps and germline variants. 44 % of somatic small variants change representation because they alter germline variants on GRCh38, impacting mutational signatures and kataegis/omikli clusters. Most somatic LINE insertions originate from two hypomethylated non-reference germline LINE insertions, highlighting their impact on insertion mutation burden. These assemblies demonstrate that centromeric, acrocentric, and telomeric regions conventionally excluded from analysis harbor extensive somatic and epigenetic changes. Resolving complete tumor genomes enables a deeper understanding of cancer structural plasticity and the endpoints of breakage-fusion-bridge cycles. These assembled, curated paired normal-tumor benchmarks will serve as a critical foundation for developing future algorithms to characterize the most intractable regions of cancer genomes.

## Introduction

Tumor genomes often have complex somatic changes relative to the germline genome from which they evolve.^1^ It is common for tumor cells to gain or lose chromosome arms due to somatic structural changes that occur inside highly repetitive satellite DNA sequences in the centromeres.^4^ These repetitive regions in normal genomes have only recently been tractable to resolve with the advent of complete “Telomere-to-Telomere” (T2T) assemblies of effectively haploid^5^ and diploid genomes.^6^ For somatic variants, mapping tumor and normal reads to a donor-specific assembly (DSA) of the normal genome has been shown to improve somatic variant calling and epigenetic analysis.^2,3,7,8^ DSAs expand prior simulated^9,10^ and tumor/normal benchmarks for legacy cell lines^11–13^ that largely excluded repetitive regions, lacked phasing, and focused on one variant type. While DSAs of the normal genomes are now possible, tumor genome assemblies are fragmented with current algorithms due to subclonal variation between cells and large, nearly-identical duplication events.

Here, we use extensive genomic data from a hypodiploid tumor cell line and normal pancreatic and duodenal tissues from the same recently broadly-consented germline XX individual with pancreatic ductal adenocarcinoma (PDAC).^14^ In this genome from HG008, we previously reported clonal somatic variants in *KRAS*, *TP53*, *SMAD4*, and *CDKN2A*, four of the most commonly mutated genes in PDAC.^14^ Here, we generate phased benchmarks for all variant types from near-complete haplotype-resolved assemblies of the normal genome and the genome of the most recent common ancestor of the tumor cell line. With extensive curation and polishing, these assemblies precisely resolved phased small variants, structural variants, and copy number variants, including complex rearrangements involving multiple chromosomes, translocations with breakpoints in centromeres, and repeat expansions and contractions in regions missing from the reference genome. The assemblies resolve distinct centromere structures for rearranged chromosomes. Finally, we developed and evaluated somatic variant benchmarks for all variant types that will enable technology and bioinformatics development for challenging complex variants.

## Results

### Direct comparison of tumor to normal assemblies recapitulates all large truncal somatic structural and copy number variation

**Fig. 1:**
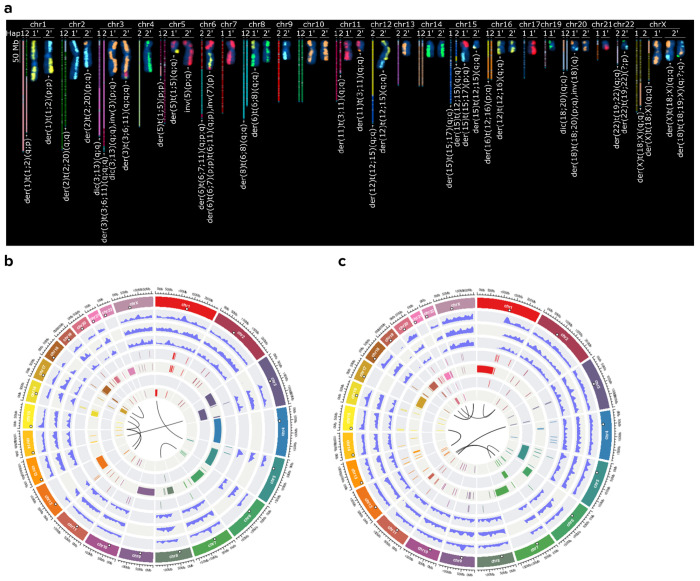
Complete haplotype-resolved tumor and near-complete normal assemblies. (a) KaryoScope visualization of the complete tumor assembly matches all chromosomes consistently seen in dGH SCREEN karyotyping (representative cell shown), which paints one strand of each chromosome one of five distinct colors. For each chromosome, KaryoScope is on the left and dGH is on the right, including ISCN notations. (b-c) Overview of haplotype-specific somatic SNVs (density plot with default 10 Mbp window) and SVs from HG008T-hap1 (b) and HG008T-hap2 (c). HG008N centromere positions are indicated by white circles on each chromosome. The track order (from outer to inner) is density of somatic SNVs, insertions <50 bp, deletions <50 bp, insertions ≥50 bp, deletions ≥50 bp, inversions, duplications, and translocations, respectively.

To assess tractability and structural accuracy of HG008 tumor genome assembly, we used the directional genomic hybridization (dGH) SCREEN assay^15^ (Fig. 1a). Almost all 80 cells from passages 21 and 53 shared common structural changes matching assembly-based copy number profiles and structural variants, apart from subclonal variants occurring in 1 to 4 cells (Supplementary Table S1). While subclonal variants differ between passages, the variants characterized in this work are relatively stable and in most cells across passages. Thus, the mutations characterized here are all truncal (*i.e.* occur in the trunk of the evolutionary tree, leading to the most recent common ancestor, MRCA). Most are also clonal (i.e. present in all cells), but a subset are subclonal due to loss in descendent passages, mostly due to large deletions.^10^

We generated haplotype-resolved, near-complete assemblies of the MRCA of the hypodiploid HG008 PDAC tumor cell line and its matched normal genome by adapting T2T assembly workflows.^16^ By curating the assembly graph, we achieved T2T scaffolds for all 35 tumor chromosomes with 9 gaps (8 due to rDNA arrays) and 44 T2T scaffolds for the normal genome. Systematic comparison of dGH to assembly-based karyotyping using KaryoScope^17^ showed complete concordance in translocation partner identification across all 16 truncal inter-chromosomal rearrangements, including unidentified ‘marker’ chromosomes from standard G-banded karyotyping (Table S2). Polishing using Element^18,19^ and Onso^20^ short reads fixed > 100,000 indel errors, particularly in homopolymers.^6^ Validation through kmers, Hi-C mapping, haplotype-specific markers, and gene-completeness analysis confirmed high structural and phasing accuracy (Supplementary Figures 1–4). While some errors remained, particularly around gaps in the normal assembly and homopolymers inside somatic duplications, these assemblies captured all truncal variation detected by karyotyping and reference-based approaches, providing a comprehensive, high-resolution foundation for identifying clonal somatic variants and tumor chromosome structure (Fig. 1).

The assemblies completely resolve truncal somatic CNVs and the translocations causing them, including complex, centromeric, and acrocentric breakpoints typically left unresolved (Supplementary Fig 5).^4^ The prevalence of large deletions in HG008 (Supplementary Fig 6) aligns closely with chromosomes and chromosome arms commonly lost in PDAC and in the chromosome-scale LOH signature from TCGA.^21^ Translocations caused only three large duplications or CNLOH events (Supplementary Fig 7). The assembly phases all events, including loss of each chr6 arm on opposite haplotypes, deleting a copy of the MHC region, which may affect immunogenicity (Supplementary Table S3). Phasing also elucidates a balanced chr18_hap2/chrX_hap2 translocation on the copy of chrX that is mostly transcriptomically silenced (Supplementary Fig 8). Interchromosomal translocations in HG008 are frequently complex in the sense that they cannot be described as a single pair of breakpoints, commonly due to non-reciprocal inversions. In addition to three reciprocal inversions, we resolve nine non-reciprocal foldback inversions related to complex interchromosomal translocations, five inversions that do not cause duplications near translocations, and one inversion related to a tandem duplication (Supplementary Fig 9).

**Fig. 2:**
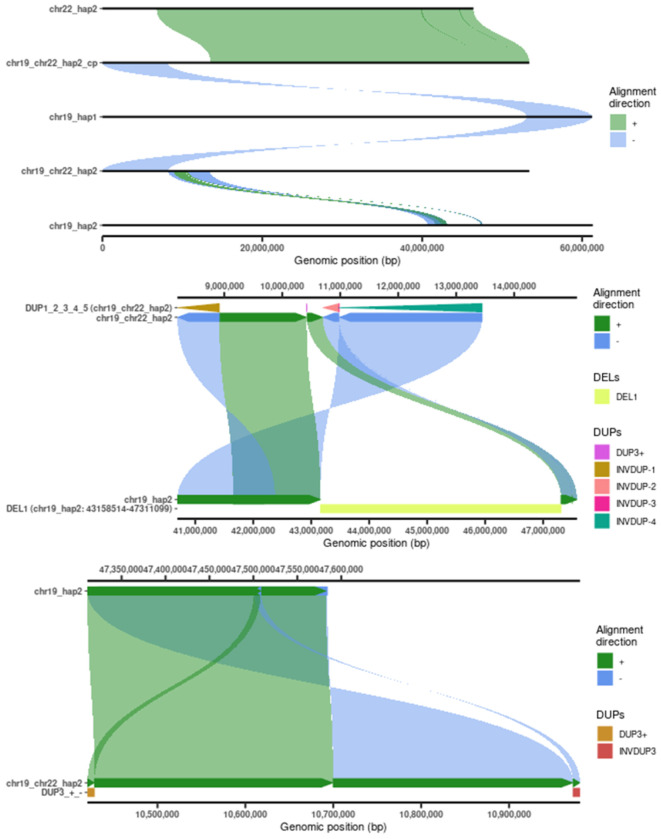
The chr19_chr22_hap2 hybrid tumor chromosome assembly reveals complex foldback inversions at multiple resolutions, plus a haplotype switch. der(22)t(19;22)(q;p) has a breakpoint in the acrocentric short arm of chr22, causing loss of 6.8 Mbp of the chr22 acrocentric short arm including the rDNA array and distal junction, as well as a 97 kbp foldback inverted duplication of chr22 adjacent to the centromere. It is connected to a series of nested foldback inversions of chr19_hap2, before being connected to the last 8 Mbp of chr19_hap1. The assembly resolves high-level structure (top) as well as complex inverted duplications of 8 kbp to 2.5 Mbp fragments at basepair resolution (middle and bottom).

The tumor assembly resolved several translocations involving multiple chromosomes. For example, chromoplexy resulted in a complex series of translocations involving chromosomes 3, 6, 7, and 11 to form 3 new hybrid chromosomes (Supplementary Fig 10). The most complex somatic structural variation and copy number variation in HG008 occurred on the parts of two haplotypes of chr19 attached to most of chr22 (Fig. 2). The translocation between chr22 and chr19 occurred with a foldback inversion in the pericentromere of chr22, between the rDNA and centromere on the normal assembly. On chr19, the assembly resolves a nested series of foldback inversions with 11 breakpoints causing copy numbers from 1 to 5, consistent with multiple breakage-fusion-bridge cycles, and the last 8 Mbp of chr19 comes from the opposite haplotype (Supplementary Fig 2c). The assembly resolves another acrocentric short arm translocation between chr15 and chr17, with 210 bp of chr11 in between (Supplementary Fig 11). All of these large translocations were confirmed by Hi-C contact maps and curation, forming a comprehensive benchmark (Supplementary Fig 12).

### Somatic variants of different types and sizes are enriched in different repeat types

**Fig. 3:**
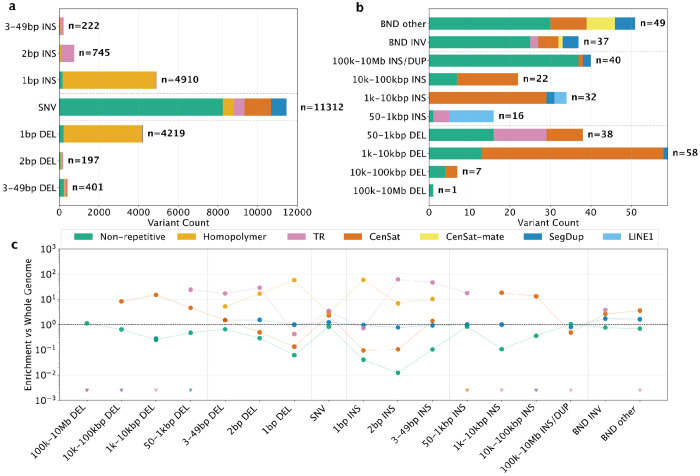
Somatic variant types are enriched in different repetitive regions, based on confirmed truncal variants derived from comparing the tumor and normal assemblies. (a) Small variant counts show substantial numbers of SNVs in repetitive regions and most indels in homopolymers and TRs. (b) SV counts show many SVs in repetitive regions, particularly for 50 bp to 100 kbp insertions. (c) Variants across types and sizes are generally enriched in repetitive regions relative to the whole genome. Triangles indicate no variants. Dashed lines separate variant types.

Directly comparing the tumor and normal assembly haplotypes gives a more precise and comprehensive view into how different types and sizes of somatic variants are associated with repetitive genomic regions (Fig. 3). In particular, assemblies reduce biases caused by germline variation and missing reference sequences in repetitive regions. All variant types except SNVs, tandem duplications > 100 kbp, and translocations predominantly occur in repetitive regions, and even these are enriched in many types of repeats (Fig. 3 and Supplementary Fig 13). Somatic insertions are particularly enriched in repetitive regions, with >96% of insertions from 1 bp to 100 kbp occurring in homopolymers, tandem repeats, or centromeric satellites, except for LINE1 insertions (Fig. 3c). Similar to germline variants, while most somatic variants are small (22,006 variants <50 bp, modifying 28,132 bp), the fraction of the genome modified by large somatic variants is higher (214 variants ≥50bp, modifying 28,093,114 bp, not including large CNVs caused by 86 translocation and inversion breakpoints) (Supplementary Fig 13). Insertion:deletion ratios are a complex function of repeat unit size, repeat length, and variant type (Fig 3 and Supplementary Figs 13–14).

While most variant types are associated with particular repeat types, large somatic deletions and tandem duplications that could be represented on GRCh38 tend to have simple breakpoints in non-repetitive regions. Important genes affected include a 20 kb deletion of the remaining copy of *CDKN2A*, a 2 Mbp tandem duplication of the *KRAS* G12V mutated gene that was associated with advanced disease,^22^ and Internal tandem duplications in *NFE2L2*, *UBR3*, and *BBS9* (Supplementary Fig 15). For other variant types, repeat structures dictate the types of mutations that occur and the challenges in detecting them, so we next investigated the specific dynamics within each major repeat class.

### Four types of centromeres in rearranged tumor chromosomes

We examine centromere structure in the rearranged HG008 tumor chromosomes because centromeres have long been implicated in aneuploidy^23^ and chromosome arm loss and were recently shown to have higher somatic mutation rates.^2,3^ Of 16 hybrid tumor chromosomes composed of multiple normal chromosomes, 9 clearly retained only one centromere with breakpoints outside the centromeres (2 reciprocal translocation pairs in Supplementary Fig 16 and 5 non-reciprocal translocations in Supplementary Fig 10–11, 17). However, 7 had a breakpoint in or near one or both centromeres (Supplementary Table S4–7), causing chromosome arm losses in HG008-T that are common in all cancers and/or the chromosome-scale LOH signature (*e.g.*, chromosomes 1p, 3p, 6p, 6q, 8p, 18q) in TCGA samples^21^ and in PDAC samples (6p, 6q, 8p, 18q).^23^

**Fig. 4.**
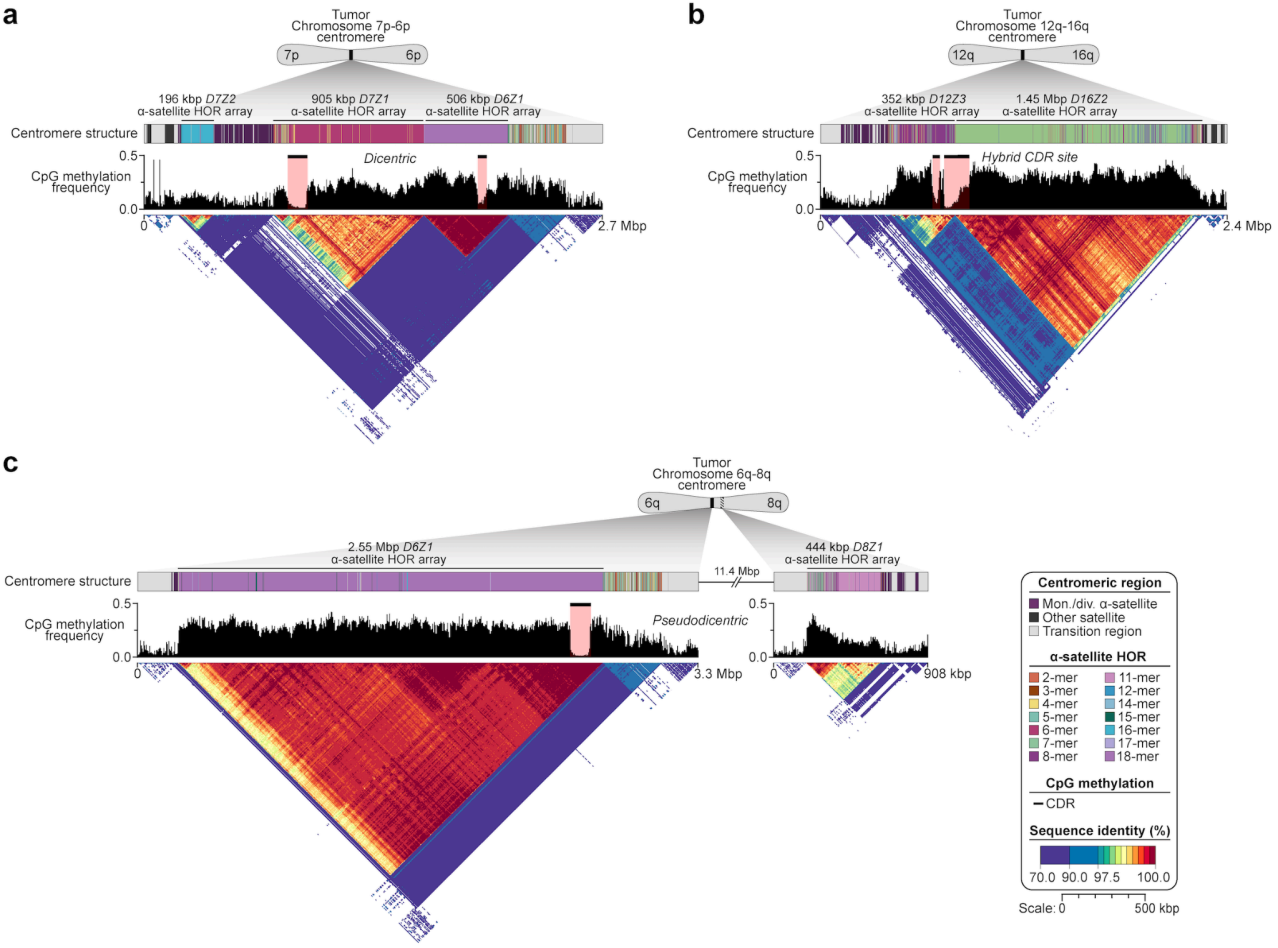
Plasticity of human centromeres during tumorigenesis. a) A dicentric chromosome is formed upon fusion of 6p and 7p, resulting in two CDRs that are associated with the site of kinetochore assembly within the *D7Z1* and *D6Z1* α-satellite HOR arrays (*i.e.*, CDRs are low methylation regions highlighted by black bars and pink rectangles in the CpG methylation track). b) A monocentric chromosome is formed when chromosomes 12q and 16q fuse, creating a hybrid CDR that resides on two different types of α-satellite HOR arrays (*D12Z3* and *D16Z2*). c) A pseudodicentric chromosome is formed upon translocation of 6q and 8q, resulting in an active CDR site on the *D6Z1* α-satellite HOR array and an inactivated CDR site on the *D8Z1* α-satellite HOR array. The inactivation of the *D8Z1* α-satellite HOR is caused by the translocation that removes ~2 Mbp of the *D8Z1* α-satellite HOR array.

To determine if these 7 chromosomes had formed putative functional dicentrics, we identified all centromere dip regions (CDRs), which are associated with kinetochore function.^24^ We found that one chromosome formed a putative functional dicentric, with the chr6 and chr7 CDRs located ~1 Mbp apart in the α-satellite HOR array (Fig. 4a, Supplementary Fig. 10 and 18). We also observed another chromosome that had fused within both chr12 and chr16 CDRs, resulting in a single CDR site composed of two different α-satellite HOR arrays (Fig. 4b, Supplementary Fig. 17 and 19). We identified three pseudodicentric chromosomes with one functional centromere and one inactivated centromere. The chr8 CDR is deleted by the interchromosomal translocation (Fig. 4c). Large inversions related to the translocation delete the CDR in chr3, but do not delete the CDR in chr18 so its CDR is inactivated by methylation (Supplementary Figs. 9, 20–22). Two monocentric chromosomes had breakpoints within the chr1 α-satellite HOR or immediately adjacent to the chr22 α-satellite HOR but preserved the CDRs (Supplementary Figs. 23–25). Together, we observe four different types of centromeres in the tumor chromosomes with translocations: one dicentric, one hybrid centromere, three pseudodicentrics with different causes of centromere inactivation, and eleven monocentrics, underscoring the extreme plasticity of human centromeres in order to preserve function by modulating either genetic or epigenetic landscapes.

### Most multi kbp SVs are in centromere satellite regions only visible with assemblies

The assemblies enabled precise resolution of many translocations, insertions, and deletions in centromere satellites, where most SVs occur in HG008. 300 SVs were confirmed as likely true truncal somatic SVs by most tumor but not normal PacBio HiFi or ONT reads aligned to the combined haplotypes. Chromosome 1 is particularly rich in challenging SVs with 25 of 28 truncal SVs in centromeres, as well as a deletion of one monomer in a 5S rDNA (Supplementary Fig 26).

Analysis of all 117 somatic insertions and deletions in centromeric and satellite DNA regions of HG008-T revealed that somatic insertions were significantly larger than deletions across all satellite types (Supplementary Fig 27). The four largest confirmed somatic insertions of 61 kbp to 136 kbp are larger than germline or somatic de novo centromeric insertions seen in recent studies (Supplementary Fig 28). The large insertions are tandem duplications, so are challenging to detect without assembling both tumor and normal reads so may have been invisible to mapping-based approaches used in previous somatic studies. As expected, smaller somatic SVs < 5 kb in α-satellite HORs showed strong periodicity at 171 bp intervals (Supplementary Fig 27).

**Fig. 5:**
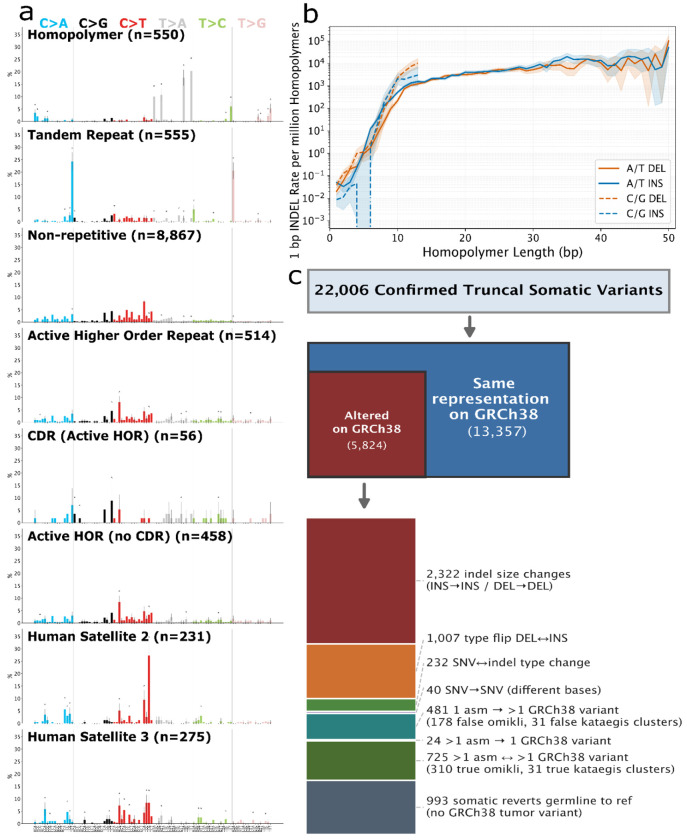
Challenging Somatic Small Variants. (a) SBS96 substitution signatures vary across some repetitive regions using the DSA. * indicates significant difference from the whole genome (uncorrected p<0.0001, with correct FDR<0.01 per region). (b) Truncal somatic 1 bp INDEL rate vs. A/T and C/G homopolymer length with 95% confidence interval shaded. (c) Many somatic small variants are lost or have altered representation on GRCh38 vs the true somatic change on the DSA due to germline variants.

Small somatic variants that could not be lifted to GRCh38 were enriched for SNVs vs. small indels, likely because centromeric satellites contain fewer homopolymers. Similar to what was found recently for UV-damage somatic SNVs in a melanoma cell line,^2^ somatic SNVs in HG008 (not from UV damage) were also enriched in centromeric satellites relative to non-repetitive regions, particularly in CDRs of α-satellite HORs, dHORs, and HSat2 (Supplementary Fig 29). Mutational profiles were mostly similar between HORs and non-repetitive regions, but those in HSat2 were significantly different, primarily due to differences in sequence composition (Fig. 5a and Supplementary Fig 30).

### Somatic variants in homopolymers and tandem repeats are obscured by germline variants

Somatic variant calling in homopolymers and tandem repeats faces multiple challenges caused by high rates of germline variants, somatic mutations, systematic sequencing errors, and mapping errors. For example, all STRs and VNTRs with somatic SVs also have large germline insertions and/or deletions in HG008, so that the true somatic variant size is not apparent on GRCh38, and four also have evidence for mosaicism in the normal tissues. In addition to TR expansions and contractions, breakpoints on one end of a complex 35 Mbp somatic inversion on chr7 fall inside a 611 bp VNTR that also has germline indels. Some somatic small variants are hidden inside germline insertions in TRs, including a stop-gain somatic SNV near the end of a 63 bp germline insertion in a VNTR in *PRB4*. Multiple VNTRs like the one in *PRB4* are also located inside segmental duplications, causing additional mapping challenges (more examples in Supplementary Figs 31–35).

Accurate truncal small variants in homopolymers and TRs were identified by requiring support in accurate short reads and/or long reads from the tumor but not normal when mapped to the DSA. Deletions are enriched in very short and very long G/C homopolymers, whereas insertions are enriched in short and 35-36 bp A/T homopolymers and dinucleotide TRs (Fig. 5b, Supplementary Fig 36). In addition, most large indels inside homopolymers are deletions in long homopolymers (Supplementary Fig 34).

Variants are missed by reference-based methods because they cannot be lifted to GRCh38 (such as in centromeres and telomeres), fall in regions with germline CNVs, revert a germline variant to reference, change type or size due to germline variation, or fall in difficult regions (Supplementary Fig 35, 37–41). 5,824 of 19,181 variants in GRCh38 benchmark regions changed type or size due to germline variants (Fig. 5c). These changes can affect mutational signatures in repeats (Fig 5a, Supplementary Fig 30, Supplementary Fig 42–43). They also cause 481 false omikli and kataegis due to complex germline-somatic interactions and 395 false omikli from variants on opposite haplotypes. Analyzing true clusters of variants on each haplotype using the DSA, 68 kataegis events containing 480 variants and 1,509 omikli events containing 3,169 variants, with DSA-specific clusters enriched in telomeres and centromeric satellites. Besides kataegis and omikli, the inactive chrX_hap2 has more SNVs and indels across most of the chromosome, consistent with previous studies showing hypermutation of inactive X is common in cancer (Supplementary Fig 44).^25^

### Many somatic LINE insertions originate from two germline LINE insertions

The 15 insertion SVs outside tandem repeats and centromeric satellites were all LINE retrotranspositions (Supplementary Table S8). Strikingly, 6 of 9 with an identifiable source were derived from two germline L1 insertions not in GRCh38: five from the chr14 source that has a population frequency of 14 % and one from chr15 that has 5.3 % population frequency in CoLoRSdb long reads.^26^ The promoters of the germline L1s on chr14 and chr15 were hypomethylated in the tumor relative to the normal. This suggests that germline ‘non-reference’ L1 insertions were particularly important for this individual’s somatic L1 insertion rate. Other identifiable source L1’s on chr11 and chr22, which also were hypomethylated in tumor but not normal reads, resulting in one and two somatic insertions respectively (Supplementary Fig 45). Six additional L1 somatic insertions were detected where the source L1 could not be determined. The somatic L1 insertions have modal poly-A tail lengths ranging from 29 bp to 113 bp, and many have highly variable lengths between cells based on HiFi reads (which might be caused by rapid shortening^27^), with the largest range from 26 bp to 152 bp for SV_88 on chr11, with a bimodal distribution (Supplementary Fig 46). Some poly-A’s are substantially longer than a previously reported germline L1S distribution of 10 bp to 65 bp,^28^ similar to those seen recent disease-causing insertions.^29^ Of note, transduction sequences of somatic L1 insertions with interspersed poly-A tracts reveal not only the source L1 element, but also occasionally past lineages. For example, for somatic SV_34, the source L1 on chr15 was itself likely derived from either chr6 or chrX. One somatic L1 insertion lies within the centromeric α-satellite HOR (400 kbp upstream of the CDR) on chr1_hap2, so it is only detected using the DSA. While germline L1 insertions have recently been identified across most chromosomes’ α-satellite HORs, they are relatively rare on chr1.^30^

### Telomeres are shortened, including TVRs, and contain translocations

Somatic changes are particularly prominent in the telomeres. While the allele telomere lengths in the pancreatic normal tissue are 6991 ± 2134 bp, the telomeres of the tumor cell line are almost completely eroded, with lengths of 549 ± 216 bp (Supplementary Fig 47–48). Interestingly, most of the telomeres in the tumor cell line eroded down to the most distal telomere variant repeat (TVR) but not significantly further, suggesting that telomeres effectively begin at the canonical tract and that the preceding regions comprised of canonical TTAGGG repeats interspersed with TVRs do not have telomeric function. However, TVRs were observed to be frequently altered, with an enrichment of somatic deletions up to 182 bp in size affecting TVRs, possibly attributable to somatic restructuring during cycles of telomere shortening and elongation.^31^ Telomeric translocations that may be related to shortened telomeres occur at the chr13q telomere with chr3, and in the chr15p acrocentric short arm telomere with chr17.

### Ribosomal DNA regions are impacted in a pancreatic tumor context

**Fig. 6.**
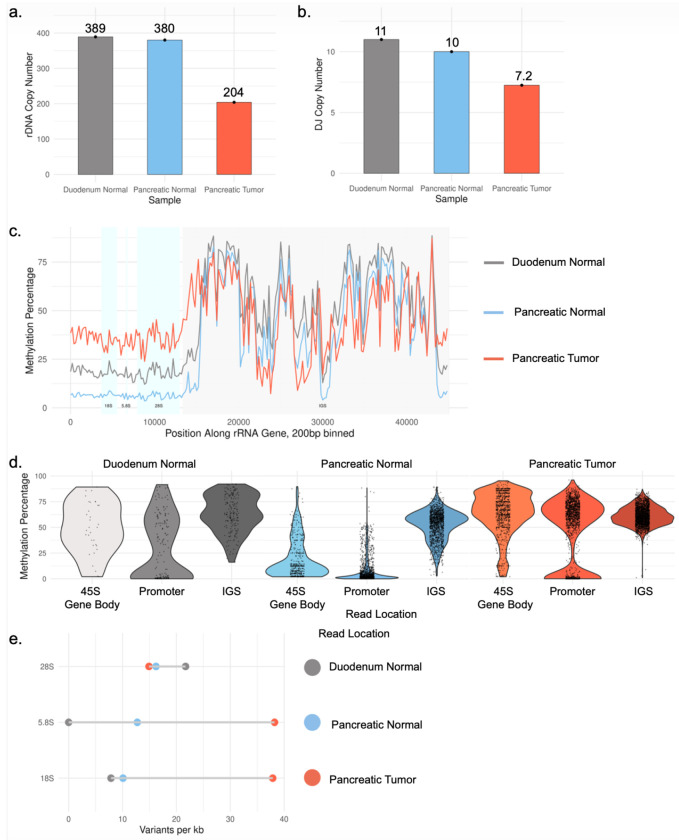
Ribosomal DNA Regions are Impacted in a Pancreatic Tumor Context. a) rDNA and b) rDNA-adjacent distal junction (DJ) copy number estimates in the three samples, with a decrease in the tumor relative to both normal samples, which share similar copy numbers. c) Average methylation for each sample was derived from ONT reads mapped to an rDNA reference gene. Regions within the rRNA gene body that comprise the mature 18S, 5.8S, and 28S rRNAs are shaded in blue, while the IGS region is shaded in grey. Bin size, 200 bp. d) Violin plots depict the distributions of DNA methylation levels across the promoter, gene body, and IGS, derived from ONT reads. Points represent per-read average methylation values within each region. e) Variant density (variants per kb) across rRNA gene regions show normal tissues predominantly exhibit variation in 28S rRNA region, while the tumor sample exhibits increased variant density in the typically conserved 5.8S and 18S genes, with relatively stable variation in the 28S.

The five short arms of the human acrocentric chromosomes harbor the ribosomal DNA (rDNA) arrays, large tandem repeats encoding the 47S rRNA precursor that drive nucleolus formation and ribosome biogenesis. The status of the rDNA is particularly relevant in cancer, a proliferative state that depends heavily on ribosomes, where rRNA gene arrays have been proposed to be recombinational hotspots.^32^ We determined that the tumor sample lost nearly half of the rRNA gene copies relative to the two normal tissues (Fig 6A). Previous work has shown copies are often lost in tumor tissue relative to normal tissue controls.^33,34^ However, it has not been clear whether the loss of genomic material was confined to the rDNA arrays, or caused by larger deletions. In HG008, we confirmed the tumor assemblies lost 3 of the 10 to 11 expected normal^35^ rDNA array regions due to loss of entire chromosomes or acrocentric short arms (Fig 6B).

Next, we examined DNA methylation in ONT reads from the HG008 normal pancreatic and duodenal tissue samples and tumor cell line. In previous analysis of cultured cell lines, the intergenic spacer region (IGS) was consistently methylated, but the 45S gene body, which encodes the mature 18S, 5.8S, and 28S rRNAs, was methylated when silenced, or unmethylated when transcribed.^36^ While the IGS methylation was consistent, methylation of the gene body was different in each of the three samples (Fig. 6C).^36,37^ Counterintuitively, the tumor tissue had the highest level of methylation over the gene body, despite the loss of rDNA copies and presumed heavy reliance on the remaining genes for ribosome biogenesis. Analysis of individual reads, quantified as per-read average methylation across rDNA regions, reveals the tumor sample has a population of reads with very low methylation, suggesting possible transcription (Fig. 6D). While most acrocentric rDNA arrays are not resolved in the assemblies, they resolve a truncal somatic 2,232 bp deletion of one monomer unit in a methylated region of the 700 kbp 5S rDNA array on chr1_hap1. Most of the array is nearly fully methylated, but the 40 kbp on the right side of the array is unmethylated, similarly in both tumor and normal, unlike the 45S gene that has differences in methylation.

Finally, we found SNVs are largely confined to the 28S region of the gene, while the 18S and 5.8S regions remain highly conserved in both normal tissues (Fig 6E).^38^ In contrast, the pancreatic tumor sample has increased low frequency SNV density in the normally constrained 18S and 5.8S regions. Together, this suggests that sequence variation can accumulate within rDNA units during tumorigenesis, and the evolutionary pressures that normally constrain variation break down.

In the tumor sample, loss of rRNA gene arrays, increased gene body methylation, and the accumulation of variants within canonically conserved rRNA coding regions, point to widespread instability of this essential genomic region. Although we have not measured expression of these variants, they may cause a shift in the pool of template rRNAs available for ribosome biogenesis.^39,40^ These observations illustrate how near-complete assemblies enable interrogation of rDNA sequence, copy number, and epigenetic state and reveal that the challenging acrocentric p-arm regions can harbor important structural and epigenetic changes in cancer.

### Creation of comprehensive, curated somatic benchmarks

In addition to the more comprehensive SV calls on the normal assembly (Supplementary Table S9), we created a somatic SV and CNV benchmark on GRCh38 with deletions, duplications, insertions, VNTR expansions and contractions, and complex translocations and inversions (Supplementary Fig 26–28 and 31–35). Important for use of this benchmark, v5 of the SV comparison tool truvari was developed to account for differences in how tools represent SVs as BNDs vs INV, DEL, and DUP. We evaluated the reliability of the GRCh38 SV benchmark by comparing 4 SV callsets from short and long reads made before availability of HG008 benchmarks, and curating common false positives and false negatives. When comparing four common short and long read-based somatic SV callsets against this benchmark, 16 somatic truncal INS and DEL in tandem repeats were missed by all methods. While SVs detectable on GRCh38 were generally detectable by both short and long reads outside TRs, one of the chr18/chrX balanced translocation pairs was missed by short reads in low mappability regions because the chr18 breakpoint was inside an L1HS and the chrX breakpoint was in an Alu between LTRs. In addition, short reads supported all L1 insertions except a subclonal L1 insertion inside a reference L1, but did not resolve the sequences of the LINE insertions. Common sources of false positives across all callsets included BNDs and DELs in tandem repeats and segmental duplications, often related to germline SVs. With availability of more comprehensive benchmarks like this, callers can improve in filtering false positives and calling SVs in repetitive regions.

Based on the small variants on the normal assembly (Supplementary Table S10), we developed a benchmark for truncal SNVs and INDELs on GRCh38, enabling reliable identification of false positives and false negatives in challenging regions, including homopolymers, tandem repeats, and segmental duplications (summaries, benchmark development, and curation examples in Supplementary Fig 36–41, 49–52). FP SNVs were highly enriched in segmental duplications and tandem repeats, while FP indels were most enriched in homopolymers and tandem repeats (Supplementary Table S11 and Supplementary Fig 36). While FN SNVs were common in all three repeats, FN indels were most enriched in homopolymers and tandem repeats. Since these repetitive regions were largely excluded in previous benchmarks, this benchmark enables developers to improve and demonstrate accuracy in more challenging regions.

## Discussion

We demonstrate that complete assembly of all chromosomes except most rDNA arrays in an early-passage hypodiploid tumor cell line is possible. This assembly uncovers distinct centromeric structures in rearranged chromosomes as well as many small and large truncal somatic variants in repeats not detected by current reference-based methods. Even though this pancreatic cell line has different mutational signatures than a recently characterized melanoma cell line with a DSA of the normal,^2^ we find similar enrichment of somatic variants in centromeric satellites (particularly CDR and HSat2) and telomeres. While short reads detect most large somatic SVs in non-repetitive regions,^4^ the assemblies reveal many SVs in repetitive regions, which even long reads miss most SVs in these regions with standard mapping-based approaches. More recent work showed that some of these SVs in repeats can be detected when assembling only the normal, but this normal DSA approach still can miss some SVs like large duplications/insertions in the centromere, and phasing and disentangling complex SVs is challenging. In addition, we find that the tumor assembly can help close gaps and correct errors in the normal assembly, particularly in regions with LOH or when ultralong reads only exist for the tumor cell line. Beyond SVs, while previous work has shown higher concordance for small variants, we find that polishing assemblies with high-accuracy reads like Element or Onso enables resolution of two times more small indels in homopolymers and TRs that are challenging to detect without the paired tumor and normal assemblies, even in this tumor without microsatellite instability. While most homopolymers occur outside coding regions, accurate calling of these can be useful for lineage analysis.^41^ In addition, somatic variants can appear to be SNVs when they are actually indels, or small indels when they are actually SVs, due to germline variation with respect to a non-DSA reference. 44% of small variants on GRCh38 have representations differing from the true somatic change, affecting kataegis and omikli estimates and mutational signatures. These variants pose representation challenges and a need for standards for representation and tools to compare these variants across callsets and individuals. Ideally, somatic variants are called against the DSA rather than a reference like GRCh38 to avoid these representation challenges and enable robust somatic calling in repetitive and highly variable regions.

Stable persistence of dicentric and fused centromeres across passages, together with critically eroded telomeres and chr13q/chr15p telomeric translocations, suggests this tumor has reached a structural endpoint of the breakage-fusion-bridge cycle. The putative kinetochores of the chr6/chr7 fusion were 1 Mbp apart, similar to the chr1-chr19 translocation observed in oligodendroglioma^42^ and dicentric Robertsonian translocations.^43^ In another case not previously identified, a single putative kinetochore was derived from kinetochores from two different chromosomes resulting in a fused centromere. Three pseudodicentrics exemplify different mechanisms for inactivation of one centromere in detail: deletion due to an interchromosomal translocation, deletion due to a translocation-associated inversion, and/or methylation. The tumor and normal assemblies also enabled discovery of exceptionally large somatic insertions in centromeric satellites caused by tandem duplications that are challenging without a tumor assembly, with insertions up to 7 times larger than the 19.4 kbp largest centromeric germline de novo insertion found in assemblies of a 17 member pedigree^30^. Although the assemblies precisely resolved all translocations causing large copy number or structural changes, including complex SVs in centromeres, additional insertions and deletions may exist in the centromeres that are not fully resolved in the normal assembly. While centromeres exhibit low recombination and instability in meiosis, these findings, alongside recent analysis of a melanoma cell line,^2^ point to centromeres as regions of genomic instability in cancer. More experiments and cancer types will need to be analyzed to assess the functionality of the novel centromeres, but here we establish the structural and epigenetic substrate to assess this..

Similar to the recently developed ‘genome benchmarking’ approach where the HG002 normal assembly was used as a benchmark instead of benchmarking variant calls, this paired tumor-normal assembly could serve as a prototype for developing genome benchmarking methods for somatic genomes. However, this work has several notable limitations. It is a single hypodiploid tumor cell line, and every tumor genome is different, even within a single tumor, so additional tumor assemblies are likely to yield more findings. Hyperdiploid assemblies will likely require more assembly graph curation and/or new methods. Duplicated regions are more challenging to polish, so hyperdiploid genomes will also need modified polishing approaches. Most acrocentric rDNA arrays were not assembled, but we were able to assess changes in variation and methylation in the tumor rRNA regions, pointing to a substantially altered rRNA template pool in the tumor. Here, we focus on truncal mutations to establish a baseline for future work on subclonal analyses, including analyzing cell lines derived from HG008 single cell clones. In contrast to benchmarks for which subclonal variants are typically the most challenging, this comprehensive, curated benchmark resource for cancer genomics contains challenging truncal variants that constitute more than one third of small indels, large insertions and deletions, and translocations. While the paired normal tissues for HG008 enabled us to avoid normal cell line artifacts previously observed and measure methylation in primary tissues, the quantity of normal tissues is limited. Our approach using the tumor assembly to improve the normal assembly might apply in clinical contexts where normal tissue is limited. In the future, it will be important for the community to develop new tumor cell lines with normal cell line pairs and broad consent for genomic data sharing and cell line distribution, enabling ongoing improvement of somatic calling in challenging regions.

## Methods

### Cell line availability and consent

The HG008-T tumor cell line is publicly available as CRL-3734 from ATCC. The ATCC cell line is derived from the same passage 13 stock as the MGH passage 23 cells (the primary source of data for this work) and the NIST passage 21 and 41 cells (used to confirm truncal variants). Therefore, we expect that truncal variants should generally be observed in cells from all sources, but subclonal variants will differ. Normal cell lines are not available - only data from normal pancreatic and duodenal tissues.^14^ Collection of tissue and blood from patients with pancreatic disease was approved by the Mass Brigham General IRB under protocol # 2003P001289. The MGH Data and Tissue Sharing Committee and NIST Research Protections Office determined that the signed consent explicitly allows for sharing of the participants’ genomic data and development and distribution of cell lines. Relevant excerpts from the patient consent were published with the previous data for HG008.^14^

### Strategy for assembling the tumor and normal genomes

We hypothesized that a near-complete assembly of the average cell in this PDAC tumor cell line may be possible because most large somatic CNVs were deletions, with only a small number of large duplications, and most SVs were truncal or in a small fraction of cells. Accordingly, we adapted the process used in recent T2T normal genome efforts (Verkko-based assembly with PacBio HiFi, ONT, and Hi-C reads) to generate haplotype-resolved assemblies of the normal and average tumor genomes. We had to make some adjustments to the recipe because only the tumor had ultralong ONT reads due to degradation of DNA in the normal tissues.

### Initial Assembly Using Verkko

We used verkko v2.2 and v2.2.1 to assemble normal and tumor genomes, respectively, using HiFi and ONT reads with Hi-C for phasing. To correct errors in ONT reads prior to assembly, we used HERRO^44^ (https://github.com/lbcb-sci/herro) so that corrected ONT reads could be used by verkko to build the graph under the –hifi parameter, with uncorrected reads input as well under the --ont parameter. The version of HERRO used was the debug branch (commit on April 22, 2025) on the HERRO github repository. The Minimap2 parameters used for overlapping were -K8g -cx ava-ont -k25 -w17 -f 0.005 -e200 -r150,150 -z200 -m<X> where <X>=1500 for the tumor sample and <X>=100 for the normal sample. The command used for running the verkko assembler was like “verkko -d verkko_asm_out --hifi hifi.fq.gz ont_HERRO.fa.gz --hic1 HiC-R1.fastq.gz --hic2 HiC_R2.fastq.gz –nano ONT.fq.gz --unitig-abundance 7”. Contiguity of automated assemblies of HG008 was limited by lack of ultra-long reads in the normal and by large somatic duplications and subclonal SVs in the tumor. Of the 46 normal chromosomes, verkko output 32 T2T scaffolds (4 T2T contigs), and of 35 tumor chromosomes, verkko output 21 T2T scaffolds (15 T2T contigs).

### Manual Resolution of Tumor Assembly Graph

To resolve large duplications and achieve a near-complete tumor assembly, we curated the assembly graph. Most tandem duplications and the 23 Mbp duplication of the start of chr1 were resolved automatically by verkko with the ultralong reads. We manually resolved 3 large somatic tandem duplications larger than 1 Mbp by going around loops in the graph twice, randomly selecting which path in each bubble comes first when these variants between duplications were not automatically phased by verkko (Supplementary Fig 53). This choice means these smaller somatic variants may be on the wrong copy of the duplication, but when the tumor assembly is aligned to the normal assembly or the reference genome, somatic variants will still be called accurately. Variants were similarly assigned randomly on the very large 83 and 64 Mbp duplications of chr17 and chr20 if they were not phased by the assembler. We also needed to manually resolve paths due to a subclonal translocation connecting most of the chrX-chr18 hybrid to the chr2-chr20 hybrid (https://github.com/jzook/HG008SVcuration/issues/101). While this subclonal variant is in about half of the passage 23 batch of cells used for most of our sequencing, it is not detectable in other passages, so we chose the path without this variant for our tumor assembly, and output the subclonal chr2-chr20-chrX-chr18 hybrid chromosome in a separate fasta file that is not used further in this work. This subclonal translocation joins chr2_hap2 to chrX_hap1 so it causes a challenge in our phasing convention, since for truncal translocations we were able to arrange the haplotypes in the normal and tumor such that they match (except for chr19 where the tumor chromosome combines both haplotypes). This curation, as well as manual removal of some erroneous sequences after the telomeres, resulted in all 35 chromosomes being T2T scaffolds and 28 T2T contigs, with only 9 gaps, with all in acrocentric short arms around rDNA except one in chr18_chr20_hap1. The chr15_hap2 rDNA array was assembled because it was a single monomer, but other rDNA arrays contained gaps in the assembly. While not experimentally confirmed, the assembler uniquely scaffolded the telomeric distal bits for the acrocentric short arms in the tumor, except for the highly identical chr14 and chr15, which were manually resolved as described above.

For regenerating the assembly sequences after each round of manual curation, we created a path file (manual.paths.gaf), and the command for re-running verkko was like “verkko -d verkko_asm_out_curated --assembly verkko_asm_out --paths manual.paths.gaf --hifi hifi.fq.gz ont_HERRO.fa.gz --hic1 HiC-R1.fastq.gz --hic2 HiC_R2.fastq.gz –nano UL-ont.fq.gz --unitig-abundance 7”, as suggested by the verkko developer.

### Manual Resolution of Normal Assembly Graph

The automated normal assembly contained many gaps due to the lack of ultralong reads. To improve the normal assembly, we aligned the tumor assembly to the normal assembly graph using GraphAligner v1.0.20. We then manually chose paths in the normal matching haplotypes present in the tumor, except where somatic structural variation occurred. The paths of the tumor assembly through the normal graph resolved many gaps where the phasing had been ambiguous or the tangle had been too complex without long reads, and it also corrected phasing that verkko had assigned incorrectly without the additional information from the tumor. We manually chose the paths to avoid incorporating somatic variation from the tumor into the normal assembly. Generally, somatic SVs were clear because they caused breaks in the alignment of the tumor to the assembly graph. We also scaffolded the normal acrocentric short arms based on the tumor, except for chr21_hap2 and chr22_hap1, which were deleted in the tumor and lacked sufficient evidence in Hi-C to scaffold.

Note that we could not resolve all regions in the normal assembly that were missing from the tumor, so more errors and gaps remain in them, but they do not affect the discovery of somatic variants. This curation, as well as manual removal of some erroneous sequences after the telomeres, resulted in 44 of 46 chromosomes being T2T scaffolds and 23 T2T contigs, with only 53 gaps, mostly in centromeres and rDNA tangles. We confirmed the structural and phasing accuracy of the assemblies by mapping Hi-C reads to the combined haplotypes, which demonstrated much higher connections within each haplotype-resolved chromosome (93.5% for normal and 94.2% for tumor) than between haplotypes or chromosomes, with no evidence of large structural or phasing errors (Supplementary Fig 3).

### Polishing

After manually resolving structural issues in the assemblies, we polished the assemblies by aligning short insert and long insert Element, HiFi, and ONT reads to the assemblies and calling variants with DeepVariant. The use of HERRO-corrected ONT and HiFi reads to form the consensus sequence may have resulted in increased homopolymer errors. HiFi and uncorrected ONT reads were mapped only to the combined haplotypes, while short and long insert Element reads were mapped to the combined haplotypes as well as to each haplotype separately because the shorter reads often cannot be mapped uniquely to one haplotype. Homozygous variants with GQ>4 and coverage less than two times the mean were candidates for correction, with additional filtering for long reads. Variants in homopolymers longer than 6 bp and dinucleotide TRs longer than 11 bp, as well as 1 bp and 2 bp indels and GQ<10, were filtered for ONT due to its higher error rates. HiFi variants were ignored in homopolymers longer than 6 bp and dinucleotide TRs longer than 11 bp if any Element callset had a 1 bp to 4 bp indel call, since Element is more accurate for small indels in these repeats if it is mappable. Finally, calls from all technologies were merged, calls within 5 bp of a somatic SV from the tumor to normal svim-asm calls were removed, and the remaining edits were applied to the assembly using bcftools consensus. The same process was applied for both tumor and normal assemblies.

We made 204,640 (552 SNV, 204,071 indel, and 17 SV) and 164,093 (694 SNV, 163,368 indel, and 31 SV) corrections to produce the v6.2 normal and v3.1 tumor assemblies, respectively, by aligning short and long-insert Element, PacBio HiFi, and ONT reads to the combined haplotypes, and Element to separate haplotypes. 99.6 % and 99.4 % of corrections were in homopolymers or diTRs, mostly from Element reads, which are more accurate in long homopolymers. While most errors in long homopolymers do not impact QV because they often do not contain unique *k*-mers and sequencing reads are noisy, the QV still increased from 54 to 59 using Illumina 31-mers or 65 to 66 using PacBio HiFi 31-mers. These QVs are near the limits of the utility of *k*-mer-based approaches, as many remaining *k*-mer errors were in regions with technology dropout. The lowest quality regions in the tumor assembly were those with highly identical somatic duplications, since short reads and most long reads cannot map uniquely to each copy. When identifying putative somatic variants by mapping the tumor to normal assemblies, the largest improvement after polishing was for 1 bp insertions, decreasing from 194,000 to 19,000, and 1 bp deletions, 2 bp insertions and deletions, and 3- to 49 bp insertions also decreased more than two fold. In addition, curation of variants called from Onso, Element, PacBio HiFi, and ONT reads aligned to their respective polished assemblies indicated most were not errors in the assembly, though we conservatively use some of these calls to exclude regions with potential errors or subclonal variation from the small variant benchmark.

### Checking Assembly Completeness

When using compleasm^45^ (https://github.com/huangnengCSU/compleasm, v0.2.7) with the primate gene database containing 11,834 genes^30,46,47^, the combined normal haplotypes have no missing or fragmented genes, 4 genes are on only one haplotype, 85 and 78 genes are duplicated and 89 and 91 genes have frameshift mutations on haplotypes 1 and 2, respectively. The genes missing from one normal haplotype were *STX1* (which appears correct in both haplotypes), C2orf80 (which has a true 25 kbp deletion on hap2), and *PPP1R12A* (which has a gap in hap1). *PLIN4* is fragmented because it has a true 396 bp deletion in an exonic TR. The highly polished HG002v1.1 assembly has similar numbers of genes with duplications (76 and 83) and frameshifts (90 on each). The combined tumor haplotypes are missing one gene entirely due to deletions of *CDKN2A* on both haplotypes and have one fragmented gene due to a translocation in *KDM2B* and deletion of the other haplotype, with about half of genes on only one haplotype due to large CNVs, and 136 genes have frameshift mutations. NucFlag identified potential errors with PacBio HiFi reads, which were fixed with a hifiasm assembly in the final v6.3 normal and v3.2 tumor assemblies when the hifiasm assembly reduced errors. In the final assemblies, NucFlag statistics are in Supplementary Tables S13 and S14, and HMM-Flagger statistics are in Supplementary Table S15, with most errors identified around assembly gaps. Finally, we used KaryoScope to assign each contig to its chromosome(s) of origin and to order and orient them based on CHM13 (Fig. 1a and Supplementary Fig 54). For tumor chromosomes derived from more than one chromosome, we include all chromosome numbers in the name, and orient by the largest sequence.

### Handling unassigned contigs

We removed the remaining unassigned contigs from the tumor assembly because these were redundant sequences or rDNA, but we kept 18 unassigned contigs with nonredundant sequence for the normal assembly, with 16 mostly centromeric contigs ranging in size from 111 kbp to 2.2 Mbp, and 2 contigs for the acrocentric short arms of chr21_hap2 and chr22_hap1. To assign potential chromosomes to the centromeric contigs, we mapped the unassigned contigs from HG008N_v6.3 to all 466 HPRCv2 haplotype assemblies using minimap2 (v2.30, asm5 preset). For each contig, haplotype, and chromosome, we merged overlapping query-coordinate intervals to compute query coverage fraction, and separately computed a mean gap-compressed identity weighted by alignment length. For each contig and chromosome, we then computed the product of these two values per haplotype (0 if the contig had no alignments to that chromosome in a given haplotype) and ranked chromosomes by the mean of this score across all 466 haplotypes. Contigs were considered assigned to the top-ranked chromosome when at least 95% of haplotypes agreed on that chromosome (haplotype unanimity ≥ 0.95) and mean score ≥ 0.60. 13 contigs were assigned (Supplementary Table S12).

### Matching tumor haplotype assembly to normal haplotype assembly

The tumor contigs and scaffolds exactly match the chromosomes found in most cells with dGH SCREEN and traditional karyotyping (Fig. 1a). All large structural events found by dGH SCREEN in >80 % of cells are recapitulated in the tumor assembly, and other events were in only 1 or 2 of 80 cells. We also manually separated the assemblies into haplotype 1 and haplotype 2 to ensure the haplotype chromosome numbers of the tumor and normal are the same. The matched tumor and normal haplotypes were confirmed by haplotype-specific markers (Supplementary Fig 2c). This matching of haplotypes was possible for all truncal events in HG008 except the true haplotype switch on chr19. Interestingly, this matching would not be possible for all tumors and would not be possible for a subclonal chr2_hap2-chrX_hap1 translocation that occurs between different haplotypes in HG008 (https://github.com/jzook/HG008SVcuration/issues/101). The tumor assembled haplotypes were aligned to their corresponding normal haplotypes, so that differences are putative somatic variants (Fig. 1b). Potential haplotype switch errors or somatic changes were detected by looking for *k*-mers from each normal assembly haplotype in the tumor contigs, and the only evidence for a haplotype switch was in the chr19_chr22 hybrid chromosome that contains both chr19 haplotypes (Fig. 4), as described below. The alignments between tumor and normal haplotypes form the basis for comprehensive truncal somatic variant calls.

### Comparing normal haplotype assemblies to T2T-CHM13v2.0

The normal haplotype assemblies were compared to T2T-CHM13v2.0 using SyRI to illustrate the high level structural difference (Supplementary Fig. 2a). A known large polymorphic inversions on the p-arm of chromosome 8 for both HG008_hap1 and HG008N_hap2 were observed, which is similar to that in HG002^6^. Similar inversions were found in haplotype assemblies of chr9, chr15, and chr16. When matched tumor haplotype assemblies were aligned onto their respective normal assembly, we noticed that large inversions exist on tumor chr3_hap1, chr18_hap1, chr5_hap2, and chr7_hap2 (Supplementary Fig. 2b).

### Subclonal whole genome duplication

Karyotyping and dGH show that some cells have experienced a whole-genome duplication event (i.e., at some evolutionary epoch all chromosomes then present were duplicated), and almost all cells have some unique large copy number variation. While the fraction of whole genome doubled cells is low at the passages sequenced here (9 of 40 cells at passage 21), it increases with passages, making up 28 of 40 cells at passage 53, and 20 of 20 cells at passage 101. Because whole genome doubling and low frequency CNVs occurred more recently and vary between passages, we do not include these in our truncal benchmark, and normalize copy number to the hypodiploid state.

### Computational Karyotyping with KaryoScope

KaryoScope is a k-mer-based computational karyotyping framework that classifies assembled genome sequences in an alignment free manner to determine chromosomal composition and annotate structural features.^17^ We applied KaryoScope to both the normal and tumor assemblies to assign each contig to its chromosome(s) of origin and to order and orient them. KaryoScope uses a reference k-mer database derived from CHM13 to classify query sequences by chromosomal origin and annotate cytogenetic features such as centromeres, pericentromeric satellites, and subtelomeric repeats. Contigs containing interchromosomal rearrangements were assigned to all constituent chromosomes. To compare computational and cytogenetic results, we generated a figure displaying KaryoScope haplotypes as colored bars representing chromosomal composition alongside KromaTid dGH microscopy images for each chromosome, with ISCN annotations for both methods.

### Hybridizatio-based Karyotyping with KROMASURE SCREEN

Whole-genome cytogenetic structural variant analysis was performed using directional genomic hybridization (dGH) SCREEN to characterize chromosome-scale structure in a hypodiploid pancreatic tumor cell line (HG008-T) across early and late passage conditions, enabling assessment of both shared clonal architecture and passage-associated structural variation.

SCREEN is a single-cell, strand-specific cytogenetic method that enables genome-wide detection of numerical and structural chromosomal variation, including inversions, translocations, insertions, copy number alterations, and complex rearrangements. The method selectively removes one DNA strand prior to probe hybridization, enabling differential labeling of sister chromatids and direct visualization of strand orientation. This strand-specific resolution enables detection of intrachromosomal rearrangements, such as inversions, that are not readily resolved by conventional cytogenetic or sequencing-based approaches (Ray *et al.*, 2013; Robinson *et al.*, 2019, Cross and Bailey, 2026).^15,48,49^

Cells were cultured and harvested using standard cytogenetic procedures with incorporation of halogenated nucleotide analogs for one cell cycle, followed by mitotic arrest and metaphase preparation. Metaphase spreads were quality-filtered based on chromosomal morphology, hybridization performance, and resolution prior to analysis.

All human chromosomes were simultaneously assessed using a five-color high-density paint configuration. Individual metaphase cells were imaged, assembled into karyograms, and analyzed at single-cell resolution. Structural events were scored per cell and classified as whole-chromosome gains or losses, inversions, interchromosomal rearrangements, large deletions including truncations and homolog size asymmetry, sister chromatid exchanges, and complex multi-breakpoint events. Structural variation was detected at an effective resolution of approximately 20-30 kb.

Interpretation of structural events incorporated prior cytogenetic characterization, including features established from G-banding datasets, together with an inverted DAPI channel to support chromosome identification, banding context, and breakpoint localization, particularly in regions of high structural complexity performed in concert with the SCREEN analysis.

To resolve complex and multi-chromosomal rearrangements, targeted follow-up hybridizations were performed using modified paint configurations in which subsets of chromosomes originally labeled with the same fluorophore were separated into distinct color channels. These follow-up assays were performed on the early passage sample and enabled unambiguous identification of chromosomal partners and source material within complex rearrangements, including marker chromosomes and inserted segments that could not be fully resolved using the standard five-color configuration. Information derived from these targeted analyses was incorporated into final structural annotations across both early and late passage datasets. Two modified screen assays were run with the following configurations: Modified assay #1 involved Chr.1 (yellow),Chr.3 (orange),Chr.7 (red), and Chr.19 (green). Modified assay #2 involved Chr.6 (yellow), Chr.11 (red), Chr.12 (green), Chr.18 (aqua), Chr.20 (orange). Events resolved by modified dGH SCREEN assay analysis are incorporated into the final annotations and include the removal of the “?” from the following preliminary calls: der(3)t(3q-11q-6q), der(5)t(1q-5q), der(6)t(7p-6p;11p-6q)inv(7(p), der(11)t(3q-11q), t(12;15)(15q-12q), t(Xq-18q), and der(22)t(19-22p).

Per-cell event calls were aggregated to generate population-level structural profiles, enabling quantification of clonal and subclonal variation across the cell population. The single-cell nature of the assay enables direct assessment of structural heterogeneity and identification of recurrent chromosomal configurations consistent with shared clonal ancestry.

SCREEN analysis was used to define chromosome-scale architecture and to validate structural features observed in haplotype-resolved tumor genome assemblies. Structural rearrangements identified by SCREEN were compared with sequencing-based, assembly-derived karyotypes to assess concordance in chromosome composition, translocation partners, and large-scale structural organization. Because SCREEN directly interrogates intact metaphase chromosomes, it provides an orthogonal measurement to sequencing of genome structure that is independent of sequence alignment and enables detection of rearrangements in repetitive and centromeric regions that remain challenging for sequence-based approaches.^50,51^

SCREEN analysis of the HG008-T pancreatic tumor cell line revealed a hypodiploid genome characterized by widespread chromosome arm loss, consistent with prior karyotyping and sequencing-based observations. Across analyzed metaphase cells, the majority of structural features were shared across cells, indicating a dominant clonal architecture, while additional variation was observed at lower frequency, consistent with subclonal diversification and passage-associated evolution.

Chromosome-scale losses were prevalent across the genome, frequently involving partial or complete loss of chromosomal arms. In a subset of cells, these losses co-occurred with genome doubling, resulting in mixed ploidy states across the population. Consistent with the underlying dataset, the proportion of genome-doubled cells increased substantially between early and late passage samples, reflecting expansion of this population during in vitro propagation. In addition to numerical abnormalities, extensive structural variation was observed across multiple event classes, including interchromosomal rearrangements, inversions, and complex multi-breakpoint configurations. Recurrent rearrangements involved multiple chromosomes, including chromosomes 1, 3, 6, 11, 12, 15, 18, 19, and 22, with frequent formation of derivative and dicentric chromosomes. These events were consistent with a highly rearranged but structured tumor karyotype, rather than random genome fragmentation.

Comparison of early and late passage samples demonstrated that the majority of large-scale structural features were conserved across passages, consistent with a shared clonal origin. Superimposed on this clonal backbone, additional lower-frequency structural and numerical variants were observed, with many events present in only one passage condition, consistent with ongoing genomic instability and selection during culture. Multiple complex chromosomal configurations were identified, including rearrangements involving three or more chromosomes and the presence of marker chromosomes with ambiguous origin based on conventional karyotyping. To resolve these events, targeted follow-up hybridizations using modified paint configurations were applied to deconvolute chromosomes initially labeled within the same fluorophore group. This approach enabled direct identification of chromosomal partners and assignment of source material within complex rearrangements, resolving previously ambiguous structural calls and refining the composition of derivative chromosomes.

All dGH results are available at https://ftp-trace.ncbi.nlm.nih.gov/ReferenceSamples/giab/data_somatic/HG008/NIST/HG008-T_bulk/20240508p21/KromaTiD-dGH_20241029/.

### Centromere mapping and annotation

Centromeres are specialized chromatin domains that mediate the segregation of chromosomes to daughter cells during cell division. In humans, centromeres are composed of near-identical tandem repeats known as α-satellites, which are organized in a head-to-tail fashion and assembled into arrays of higher-order repeats (HORs) that can span several million basepairs. The epigenetic landscape of centromeres is critical to their identity and function, particularly a 75-300 kbp hypomethylated region, known as the “centromere dip region” (or CDR^52,53^), enriched with nucleosomes containing the histone H3 variant CENP-A.^30,53,54^ This combination of hypomethylated DNA and CENP-A chromatin is thought to define the site of kinetochore assembly during mitosis and meiosis, resulting in spindle microtubule attachment during metaphase and subsequent segregation of chromosomes to daughter cells during cell division.^55^

Although the genetic and epigenetic landscapes of human centromeres have been well-defined in the healthy population,^30,46,47^ they have not yet been completely assembled in any tumor cell line to our knowledge. Therefore, we sought to determine the complete genetic and epigenetic landscape of centromeres in both the normal and tumor samples and assess the events that may have led to changes within centromeres. We first identified each centromere within the normal and tumor genome assemblies using CenMAP,^30^ successfully identifying all 46 centromeres in the normal sample and all 35 centromeres in the tumor sample. The reduction in chromosome number in the tumor sample suggested chromosomal fusion events at or near centromeres, forming chromosomes with two putative functional centromeres (termed a dicentric). To test this hypothesis, we applied our centromere mapping and annotation pipeline (CenMAP^30^), which we refined to be able to detect chromosomes with more than one centromeric α-satellite HOR array.

To identify and annotate centromeric regions in the HG008 normal (HG008-N) and tumor (HG008-T) genome assemblies, we applied CenMAP, a centromere mapping and annotation pipeline (https://github.com/logsdon-lab/CenMAP; v1.2.0, commit c439499ca94227685e074256a102673f49eea400),^30^ which automatically identifies α-satellite-containing regions in a whole-genome assembly, annotates the sequence and structure of the centromeric region, determines the length of the active α-satellite HOR array, defines the location of the CDR, and generates plots for visualization. CenMAP was updated specifically for this study (v1.0 and greater) to enable the detection of multiple α-satellite-containing regions from different chromosomes on the same contig, as observed in the HG008 tumor assembly.

To detect α-satellite-containing regions, CenMAP (v1.0 and greater) runs three tools: K-Mer Counting^56^ (KMC), Satellite Repeat Finder^57^ (SRF), and Tandem Repeat Finder^58^ (TRF). First, CenMAP identifies repetitive sequences in a whole-genome assembly by identifying k-mers (k = 171 bp; the average length of an α-satellite repeat) that occur more than 10 times using KMC 3 (v3.2.4; Kokot et al., Bioinformatics, 2017). Then, satellite motifs are constructed using SRF^57^ (commit e54ca8c8eccf6b1f19428b0f862f2c90575290a0), and tandem repeats are annotated in each motif with a fork of TRF (trf-mod; commit 3e891db310124f7e5f7a630a1c006650be9d1f3a; github.com/lh3/TRF-mod). Using a custom script we developed to work with both SRF and TRF outputs, srf-n-trf (v0.1.1; https://github.com/koisland/srf-n-trf), we remove any SRF motif without a TRF motif and with a length of n x 170 bp, where n was <= 5 for α-satellite monomers or <=42 for HSat1A monomers^59^ within 2% difference in length using the following command: srf-n-trf motifs -f ${srf_motifs} -m ${trf_monomers} -s 170 340 42 -d 0.02. With a candidate set of motifs, CenMAP maps each assembly to the motifs using minimap2^60^ (v2.29) with the following command: minimap2 -c --eqx -N 1000000 -f 1000 -r 100,100 <(srfutils.js enlong ${srf_motifs}) ${assembly.fasta}. The output PAF is then filtered to find monomers from aligned regions or CIGAR elements that contain an α-satellite or HSat1A monomer with the command: srf-n-trf monomers -p ${paf} -m ${trf_monomers} -s 170 340 42 -d 0.02 --max-seq-div 0.3. All α-satellite and HSat1A regions are iteratively merged with srf-n-trf regions if they are within 100 kbp of each other and contain at least one monomer using the following command: srf-n-trf regions -b ${monomers.bed} -m 30000 -d 100000 -s 170 340 --diff 0.02. This yields the coordinates of the centromeric α-satellite HOR array(s) greater than 30 kb. The coordinates of the α-satellite HOR array(s) are subsequently extended on either side by 1 Mb using BEDtools slop, producing the coordinates of the centromeric regions within each contig.

Following the detection of candidate centromeric regions, CenMAP then assigns chromosome names to each contig. CenMAP aligns each whole-genome assembly to the centromeric regions of the CHM13 reference genome (v2.0) using minimap2 (v2.28) with the following parameters: -x asm20 --secondary=no -s 25000 -K 15G. The resulting BAM files are then converted into BED files using rustybam (v0.1.34).^61^ Any contigs containing centromeric regions are reoriented from p to q arm relative to the CHM13 reference genome with the HG002 Y chromosome assembly (v2.0). Finally, contigs are renamed to indicate the chromosome they most accurately align to based on the percent of sequence aligned to the CHM13 reference genome (v2.0), with a minimum of 5% required.

To annotate centromere sequence, structure, and epigenetic features, CenMAP uses ModDotPlot^62^ (Implemented in CenStats v0.1.3), Repeatmasker^63^ (v4.1.2p1), HumAS-SD (https://github.com/logsdon-lab/Snakemake-HumAS-SD/tree/feature/multi-chr-lib; commit: 52455f930ddc1615b2751d7a0ca546232f9e7aa3), and CDR-Finder^24^ (commit: 18a8dc62523a2de75bdbce136561ce1a9ce6fed9; https://github.com/logsdon-lab/CDR-Finder). We modified the original HumAS-SD implementation to construct monomer libraries with multiple chromosomes in cases of chromosomal rearrangements within the centromere. In such situations, a mixed monomer library is built from the base monomer library to avoid incorrect/missing annotations.

### Centromere breakpoint analysis

To detect and validate centromere fusion or recombination breakpoints, both assembly- and read-based evidence were evaluated. First, we aligned the HG008 tumor assembly to the normal assembly using a custom mapping pipeline (https://github.com/koisland/asm-to-reference-alignment; commit d3610a8f8c4a3fddedb7b64078ffb65fe0853573), default minimap2 parameters (-x asm20 --secondary=no -s 25000 -K 8G -a --eqx --cs), and rustybam trim and break PAF parameters (rb trim-paf {input.paf} | rb break-paf --max-size 10000). The resulting PAF and trimmed PAF were used with SVbyEye^64^ (commit ea6d0ddef601fdb029d88cfeec66537809ac96b4) and SafFire^61^ (commit 63596fa6426b4562a9625077fde2300e567eeece), respectively, for visualization of centromeric breakpoints and homologous regions. Next, read alignments from publicly available PacBio HiFi and ONT datasets were used to determine exact breakpoints and manually inspected through IGV^65^ (v2.19.1). IGV sessions and screenshots can be found at https://github.com/koisland/HG008_TN_analysis/tree/main/data/igv.

Visualization of both tumor and normal centromeric regions and their breakpoints was performed with a modified fork of ModDotPlot^62^ v0.9.9 that allows for more colors and fixes custom breakpoints (https://github.com/koisland/ModDotPlot; commit: 3c174a4dbdab86a46f2ad9793238ca4e1705e838). Non-centromeric regions associated with centromeric translocation breakpoints were subset with SAMtools^66^ faidx (v1.23) and/or centromeric regions were taken directly from CenMAP’s output and concatenated. ModDotPlot was run with the following command: moddotplot static --compare-only -f ${multifasta} -o ${outdir} -id 70.0 -w 5000 --breakpoints ${breakpoints} --colors ${colors}. Combining these plots with those generated by CenMAP in Adobe Illustrator produced the final figures.

### NucFlag

To detect potential assembly errors in HG008-T and HG008-N with NucFlag, we first aligned both PacBio HiFi or ONT reads to their respective whole-genome assembly using minimap2 (v2.30; Li et al., Bioinformatics, 2018) with the following parameters: -a -x lr:hqae -y --eqx --cs -I 8g. Alignments were filtered using SAMtools^66^ v1.22 and the filter flag (-F) 2308 to remove secondary, supplementary, and low-quality alignments. Next, we ran NucFlag (v1.0.0-a3) with the following command: nucflag call -i ${read_alignments.bam} -f ${assembly.fasta} -p ${num_of_processes} -t ${num_of_threads} -x {ont_r9 or hifi} -o ${output.bed}. NucFlag uses peak detection in the coverage, mismatch, insertion, deletion, and softclip signals to detect collapsed and misjoined sequences as well as insertions, deletions, softclipping, low-quality regions, mismatches, and heterozygous sites within each genome assembly. In addition, NucFlag v1.0 annotates specific repeat-associated errors associated with long-read technologies, such as homopolymers and dinucleotide repeats, to allow downstream filtering.

### Constructing Hi-C contact matrices

We construct Hi-C matrices to observe support for large SVs (Supplementary Fig 12) by aligning the raw arima paired end reads of both the tumor and the matched normal tissue onto the GRCh38 reference assembly using the dockerized pipeline (https://github.com/4dn-dcic/docker-4dn-hic) provided by the 4DN consortium. The pipeline generates .pairs files for both the tumor and the matched normal that we further process with Juicer^67^ (https://github.com/aidenlab/juicer) to produce .hic files. To visualize Hi-C matrices we use the Juicebox toolkit (https://aidenlab.org/juicebox/).

### Tumor/normal haplotype assembly-based somatic SV calling

For tumor-normal haplotype assembly-based somatic SV identification, we used SVIM-asm,^68^ SyRI,^69^ and PAV^70^ to generate initial SV sets. We also selected mapping-based calls from severus,^12^ savana,^71^ cutesv,^72^ nanomonsv,^73^ and sniffles2^74^ to ensure the assemblies did not miss variants. We manually inspected each of those candidate SVs using IGV and svviz2,^75^ and retained likely truncal SVs with supporting reads in the tumor but not in the normal.

We lifted the tumor vs. normal assembly-based SV breakpoint regions to the GRCh38 reference and curated each SV, as well as CNVs caused by them, to develop a confident benchmark for SVs and CNVs with precise breakpoints. To ensure the applicability of the truncal variants across passages, we required support for variants in passage 41 HiFi reads for the truncal benchmark, a passage that originated from the passage 13 stock of cells that we expect all bulk HG008-T cells to have in common in the future. While we include many subclonal SVs in the vcf, we did not focus on characterizing low frequency SVs in this work.

### Structural and copy number variant curation

We used an SV curation process documented as issues in a Github repository (https://github.com/jzook/HG008SVcuration) which involved generating issues for all of the SVs then requesting community members to curate the SVs so that all were curated by at least two individuals. Ribbon and splitthreader^76,77^ were used to identify whether a similar SV existed with a few kb, the SV is a true translocation, it causes a CNV, the SV is part of a complex set of variants within 5 Mbp, and/or the SV is inside a larger CNV or LOH region. Within IGV, we determined if the breakpoints appear exactly correct for the SV. If the variant was an insertion we used BLAT to determine if it came from somewhere else in the genome or is a tandem duplication. Finally, the curator used IGV and ribbon/splitthreader images to determine if the SV appeared to be in all cells or likely to be subclonal. The Github repository has 306 issues and the results of this curation updated criteria for inclusion in the benchmark. We follow the VCFv4.5 specification in describing translocations and complex inversions as breakend (BND) calls with an EVENTTYPE that gives more detail about the type. Variants in VNTRs are represented as close to their true somatic change as possible even if they modified germline variants. In the draft V0.5 SV+CNV somatic benchmark, the FILTER field has values of CENSAT for centromeric and heterochromatic SVs, LT50 for variants close to but smaller than 50 bp, and VAFlt5percent for subclonal SVs at <5% VAF in batch 0823p23. BED files are included that include or exclude subclonal variants with VAF >5%. SVVIZ_VAF_ALL INFO field gives the VAF estimated by svviz2 for combined Element, HiFi, and ONT datasets. The SVVIZBYDATASET INFO field gives the REF and ALT counts and VAF estimated by svviz2 for Element, HiFi, and ONT datasets individually. Several HG8N6.3 INFO fields give calls from tumor vs normal assembly alignments in HG008Nv6.3 coordinates. Phasing information is based on the HG008Nv6.3 and HG008Tv3.1 assemblies.

For somatic CNVs, we curated all SV breakpoints and added CNVs with breakpoints matching the SVs if the SV caused a copy number change. In addition, if a large CNV had a breakpoint in the centromere, we added an SV based on the alignment of the tumor assembly to GRCh38. Unlike somatic breakpoints in the normal assembly centromeres, typically these GRCh38 SVs and CNVs have annotations indicating large uncertainty in the breakpoints because centromeres are not well-represented in GRCh38 and centromeres are highly polymorphic between individuals. Links to curations for all SVs and CNVs are in Supplementary Table S9.

### Somatic LINE insertions

Candidate MEIs from the 50–7,000 bp insertions were manually curated based on alignment to L1HS, AluYa5, or SVA_F mobile element sequences, presence of poly-A tracts, and/or target site duplications (TSDs). Putative source (driver) full-length L1 elements were identified in GRCh38 and CHM13 (HS1) reference genomes using UCSC BLAT and the UCSC Genome Browser, and by interrogating the matched normal VCF. All insertion sequences containing LINE-derived sequences were aligned to a reference L1 element for structural characterization and poly-A tail analysis (Supplementary Fig 46). Mobile element annotation tools used to supplement manual curation were TraFIC,^78^ SVAN (SVAN: https://github.com/REPBIO-LAB/SVAN), and MEIGA (MEIGA: https://gitlab.com/mobilegenomesgroup/MEIGA_LR).

### Truncal small variant benchmark development

To identify true truncal variants, we used repun^79^ to evaluate support for each putative assembly-based somatic variant by short and long reads from both tumor and normal mapped to the normal assembly (Supplementary Fig 41). To be included in the truncal somatic benchmark, we required support in >50% of tumor but <10% of normal reads mapped to combined normal assembly haplotypes with coverage > 5 (ignoring HiFI and ONT for homopolymers covered by short reads, and ignoring Element and Onso for indels >10bp or low MQ). In regions with somatic duplications, we required >30% of tumor but <10% of normal reads. For short reads aligned separately to each haplotype, we required >30% of tumor reads and <10% of normal reads, or >90% of tumor reads and <70% of normal reads to support the variant.

The GRCh38 benchmark is generated by first lifting coordinates to GRCh38. Each somatic variant is given ±50 bp of slop on the assembly, assembly-gap regions are excluded, then coordinates are lifted from the HG008-N v6.3 assembly to GRCh38 using liftOver (minMatch=0.1) with chains derived from minimap2 alignments. Lifted regions are expanded to overlapping repeats. The somatic candidate VCF is split by haplotype and rtg vcfeval (https://github.com/RealTimeGenomics/rtg-tools) is run for each haplotype, comparing HG008-T calls against HG008-N calls. False positives (tumor-unique variants), false negatives (normal-unique variants), and true positives (germline variants shared between tumor and normal) are produced for each haplotype. To generate GRCh38-based somatic variants, vcfeval FP and FN results are filtered to the somatic candidate regions lifted from calls on the HG008N v6.3 assembly, producing the per-haplotype somatic variant set on GRCh38. Regions excluded from the benchmark BEDs include subclonal variants, structural-variant breakpoints (±50 bp), assembly errors (variants flagged by DeepVariant calls on the normal assembly), and complex repeats where the assembly alignment to GRCh38 is unreliable. Phasing information is based on the HG008Nv6.3 and HG008Tv3.2 assemblies.

Three versions of benchmark beds are produced: ‘ll.bed’, ‘ogermlineinterference.bed’, and ‘ogermlinewithin50bp.bed’. HG008-T_somatic_smvar_benchmark_v0.3_all.bed includes all somatic variants that are confidently called, but current comparison tools may not handle different representations of somatic variants that modify germline variants. HG008-T_somatic_smvar_benchmark_v0.3_nogermlineinterference.bed is the all.bed except with regions excluded where somatic variants modify germline variants in a way that current comparison tools cannot resolve correctly. HG008-T_somatic_smvar_benchmark_v0.3_nogermlinewithin50bp.bed is the strictest set: every region in the benchmark is at least 50 bp away from any germline variant. This is not recommended as the primary benchmark bed but rather to understand the effect of germline variants on FP and FN somatic calls.

The v0.1 draft benchmark was compared to nine community-contributed somatic small variant callsets using both aardvark^80^ (https://github.com/PacificBiosciences/aardvark) and rtg vcfeval.^81^ False positives supported by two or more callers were manually curated; some FPs were genuine somatic variants at low VAF that were removed from the benchmark regions as filtered subclonal variants in v0.2, while others resulted from caller errors or representation differences. False-negative rates were stratified by whether the assembly variant has the same or a changed representation on GRCh38; this stratification motivated the multi-tier BED design.

### Mapping & Variant calling for small variant comparisons

Short read data from the different submitters was analysed with community standard computational pipelines to detect somatic variation to build and test the variant benchmarks.

#### OncoAnalyser,

Genomic short reads were analyzed using OncoAnalyser v2.3.0 [https://github.com/nf-core/oncoanalyser/releases/tag/2.3.0]. The nf-core page provides detailed parameter settings, resources, and software versions. Briefly, DNA reads were mapped to the reference genome GRCh38 using BWA-MEM2 and processed downstream using the Nextflow implementation of the open-source, multi-platform Hartwig Medical Foundations tool set WiGITS [https://github.com/hartwigmedical/hmftools. This resulted in a comprehensive (SNV, MNV, CNV, SV, viral integrations) dataset.

#### Ultima Genomics small variant calling:

Ultima genomics short reads were aligned with Ultima Aligner (UA) with default parameters (DockerHub: ultimagenomics/alignment), Somatic short variants were then called using Ultima-adapted somatic deepVariant (https://github.com/Ultimagen/healthomics-workflows/tree/main/workflows/efficient_dv) using the default WGS somatic calling model for matched samples (https://github.com/Ultimagen/healthomics-workflows/blob/main/workflows/efficient_dv/input_templates/efficient_dv_template-WGS-somatic-matched-samples-v1_3-40X-200X.json).

#### DeepSomatic was performed on tumor-normal Illumina and PacBio HiFi reads.

Calls used DeepSomatic^82^ from https://github.com/google/deepsomatic (Docker: google/deepsomatic:1.6.0). Bam files used: https://ftp-trace.ncbi.nlm.nih.gov/ReferenceSamples/giab/data_somatic/HG008/Liss_lab/superseded-2022-data/BCM_Illumina_WGS_20220816/HG008-N-P_Illumina_160x_GRCh38-GIABv3_sorted.bam; https://ftp-trace.ncbi.nlm.nih.gov/ReferenceSamples/giab/data_somatic/HG008/Liss_lab/superseded-2022-data/BCM_Illumina_WGS_20220816/HG008-T_Illumina_185x_GRCh38-GIABv3_sorted.bam; https://nist-giab.s3.amazonaws.com/giab_tumor-normal/incoming/PacBio/Revio/HG008-N-P/analysis/HG008-N-P_GRCh38_GIABv3_37x_PB-HiFi-Revio.bam; https://nist-giab.s3.amazonaws.com/giab_tumor-normal/incoming/PacBio/Revio/HG008-T/analysis/HG008-T_GRCh38_GIABv3_120x_PB-HiFi-Revio.bam

### Small Variant Curation

We used IGV 2.19.7 with a session that contained links to the HG008 v0.2 draft somatic small variant benchmark, one of nine different callsets submitted from collaborators for evaluation, and a variety of short and long read alignments as well as assemblies of both tumor and normal aligned to GRCh38. Using aardvark for comparison of each submitted callset to the draft benchmark, we randomly selected at least five SNVs and INDELs from each set of false positives and false negatives. Also, 5 false negative SNVs and INDELs that were common to all callsets were curated. For a discrepant variant, we examined both the local 48 bp window along with a larger context view of around 10 kb. Along with noting genomic context around the variant, we curated with the goal of determining whether the benchmark variant was correct at a given loci and if the comparison variant was correct or not. The IGV session included 68x HG008-N-D PacBio HiFi, 106x HG008-T PacBio HiFi, 157x HG008-T Ultima, 48x HG008-N-D Pacbio Onso, 136x HG008-T Pacbio Onso, 77x HG008-N-D Element, 111x HG008-T Element, 118x HG008-N-D Illumina, 161x HG008-T Illumina, 20x HG008-N-P ONT 25Kb, 54x HG008-T ONT-UL, HG008-N Haplotype 1 assembly, HG008-N Haplotype 2 assembly, HG008-T Haplotype 1 assembly, HG008-T Haplotype 2 assembly, a BED file denoting repeats in GRCh38, and a BED file denoting Segmental Duplications. The alignments were hosted on an NCBI-hosted FTP site for GIAB data or an S3 bucket.^14^ Curation results for the FPs and FNs are in Supplementary Table S11. Based on the curations, somatic variants or potential errors that lifted to multiple GRCh38 locations were excluded from the v0.3 small variant benchmark regions.

### Telomere Length Estimation Methods

We analyzed Nanopore long reads aligned to chromosome ends (*i.e.* first and last 10 kbp) of CMH13v2.0. Those reads were analyzed using Topsicle (https://doi.org/10.1186/s13059-025-03783-4) by quantifying 4-mer repeats based on human telomere pattern (CCCTAA), with TRC cutoff of 0.7, minimum length of long read as 7 kbp and the longest possible telomere length is 30 kbp. The same reads were also processed with Telogator2 (https://github.com/zstephens/telogator2), run with default parameters except for specifying a custom reference containing HG008 subtelomeres via the -t input option.

### Analysis of Ribosomal DNA Regions

Despite their central role in cellular physiology, rDNA loci have historically been excluded from genome-wide analyses because their repetitive structure prevents accurate mapping and assembly using short-read sequencing. This limitation persisted until the CHM13 T2T assembly, which finally resolved the p-arms of the acrocentric chromosomes, yet the rDNA arrays themselves have still not been fully assembled.^5^ As a result, the stability and variation in the rDNA regions have been challenging to characterize, further preventing our understanding of how these arrays change in disease. Long-read sequencing and near-complete genome assemblies as well as new analysis methods now allow for improved interrogation of these regions, enabling simultaneous analysis of rDNA sequence, copy number, and epigenetic state in matched tumor and normal genomes. We examined rDNA copy number, methylation, and the variant profile in the normal duodenal, normal pancreatic and pancreatic tumor tissue from HG008. As all of these measurements vary between individuals to collectively form a unique rDNA fingerprint, it is critical to use matched samples for comparisons.^36^ As the rDNA arrays mostly remain as gaps, we used a k-mer based approach for copy number quantification from short read data,^36^ normalizing estimates to coverage from chromosome 4.

#### rDNA Copy Number Analysis

Copy number of the rRNA gene was estimated using a Snakemake pipeline called CONKORD (Version 8 https://github.com/borcherm/CONKORD). This pipeline identifies bins in a reference genome that are of similar G/C percentage to the provided feature and on the same chromosome. K-mers from the provided feature and matched bins are counted in short-read whole genome sequencing data, then filtered to remove any which occur at counts greater than 3 standard deviations from the mean of the set, as well as those that do not appear at all. K-mers which are not exclusive to the matched bins are removed from the analysis, and those that occur multiple times in the reference have their counts divided by their genomic multiplicity. Copy number is calculated by dividing the median count of feature k-mers by the median count of matched bin k-mers, which effectively gives the multiple of times that the target feature occurs relative to the average genomic background from a similar sequence context. The bin size used was 2 kb, and k-mers were of size 31. The 31-mers were extracted from the 18S portion of rDNA reference sequence KY962518.1 and counted in HG008 tumor and normal samples. Chromosome 4 of human reference genome chm13 v2.0 was used for background k-mer normalization. This chromosome was chosen as it was consistently haploid in the tumor sample, allowing for a stable expected k-mer count at half the value of the typical genomic background. The haploid status of chromosome 4 was confirmed through CONKORD, which showed mean tumor -kmer counts for chromosome 4 at ~28, roughly half of other chromosome k-mer counts at ~52. Normal pancreas and duodenum counts were consistent across all chromosomes. K-mer spectra further validated this, with a distinct haploid peak using chromosome 4 k-mers in the tumor sample, leading to division of the CONKORD output by 2 for just the tumor sample.

#### DJ Copy Number Analysis

A sequence known as the distal junction (DJ) is present on each acrocentric chromosome,^83^ distal to the rDNA array; a normal diploid genome contains 10 copies, with variation ranging from 9 to 11 copies.^35^ The Illumina short read sequencing data for the HG008 normal duodenum, normal pancreatic, and tumor pancreatic samples was used for estimating DJ copy number with a reference free k-mer based approach in the DJCounted framework (https://github.com/marbl/DJCounter). A curated set of DJ specific 31-mers, present once per distal junction and shared across the five acrocentric chromosomes in CHM13 v2.0 reference assembly, was used to quantify DJ abundance. K-mer counts were generated from sequencing reads and normalized to the diploid coverage peak derived from genome wide k-mer frequency distributions. This approach enables robust quantification of DJ copy number independent of read alignment and is described in detail recently.^35^

#### rDNA methylation Analysis

For these samples, we cannot assign reads to specific chromosomes, and therefore collapsed all the reads in each sample into a single repeat unit to examine the average methylation level. Oxford Nanopore Technologies (ONT) whole-genome sequencing data for the HG008 normal duodenum, normal pancreatic, and tumor pancreatic samples was used for rDNA methylation analysis. Raw BAM files containing per-read modification tags were converted to FASTQ format using samtools (v1.23.1) with retention of base modification tags (samtools fastq -T “*”) to preserve methylation information. Reads were aligned to the human 45S rDNA reference sequence (NCBI accession KY962518.1) using minimap (v2.26) with ONT specific parameters (-x map-ont). Alignments were sorted and indexed using samtools. To restrict analyses to primary alignments, supplementary and secondary reads were removed (-F 0x900). CpG methylation frequencies were then quantified using modkit (v0.6.0) with the pileup function, specifying CpG context and combining strands. Resulting per-site methylation calls were processed in R, where sites with coverage <100 reads were excluded. Methylation levels were subsequently aggregated into 200 bp bins across the rDNA reference, and mean methylation per bin was calculated as the ratio of modified to total reads.

To investigate the distribution of methylated reads, reads were then aligned to human rDNA reference sequence KY962518.1 using minimap2 (v 2.26), and were filtered to keep only alignments covering greater than 70% of the reference sequence at greater than 90% identity. These alignments were separated into individual bam files, which split very long reads that spanned multiple rDNA units, and provided one file per reference-spanning segment. Filtering and separation of reads was performed using samtools (v 1.23.1), bedtools (v2.30.0), and python scripts (v 3.9.12). The tool modkit (v 0.2.0) was used to translate methyl tags into a bedmethyl format, using the filtering parameter to remove the 10% least-confident methylation calls. Average methylation was compiled per-read over the gene body, intergenic spacer, and promoter regions.

#### rDNA Variant Calling

The Illumina short read sequencing data for the HG008 normal duodenum, normal pancreatic, and tumor pancreatic samples was used for single nucleotide variant calling. The data was trimmed using Trim Galore (v0.6.10) to remove adaptors and based with a Phred score less than 20. The trimmed reads were aligned to KY962518.1 NCBI rDNA reference using BWA MEM (v0.7.17). The aligned SAM files were converted to BAM format, sorted, and indexed using samtools (v1.23.1). Variant calling was performed for each sample using LoFreq (v2.1.5).^84^ The resulting VCF files were filtered to remove variants with a quality score less than 30. Downstream analysis excluded variants with less than 5% allele frequency.

### Generative AI Use

Analyses for and editing of this manuscript were assisted by Claude Code (Sonnet 4.6 and Opus 4.6), and Gemini 3.1 Pro. All content, scientific claims, and conclusions have been reviewed and verified by the authors to ensure accuracy and originality.

## Supplementary Material

Supplement 1

Supplement 2

## Data Availability

All sequencing data presented are publicly accessible in NCBI BioProject (PRJNA200694)^17^. HG008 benchmark data, related alignments, and assembled genomes are also accessible from an NCBI-hosted GIAB FTP site (https://ftp.ncbi.nlm.nih.gov/ReferenceSamples/giab/data_somatic/HG008/). Curations of SVs are in https://github.com/jzook/HG008SVcuration/issues. Draft v0.5 somatic SV and CNV benchmark is at https://ftp-trace.ncbi.nlm.nih.gov/ReferenceSamples/giab/data_somatic/HG008/Liss_lab/analysis/NIST_HG008-T_somatic-stvar-CNV_DraftBenchmark_V0.5-20260318. Draft v0.3 somatic small variant benchmark is at https://ftp-trace.ncbi.nlm.nih.gov/ReferenceSamples/giab/data_somatic/HG008/Liss_lab/analysis/NIST_HG008-T_somatic-smvar_DraftBenchmark_V0.3-20260425. HG008-N v6.3 assembly is at https://nist-giab.s3.us-east-1.amazonaws.com/giab_tumor-normal/analysis/HG008/NIST_asm_dev/HG008N_v6.3/HG008N_v6.3.fasta.gz HG008-T v3.2 tumor assembly is at https://nist-giab.s3.us-east-1.amazonaws.com/giab_tumor-normal/analysis/HG008/NIST_asm_dev/HG008T_v3.2/HG008T_v3.2.fasta.gz.
