# Supplementary material for: A complete human pancreatic cancer genome
Source: bioRxiv. 2026 May 10:2026.05.01.722316. Preprint. [Version 2] doi: 10.64898/2026.05.01.722316 (PMC13174521; doi:10.64898/2026.05.01.722316)
Supplement: Supplement 1 [file media-1.pdf]

A complete human pancreatic cancer genome Supplemental Figures

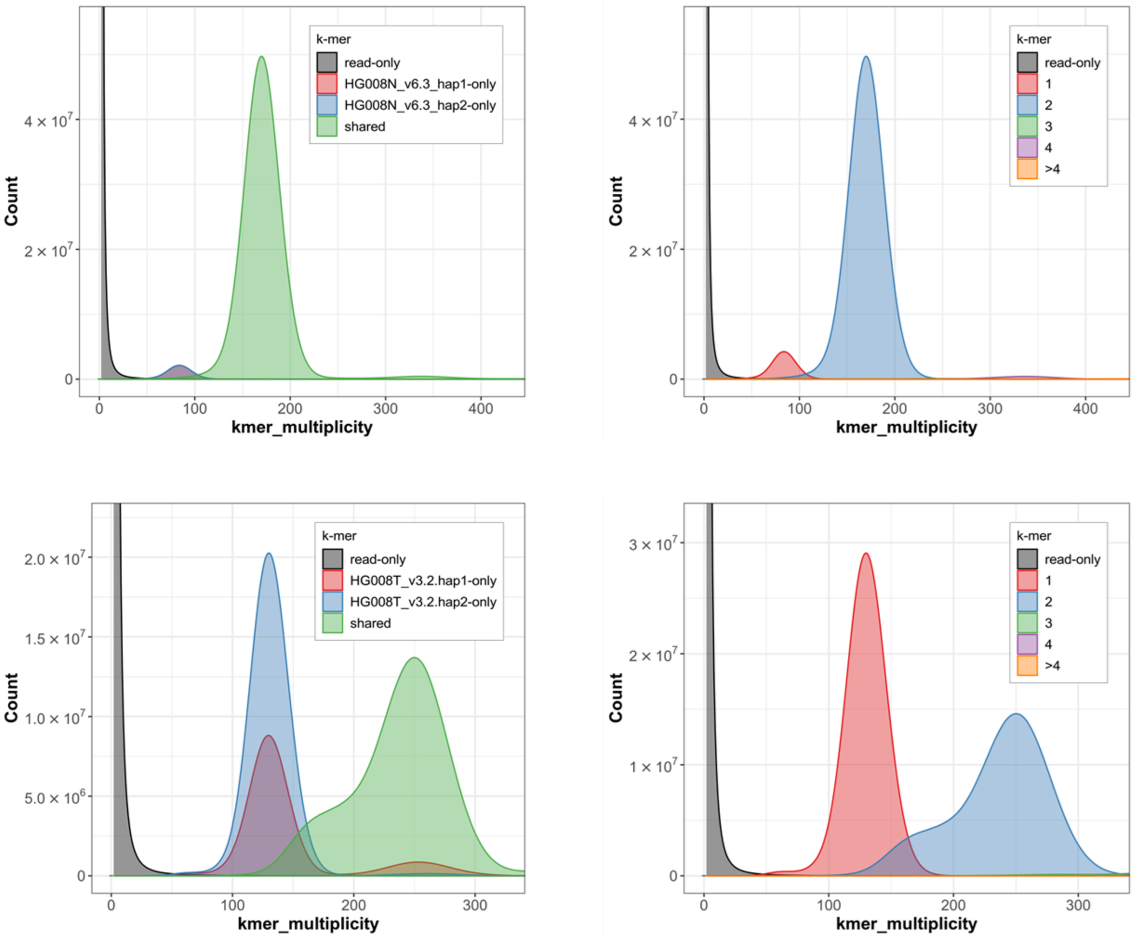

Supplementary Fig. 1. Kmer-based assembly quality evaluation (QV=70 for HG008-N, and QV=72 for HG008-T) using Merqury[85] (k=31 with hybrid database from PacBio HiFi and Illumina reads).

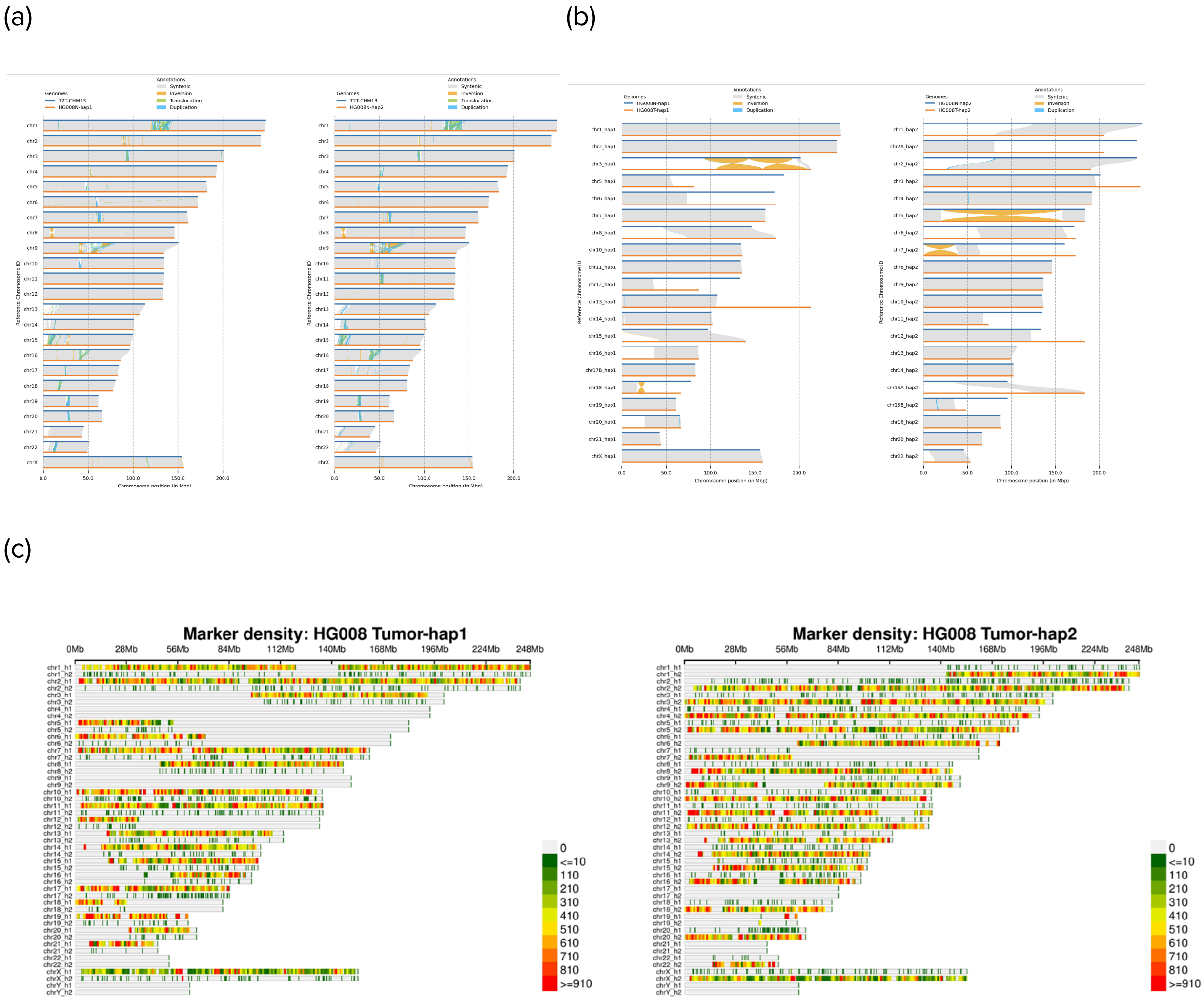

Supplementary Fig. 2: Tumor and normal assembly evaluations. (a) Assembly alignments of HG008N_hap1 and HG008N_hap2 to T2T-CHM13 reveal both structural similarities and dissimilarities by SyRI plot; (b) Alignments of matched tumor haplotype with normal haplotype assemblies shows large structural variations by SyRI; and (c) Distribution of haplotype-specific markers on each HG008T haplotype.

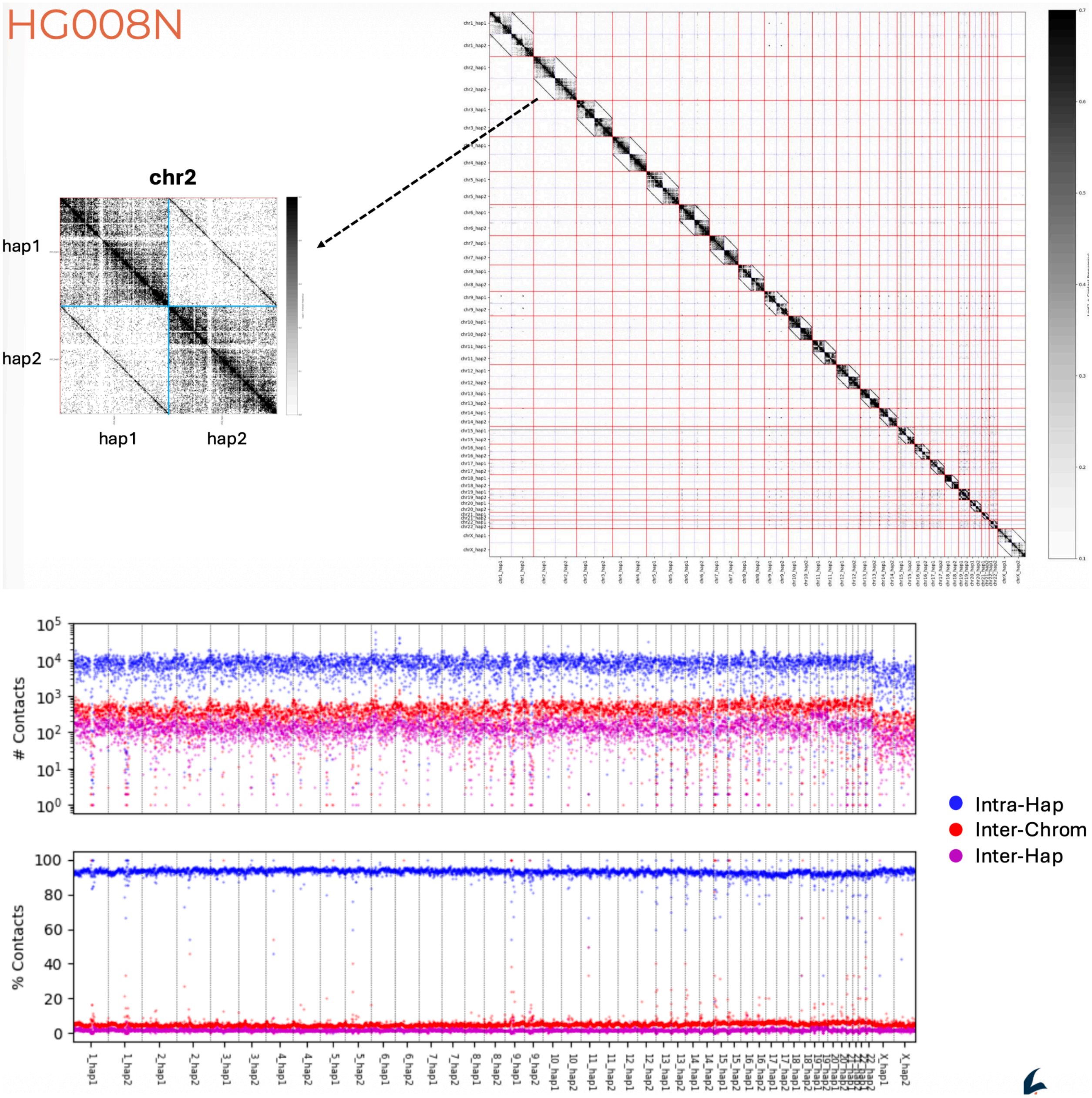

Supplementary Fig. 3: Phased mapping of Dovetail Hi-C to diploid normal assembly confirms structural and phasing accuracy.

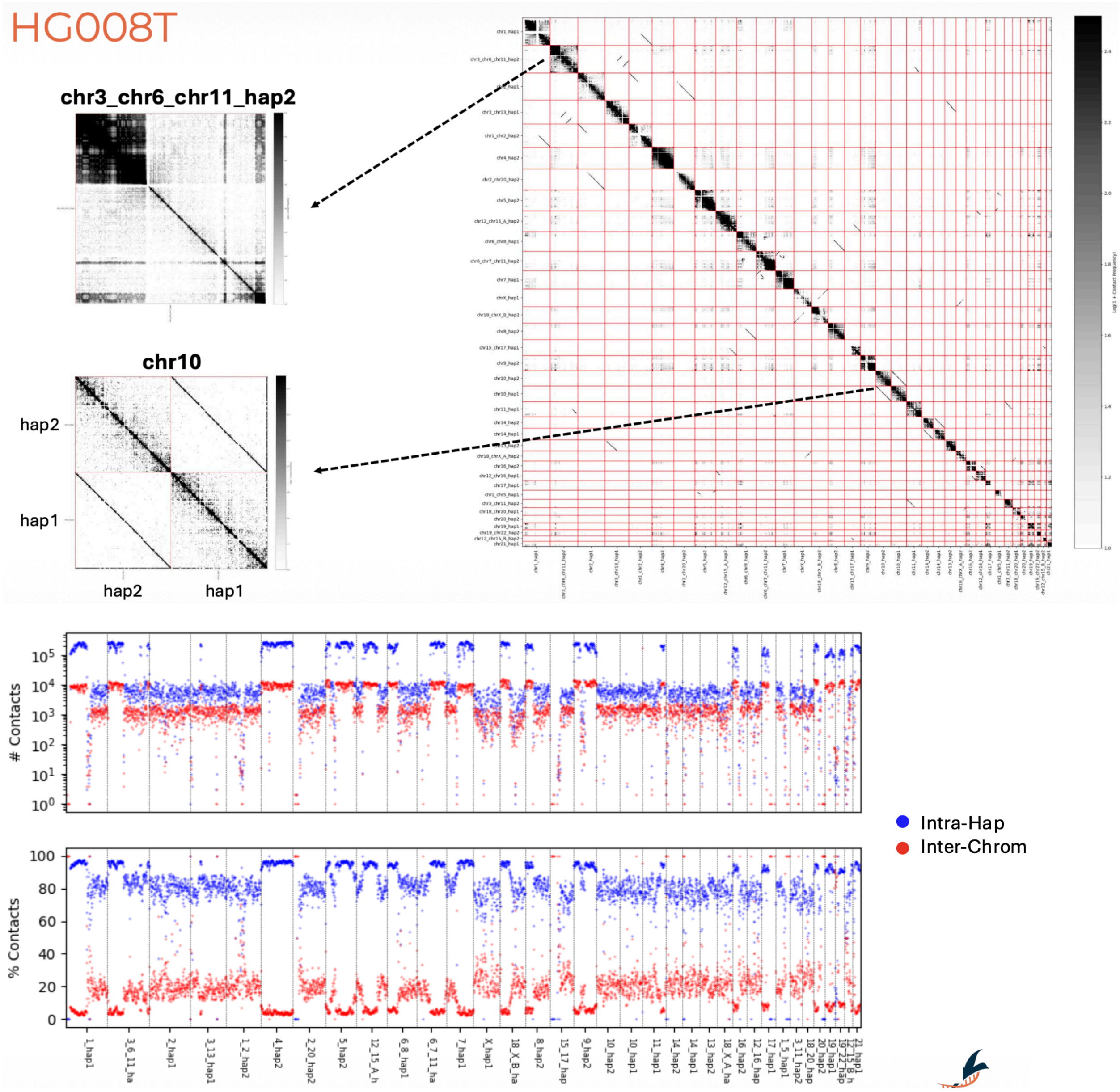

Supplementary Fig. 4: Phased mapping of Dovetail Hi-C to diploid tumor assembly confirms structural and phasing accuracy.

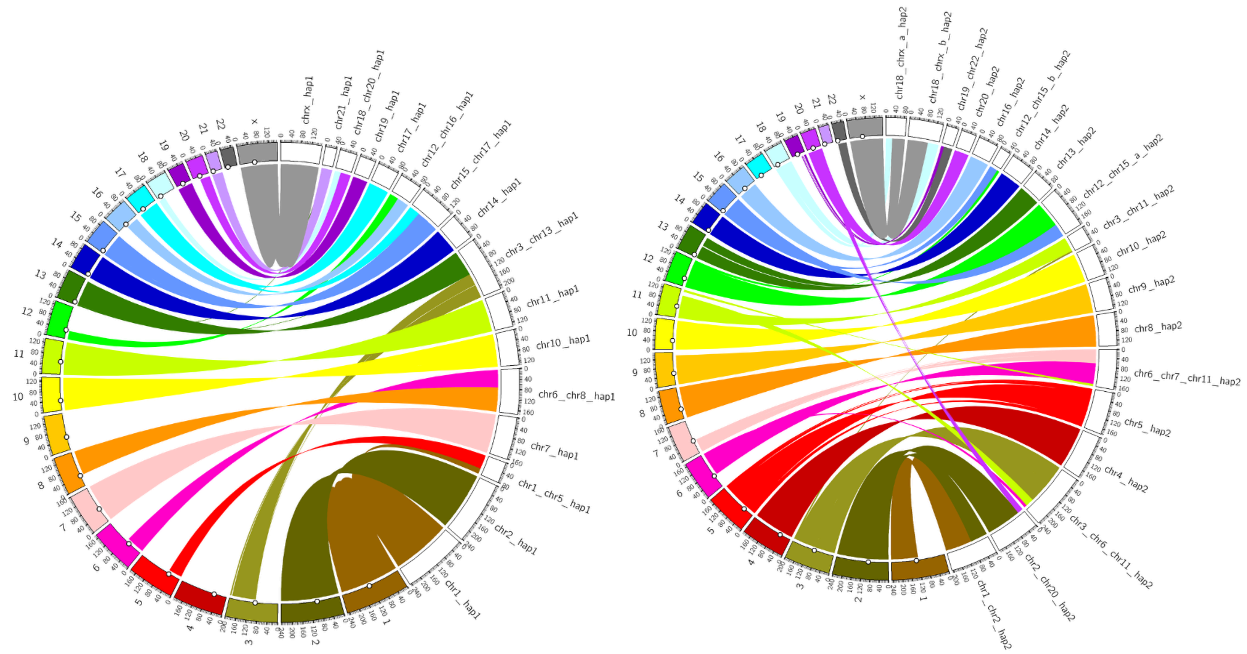

Supplementary Fig. 5: Alignments of tumor and normal assembly haplotypes (right and left sides of each circos plot, respectively) reveal large chromosomal-scale deletions, duplications, and translocations. Large chromosome-scale CNVs >10Mbp were caused by loss of whole chromosomes (chr4_hap1, chr9_hap1, chr17_hap2, chr21_hap2, and chr22_hap1) or by unbalanced translocations between chromosomes (e.g. chr3p, chr5q, chr6q, chr8p, chr12q, chr16p, and chr18q in hap1, and chr1p, chr6p and chr7q in hap2).

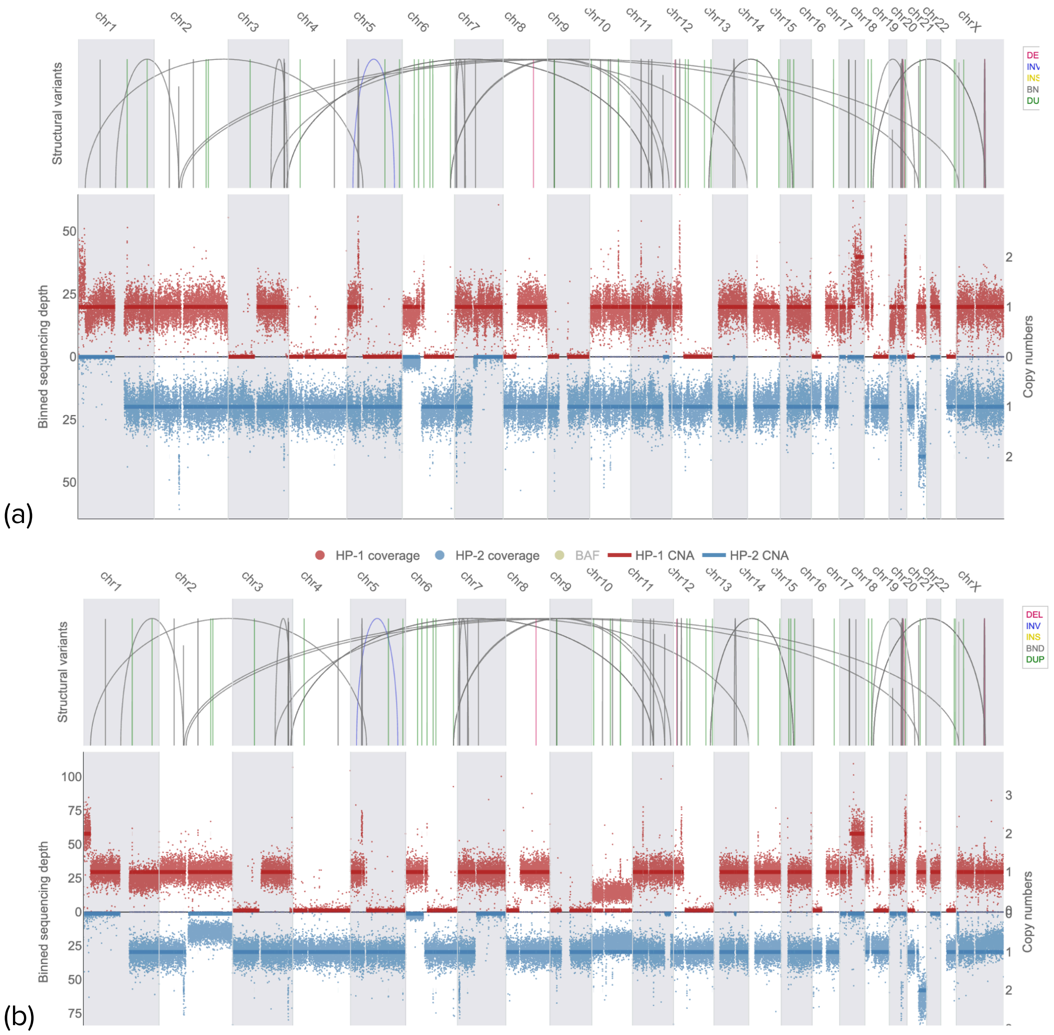

Supplementary Fig. 6: Haplotype-resolved copy number from long reads demonstrates many large deletions and fewer large duplications and copy neutral loss of heterozygosity. (a) Copy number from NIST passage 21 HiFi data, which contains no large high frequency subclonal CNVs and closely matches the assembly. (b) Copy number from MGH passage 23 ONT data, which contains some large high frequency subclonal CNVs (e.g., 1q, 2q, 10, X) that are not contained in the assembly because they are not truncal. While most truncal chromosome-scale CNVs were deletions, a large duplication on chr20_hap2 resulted in 3 total copies. Also, the only remaining haplotype of chr1p_hap1 and chr17q_hap1 had large partial duplications caused by unbalanced translocations that result in CNLOH. Smaller copy number changes were caused by tandem duplications, large deletions, or loss or gain of sequences between translocation breakpoints. Chr19 has the largest variation in copy number due to nested foldback inversions. Phasing copy number changes enables us to see that chr6_hap2 lost most of the p arm in most cells due to a translocation with chr8_hap2 and chr6_hap1 lost most of the q arm due to a translocation with chr7_hap1 in the centromeres.

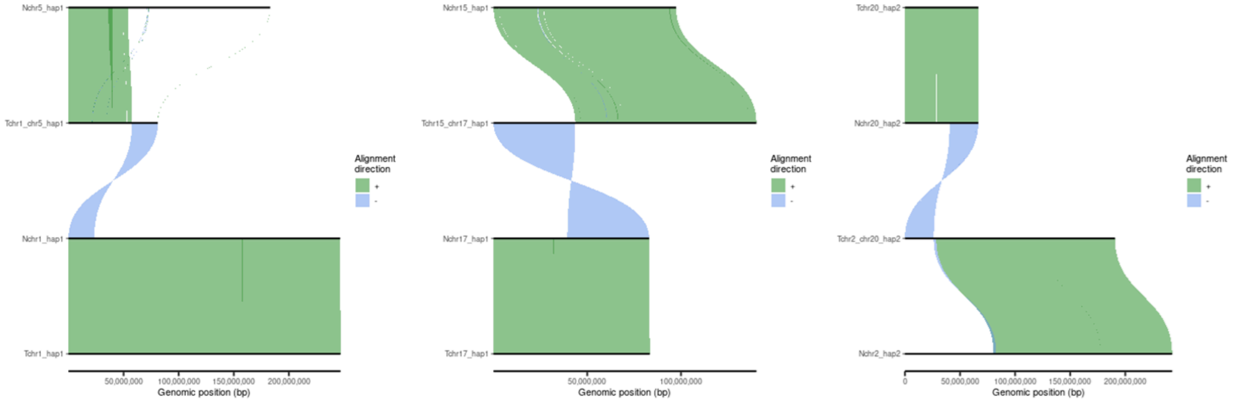

Supplementary Fig. 7: While most large somatic CNVs were deletions, large duplications caused by translocation on chr1_hap1 and chr17_hap1 caused CNLOH, and a duplication on chr20_hap2 resulted in 3 total copies.

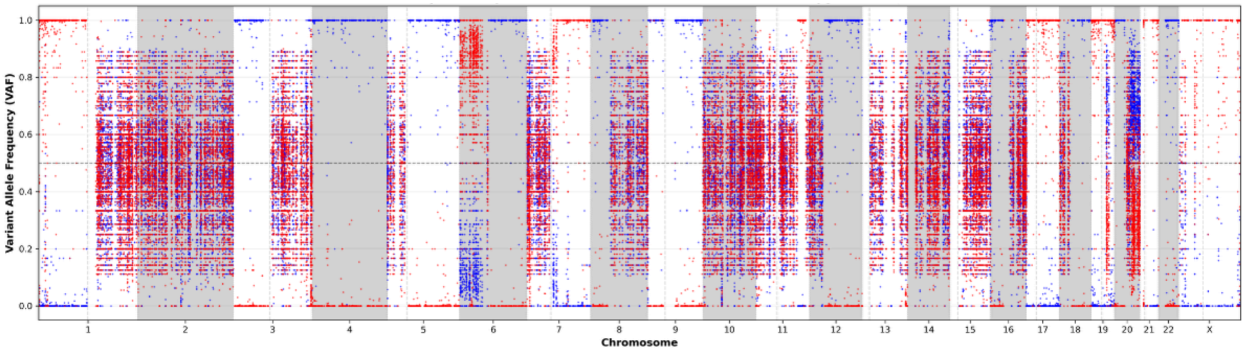

Supplementary Fig. 8: VAF of heterozygous SNVs in RNA sequencing shows allele-specific expression of haplotype 1 (red) and haplotype 2 (blue). RNA sequencing was performed on NIST passage 21 cells, and phase information for the SNVs was inferred from genomic data. The complete LOF on chromosome X demonstrates that the tumor cells retained silencing despite a balanced chr18_hap2/chrX_hap2 translocation involving the silenced copy of chrX. Because XIST is on the portion of chrX attached to chr18q, we initially hypothesized that chr18q haplotype 2 might also be silenced; however, silencing did not spread to most of the autosome, as chr18q retains expression of many haplotype 2 genes. The absence of haplotype 1 expression on chr18q is explained by a separate event: a complex unbalanced translocation between chr18 and chr20, together with a large inversion on haplotype 1, deletes chr18q on haplotype 1. Interestingly, the chr18/chr20 breakpoint lies only ~30 kbp from the chr18/chrX translocation breakpoint on haplotype 2 (https://github.com/jzook/HG008SVcuration/issues/272). In addition, chrXp haplotype 2 expression remains repressed even though XIST is no longer on this hybrid chromosome, consistent with maintenance of the established epigenetic state. Overall, the VAF from RNA-seq is consistent with the VAF from genome sequencing.

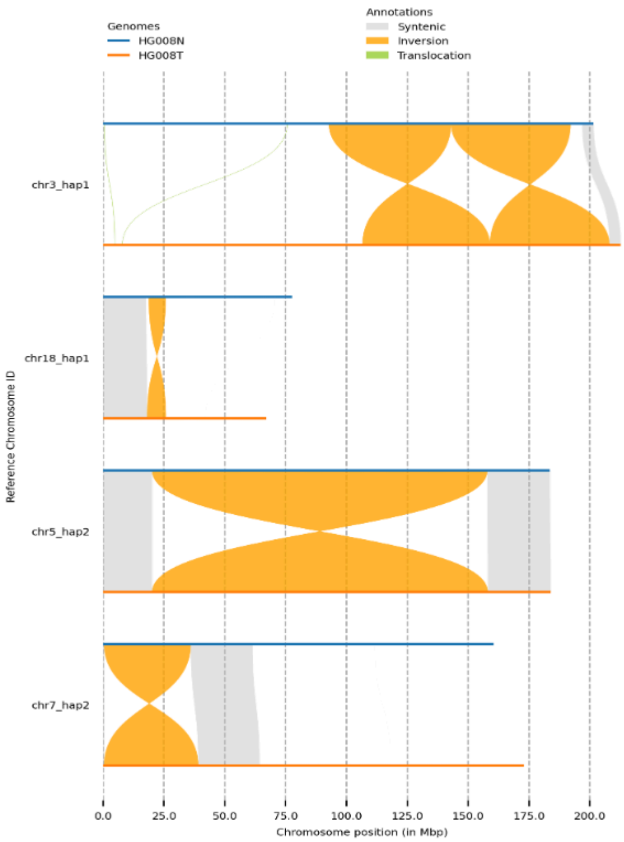

Supplementary Fig. 9: HG008 contains 3 reciprocal inversions, nine non-reciprocal foldback inversions, five non-reciprocal inversions that do not cause duplications, and one inversion related to a tandem duplication. The reciprocal inversions are 2.7 kbp (chr3_hap2), 36 Mbp (chr7_hap2), and 138 Mbp (chr5_hap2) in size, and all result in loss of 1 bp to 363 bp of sequence at each breakpoint. 9 foldback inversions cause 537 bp to 2.32 Mbp of duplicated sequence near interchromosomal translocation breakpoints, including 4 nested events on chr19 discussed below. Also, two occur in chr1 and chr8 centromeric active HORs and one in acrocentric short arm repeats near the beginning of chr15. 5 inversions associated with translocations do not cause duplications, with 2 smaller than 1 kbp and others 8, 54, and 99 Mbp. The 8 Mbp inversion at chr18_hap1 translocation with chr20_hap1 can only be seen precisely in the normal assembly because one breakpoint of the inversion is in the chr18 centromeric active HOR. The chr3-chr13 translocation has complex 54 and 99 Mbp inversions on chr3_hap1, also with one breakpoint in centromeric HSat1A. While not a translocation-associated inversion, there is also a 4,577 bp inverted duplication of sequence starting approximately 3 kbp past the end of a 365 kbp tandem duplication on chr17 that is inserted between the two copies of the tandem duplication (https://github.com/jzook/HG008SVcuration/issues/266). The only non-complex translocations are the chr1_hap1/chr5_hap1 and chr12_hap1/chr16_hap1 unbalanced translocations as well as chr12_hap2/chr15_hap2 (which disrupts the KDM2B gene) and chr18_hap2/chrX_hap2 balanced translocations (Fig. 1b).

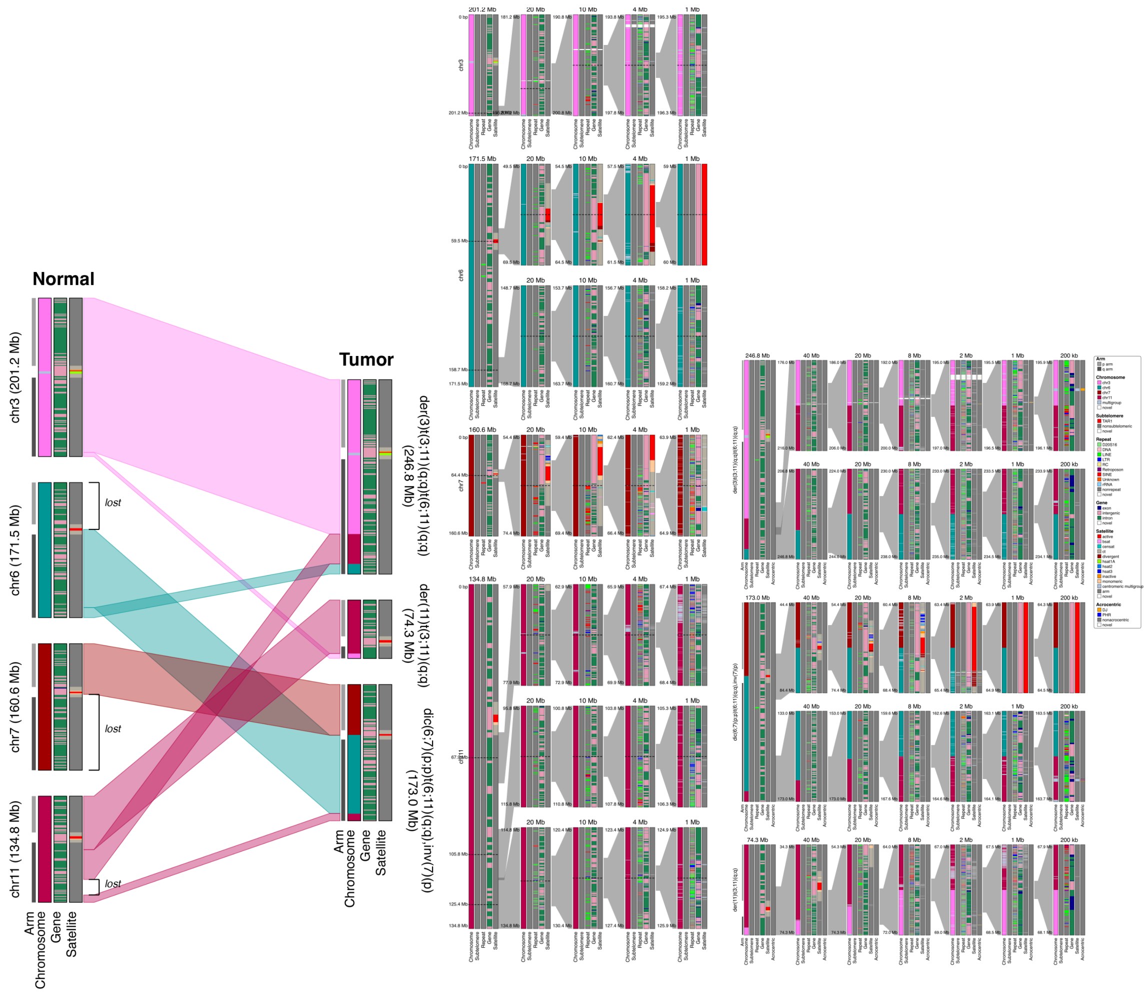

Supplementary Fig. 10: Chromoplexy causing a complex series of events linking chromosomes 3, 6, 7, and 11 in three hybrid tumor chromosomes. Karyoscope's chromosome, subtelomere, repeat, gene, satellite annotations are shown across the entire chromosomes and at different resolutions around each interchromosomal breakpoint. Note that inversions, including the one on chr7p, are not shown by karyoscope.

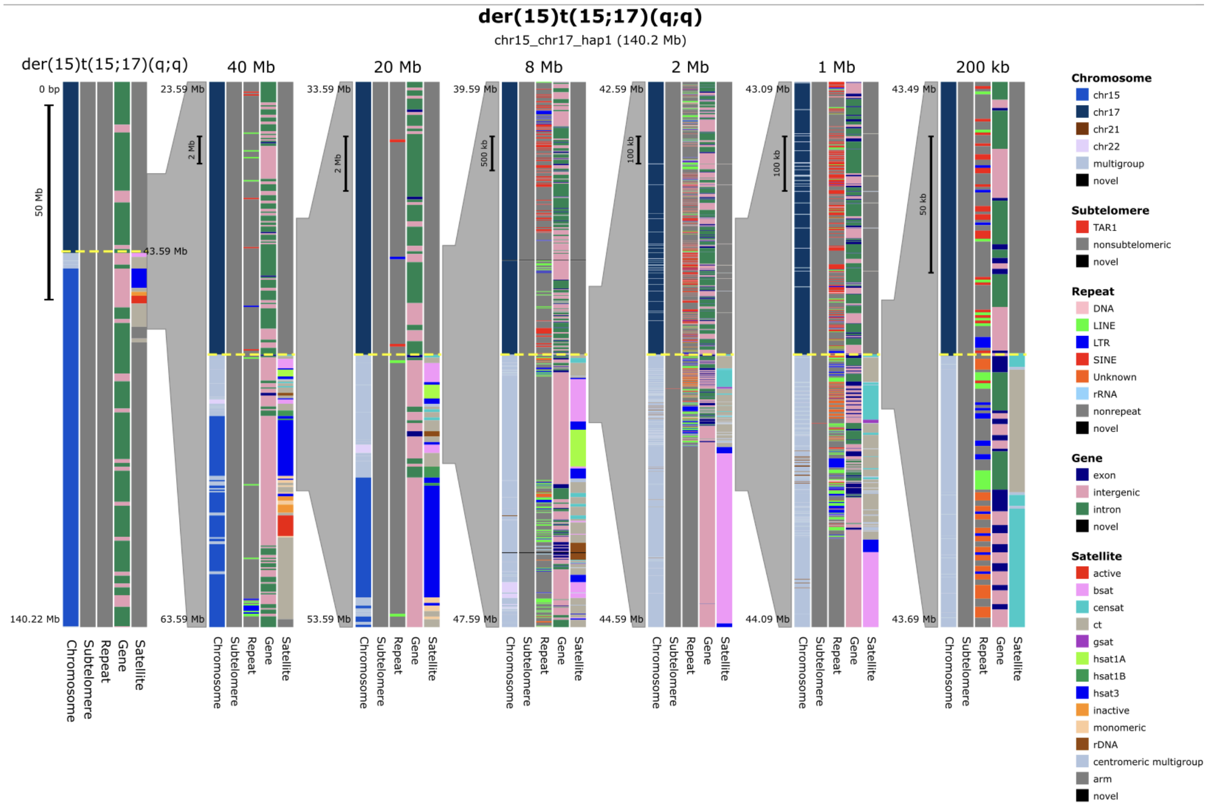

Supplementary Fig. 11: der(15)t(15;17)(q;q) example of acrocentric breakpoints not resolvable without a normal assembly. This event is caused by a translocation occurring in an acrocentric short arm near the p arm telomere of chr15_hap1, which is attached to a duplication of the only remaining copy of chr17. There is an inverted duplication of 25 kbp of chr15 starting 40 kbp from the telomere. Interestingly 210 bp from the middle of chr11 is duplicated in between the copies of the inverted duplication. This translocation is only clear using matched normal and tumor assemblies because acrocentric short arms frequently exchange material between chromosomes. Furthermore, the HG008 normal short arms of chr15 haplotype 1 and chr14 haplotype 2 are nearly identical in the first 4.1 Mbps from the telomere through the rDNA arrays, with only one SNV and one indel difference in this region outside the rDNA arrays. This region includes the rDNA arrays, though these arrays were not fully resolved in the assemblies. The tumor assembly contains 11 somatic SNVs on chr15 and 12 somatic SNVs on chr14 in this region supported by HiFi reads, illustrating the unusual situation of far fewer germline SNVs than somatic SNVs in this region. Karyoscope's chromosome, subtelomere, repeat, gene, satellite annotations are shown across the entire chromosomes and at different resolutions around each interchromosomal breakpoint. Note that inversions are not shown by karyoscope.

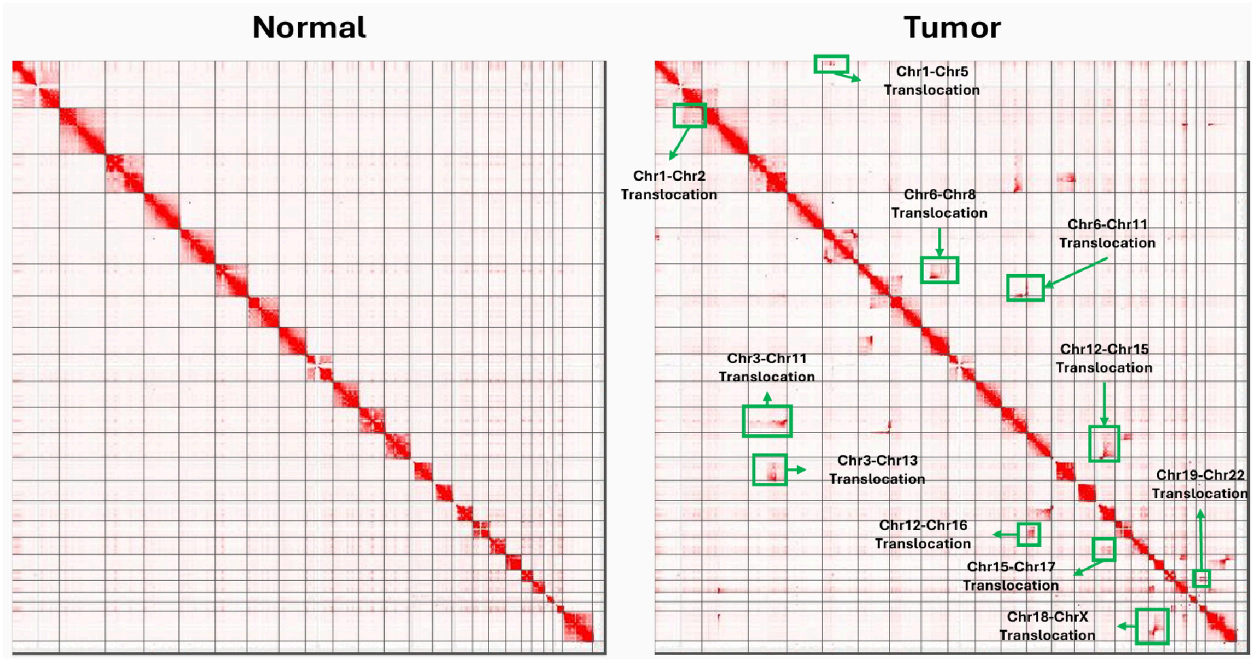

Supplementary Fig. 12: Hi-C Contact maps for Tumor and Normal on GRCh38. We highlight the impact of a few large interchromosomal translocations and inversions evident when comparing the Hi-C contact map aligned onto the GRCh38 reference assembly of the tumor sample against its matched normal tissue sample.

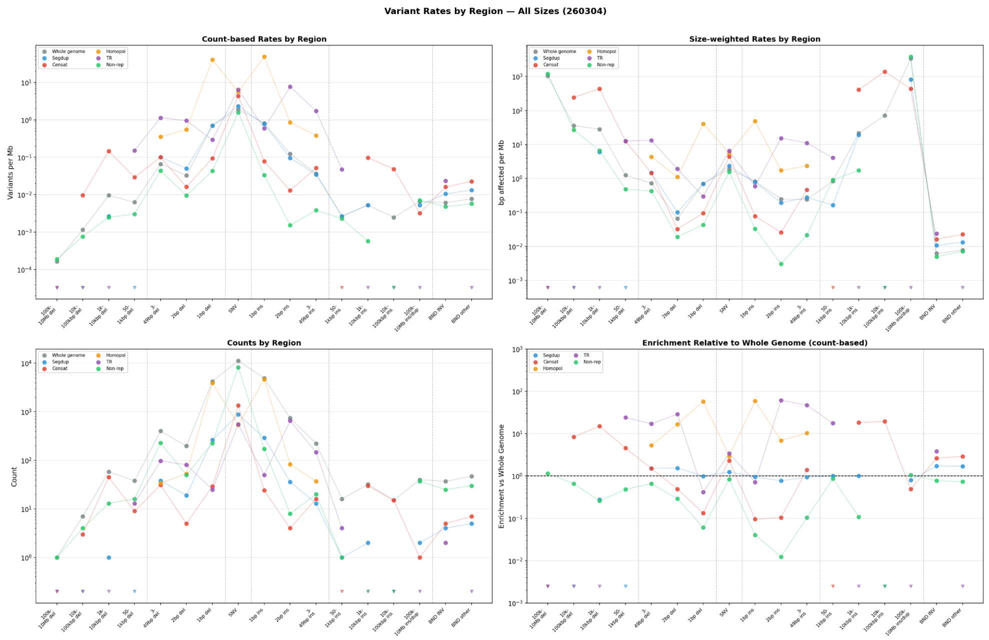

Supplementary Fig. 13: Small variant and SVs are generally enriched in repetitive regions. There are more 1-2 bp insertions than deletions, but more 3 bp to 10 kbp deletions than insertions, and more insertions than deletions larger than 10 kbp. Comparing repeat unit size, repeat length, and variant type we observe the following trends: 1 bp and 2 bp indels predominate in homopolymers and dinucleotide TRs; larger insertions and deletions predominate in STRs, VNTRs, and satellites; 10 kbp to 100 kbp insertions/duplication predominate in satellites; and tandem duplications > 100 kbp predominate in non-repetitive regions. Large homopolymers exhibit interesting characteristics including: 14 1bp indels in >50bp homopolymers, 7 contractions of 10-30bp in homopolymers longer than 50bp in our benchmark, and 14 deletions >=10bp in homopolymers <50bp. Unlike the 21 contractions of at least 10 bp in homopolymers, which appeared true upon curation, the 5 putative expansions of at least 10bp in homopolymers appeared likely to be assembly errors, mostly haplotype switches in the normal assembly. We observed 607 2bp ins and 80 2bp del in diTRs with 232 SNVs. Similar to previous analysis of mutation rates,[86] there are many more deletions (14) than insertions (2) larger than 9bp. Somatic mutation rates are lower for diTRs than A/T or G/C homopolymers of similar lengths and type, which is opposite what was recently found when merging all lengths and types for a smaller number of germline de novo mutations with similar accurate short reads.[18] However, it is similar to a previous analysis of germline variants a large number of number of samples comparing rates at different lengths of homopolymers and diTRs (Extended Data Fig 2 in [87]). Adjacent TRs and homopolymers show distinct patterns. For variants occurring in between two homopolymers each longer than 6bp, there are 65 SNVs, 108 1bp INS, and 84 1bp DEL. Interestingly, within adjacent homopolymers there are 39 5′−TTA−3′ ↔ 5′−TAA−3’ vs 13 5′−TAA−3′ ↔ 5′−TTA−3, similar to spontaneous mutations in *C. elegans*.[88] For adjacent diTRs, SNVs are enriched with 256 SNVs, 67 2bp INS, and 3 2bp DEL.

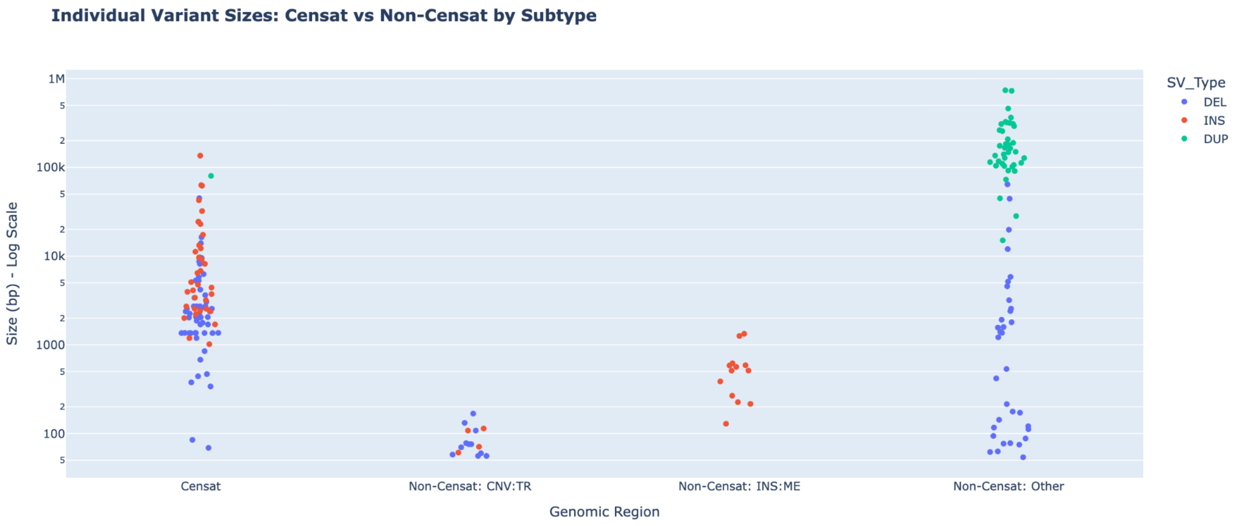

Supplementary Fig. 14: SV size distributions by repeat SV type. While germline variants typically have balanced insertion and deletions with respect to an accurate reference like T2T-CHM13, our tumor/normal assemblies reveal HG008's somatic SVs have distinct deletion and insertion profiles in different types of genomic repeats. In non-repetitive regions, of the 41 SVs smaller than 10 kbp, 27 were deletions and 14 were LINE insertions. Of 40 SVs 10 kbp to 10 Mbp in size, 36 were tandem duplications. STRs and VNTRs have 4 insertions and 13 deletions between 50 and 200 bp, whereas centromere satellite regions have 57 deletions ranging from 69 to 44,973 bp and 46 insertions from 1,018 to 135,740 bp.

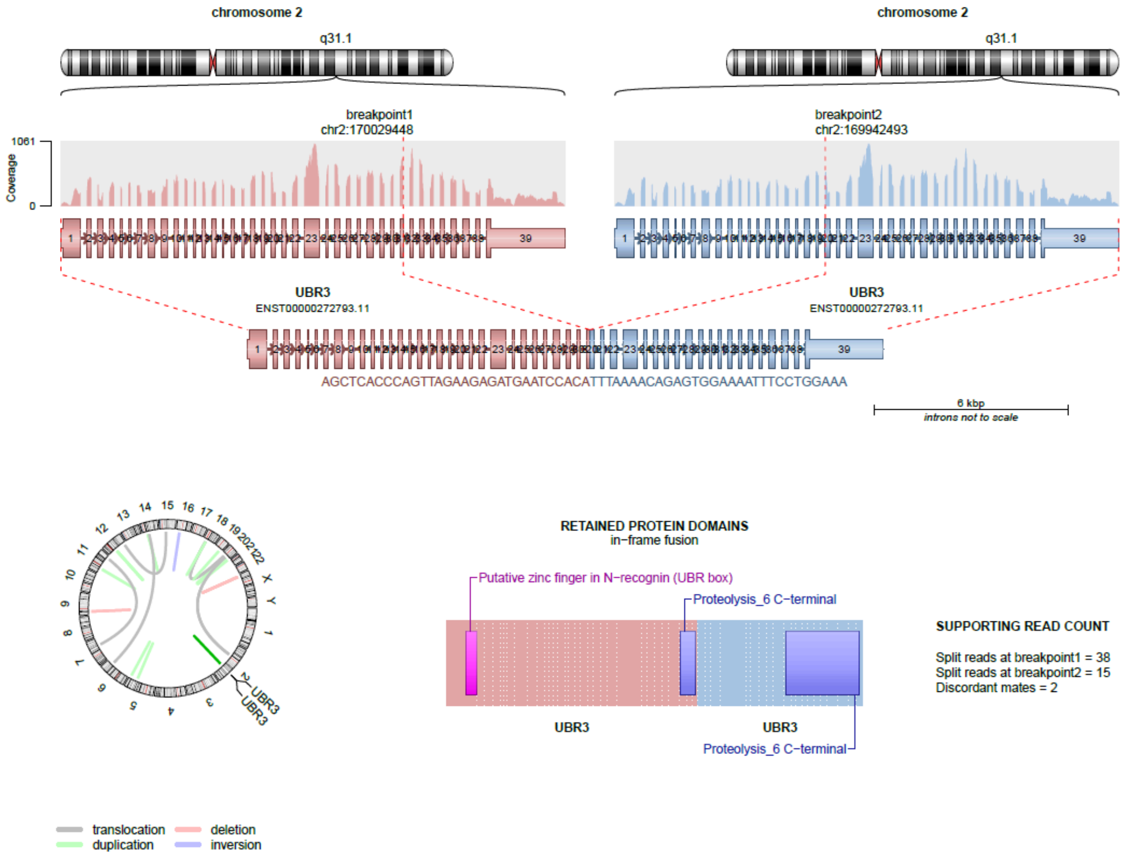

Supplementary Fig. 15: Expression of 92 kbp internal tandem duplication in *UBR3*. Internal tandem duplications in genes include *NFE2L2*, which has ITDs in NSCLC,[89] *UBR3*, which can be important for genome stability,[90], and *BBS9*. Fusion analyses of HG008-T RNA-seq also supports the detection of *UBR3* internal tandem duplication.

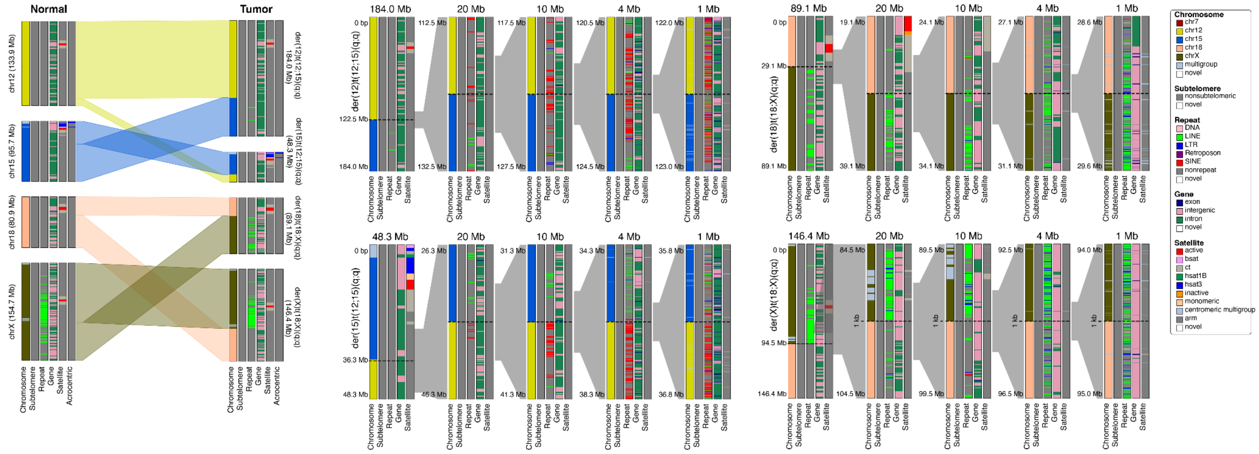

Supplementary Fig. 16: Reciprocal translocations between chr12 and chr15 and chr18 and chrX on haplotype 2. These translocations do not occur in the centromeres and so retain a single copy of a centromere in the resulting tumor chromosomes. Karyoscope's chromosome, subtelomere, repeat, gene, satellite annotations are shown across the entire chromosomes and at different resolutions around each interchromosomal breakpoint. Note that inversions, including the one on chr18, are not shown by karyoscope.

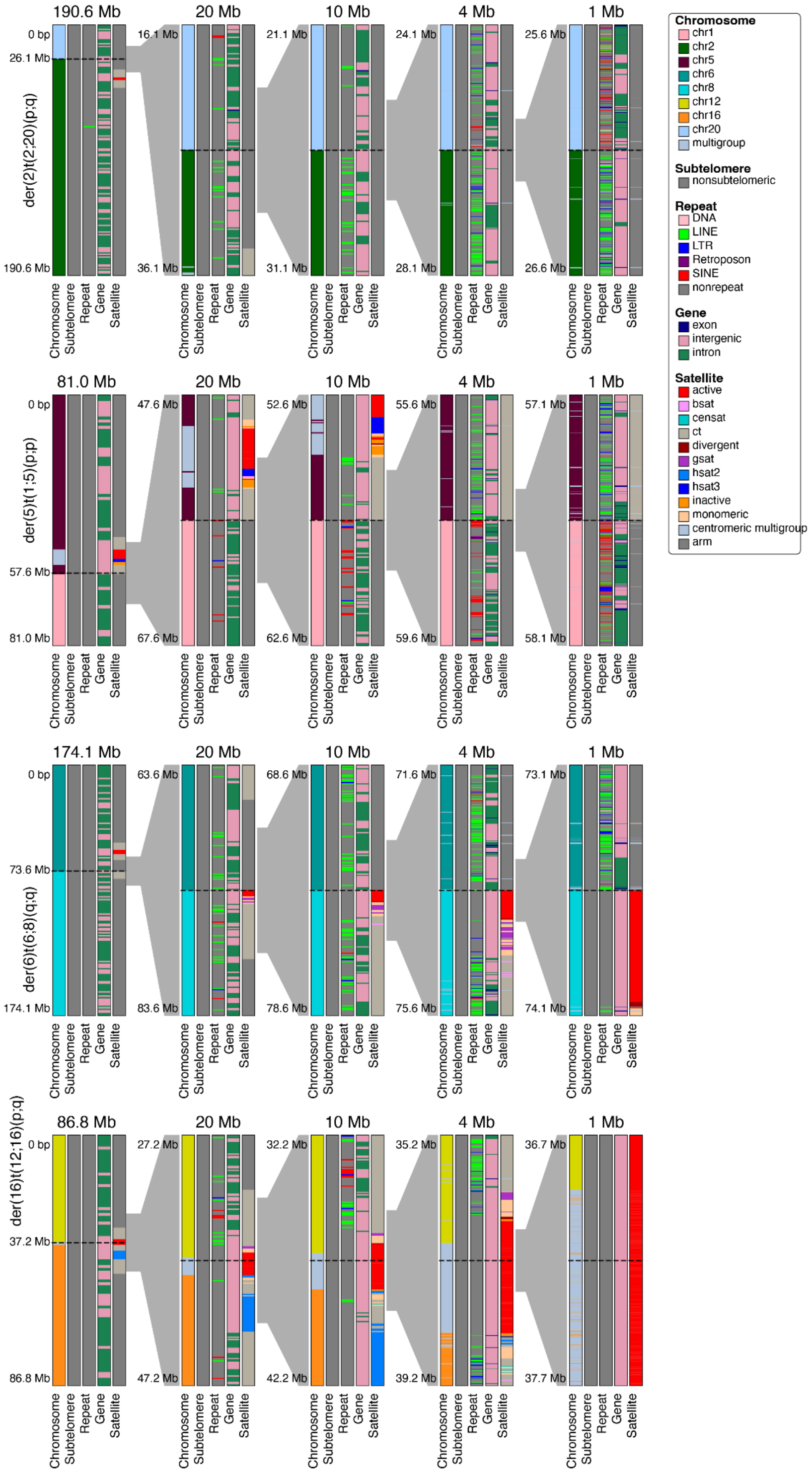

190.6 Mb
20 Mb
10 Mb
4 Mb
1 Mb
der(2)t(2;20)(p;q)
0 bp
26.1 Mb
190.6 Mb
16.1 Mb
36.1 Mb
21.1 Mb
31.1 Mb
24.1 Mb
28.1 Mb
25.6 Mb
26.6 Mb
81.0 Mb
der(5)t(1;5)(p;p)
57.6 Mb
81.0 Mb
47.6 Mb
67.6 Mb
52.6 Mb
62.6 Mb
55.6 Mb
59.6 Mb
57.1 Mb
58.1 Mb
174.1 Mb
der(6)t(6;8)(q;q)
73.6 Mb
174.1 Mb
63.6 Mb
83.6 Mb
68.6 Mb
78.6 Mb
71.6 Mb
75.6 Mb
73.1 Mb
74.1 Mb
86.8 Mb
der(16)t(12;16)(p;q)
37.2 Mb
86.8 Mb
27.2 Mb
47.2 Mb
32.2 Mb
42.2 Mb
35.2 Mb
39.2 Mb
36.7 Mb
37.7 Mb
Chromosome
Subtelomere
Repeat
Gene
Satellite
Chromosome
chr1
chr2
chr5
chr6
chr8
chr12
chr16
chr20
multigroup
Subtelomere
nonsubtelomeric
Repeat
DNA
LINE
LTR
Retroposon
SINE
nonrepeat
Gene
exon
intergenic
intron
Satellite
active
bsat
censat
ct
divergent
gsat
hsat2
hsat3
inactive
monomeric
centromeric multigroup
arm

Supplementary Fig. 17: Karyoscope's annotations for non-reciprocal translocations. Translocations between chr1 and chr5 and between chr2 and chr20 cause duplications of parts of chr1p and chr20q. These translocations do not occur in the centromeres and retain a single copy of a centromere in the resulting tumor chromosomes originating from the non-duplicated chromosome. Translocations between chr6 and chr8 and between chr12 and chr16 occur in one or both centromeres.

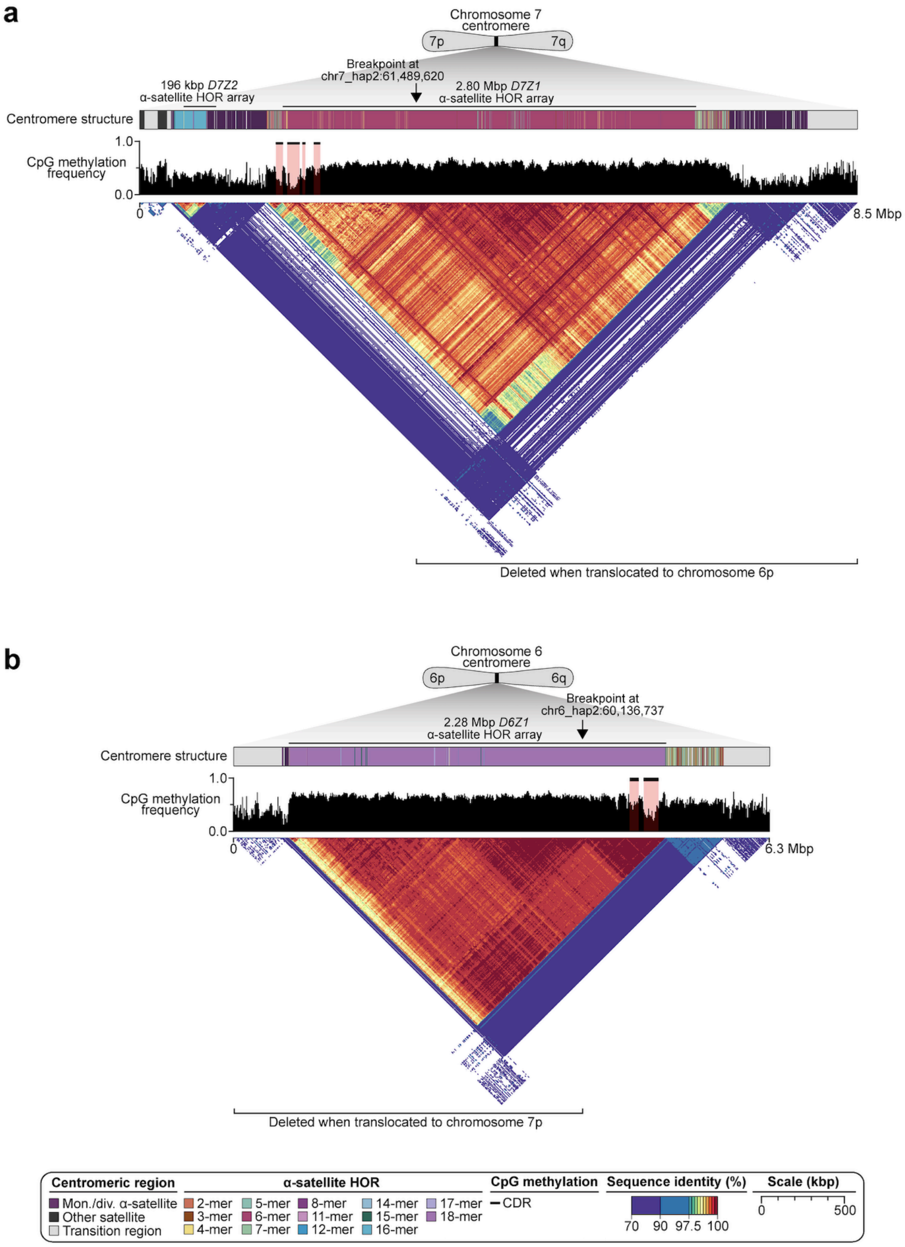

Supplementary Fig. 18. The sequence, structure, and epigenetic landscape of the chromosome 7 and 6 centromeres in normal cells. For both chromosomes a) 7 and b) 6, the CDR sites are retained upon translocation, while most of the α-satellite HOR array is deleted. This creates a putative functional dicentric chromosome with two active centromeres (Fig. 4a).

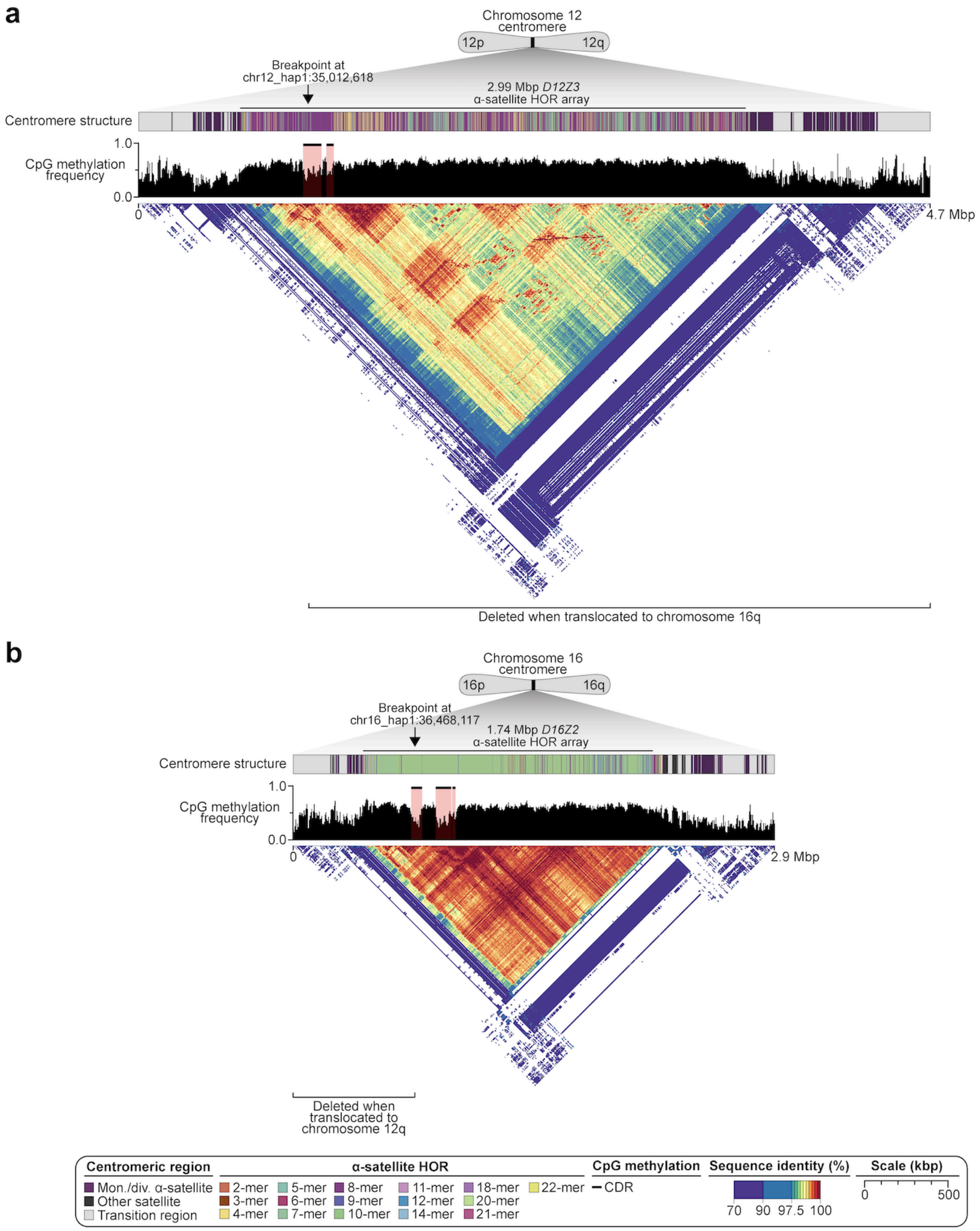

Supplementary Fig. 19. The sequence, structure, and epigenetic landscape of the chromosome 12 and 16 centromeres in normal cells. For both chromosomes a) 12 and b) 16, a portion of the CDR sites is retained upon translocation, while the rest of the CDR site and most of the α-satellite HOR array is deleted. This creates a fused chromosome with a hybrid CDR site residing on two α-satellite HOR arrays (*D12Z3* and *D16Z2*; Fig. 4b).

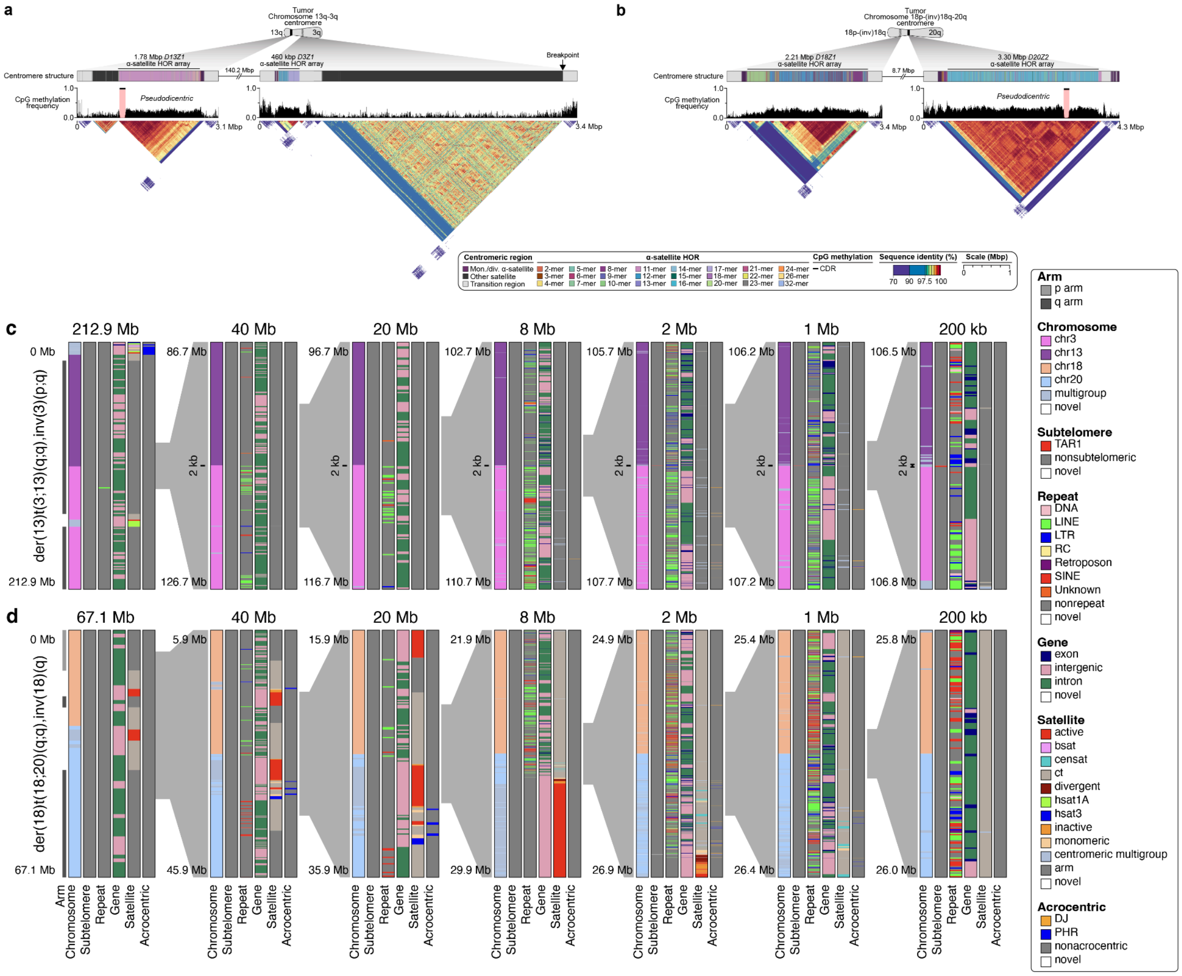

Supplementary Fig. 20. The sequence, structure, and epigenetic landscape of the chromosome 13q-3q and 18p-(inv)18q-20q centromeres in the tumor. a) Upon translocation of chromosome 13q to 3q, the chromosome 3 centromere is inactivated by the complex inversions in Supplementary Fig 9 that delete the CDR, while the chromosome 13 centromere retains the CDR. b) Upon translocation of 18p-(inv)18q to 20q, the chromosome 18 CDR is inactivated by methylation though the inversion in Supplementary Fig 9 also deletes part of the centromere, while the chromosome 20 centromere retains its function. c-d) Karyoscope's chromosome, subtelomere, repeat, gene, satellite annotations are shown across the entire chromosomes and at different resolutions around each interchromosomal breakpoint.

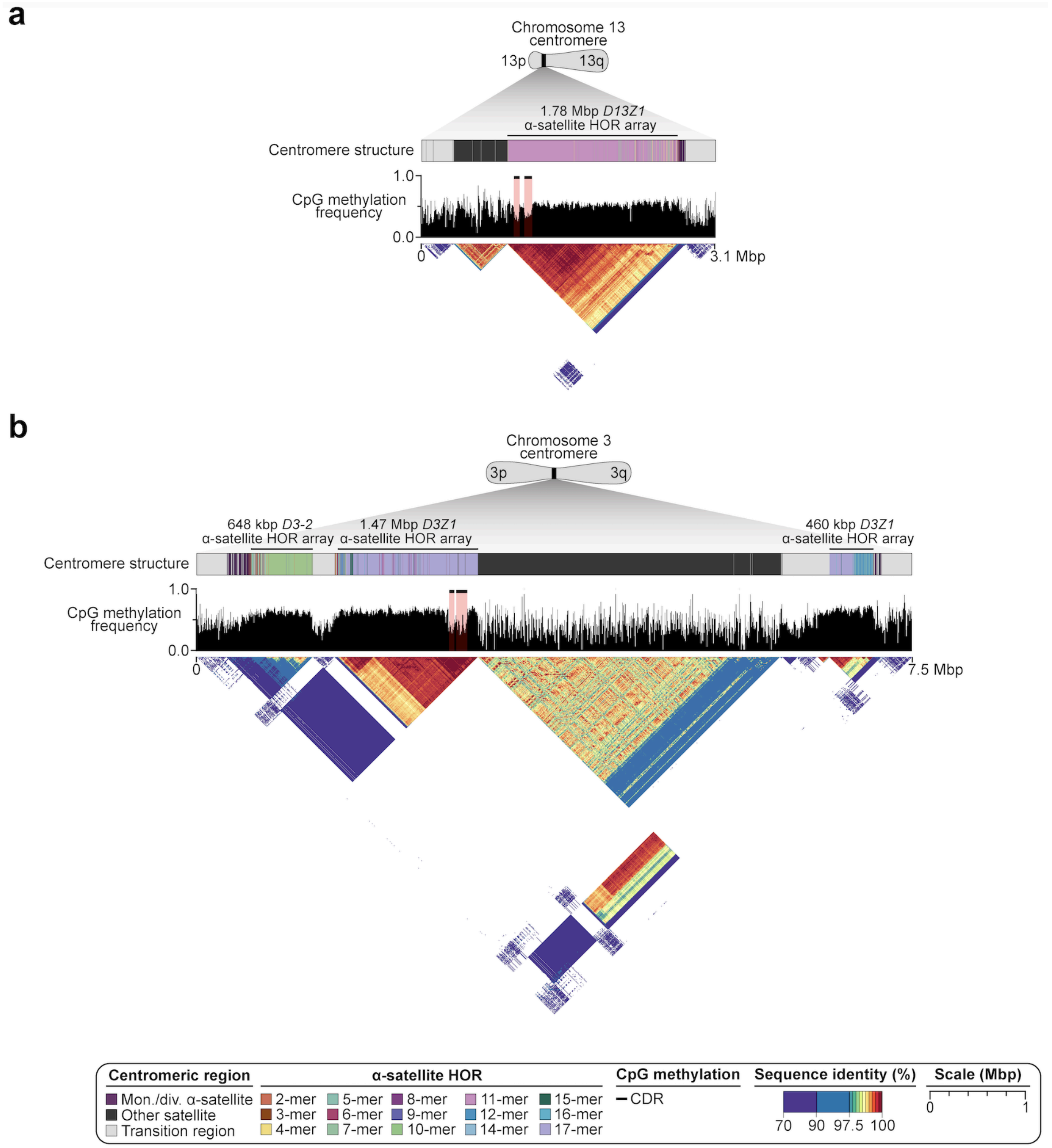

Supplementary Fig. 21. The sequence, structure, and epigenetic landscape of the chromosome 13 and 3 centromeres in normal cells. a) For chromosome 13, the CDR site is retained upon translocation. b) For chromosome 3, however, the CDR-containing centromeric α-satellite HOR array is deleted, and the remaining sequence is inverted upon translocation.

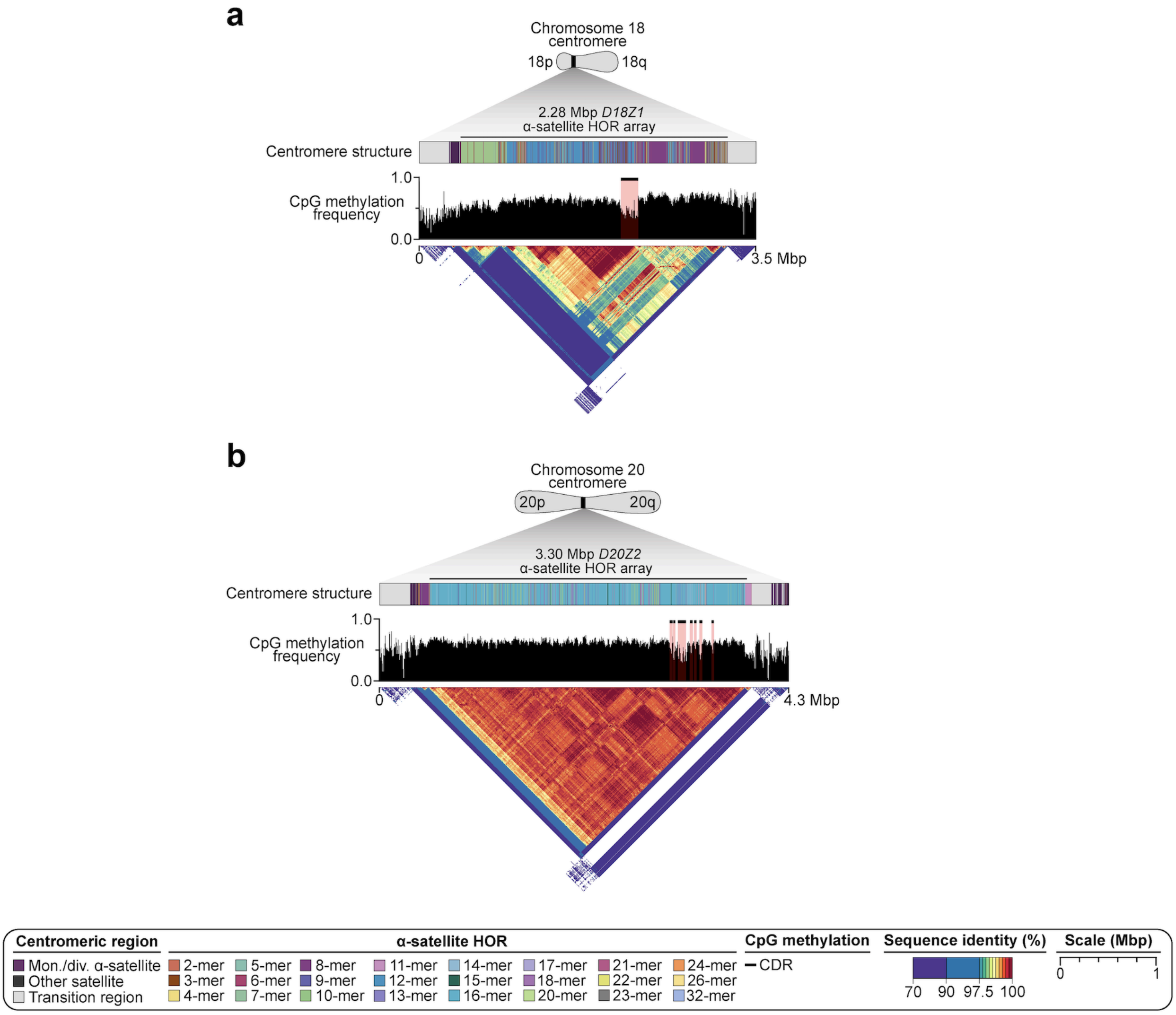

Supplementary Fig. 22. The sequence, structure, and epigenetic landscape of the chromosome 18 and 2- centromeres in normal cells. a) For chromosome 18, the CDR site becomes methylated during tumorigenesis, resulting in inactivation of the centromere, though part of the centromere is also deleted by a large inversion related to the translocation. b) For chromosome 20, however, the CDR position is retained but changes in methylation pattern during tumorigenesis.

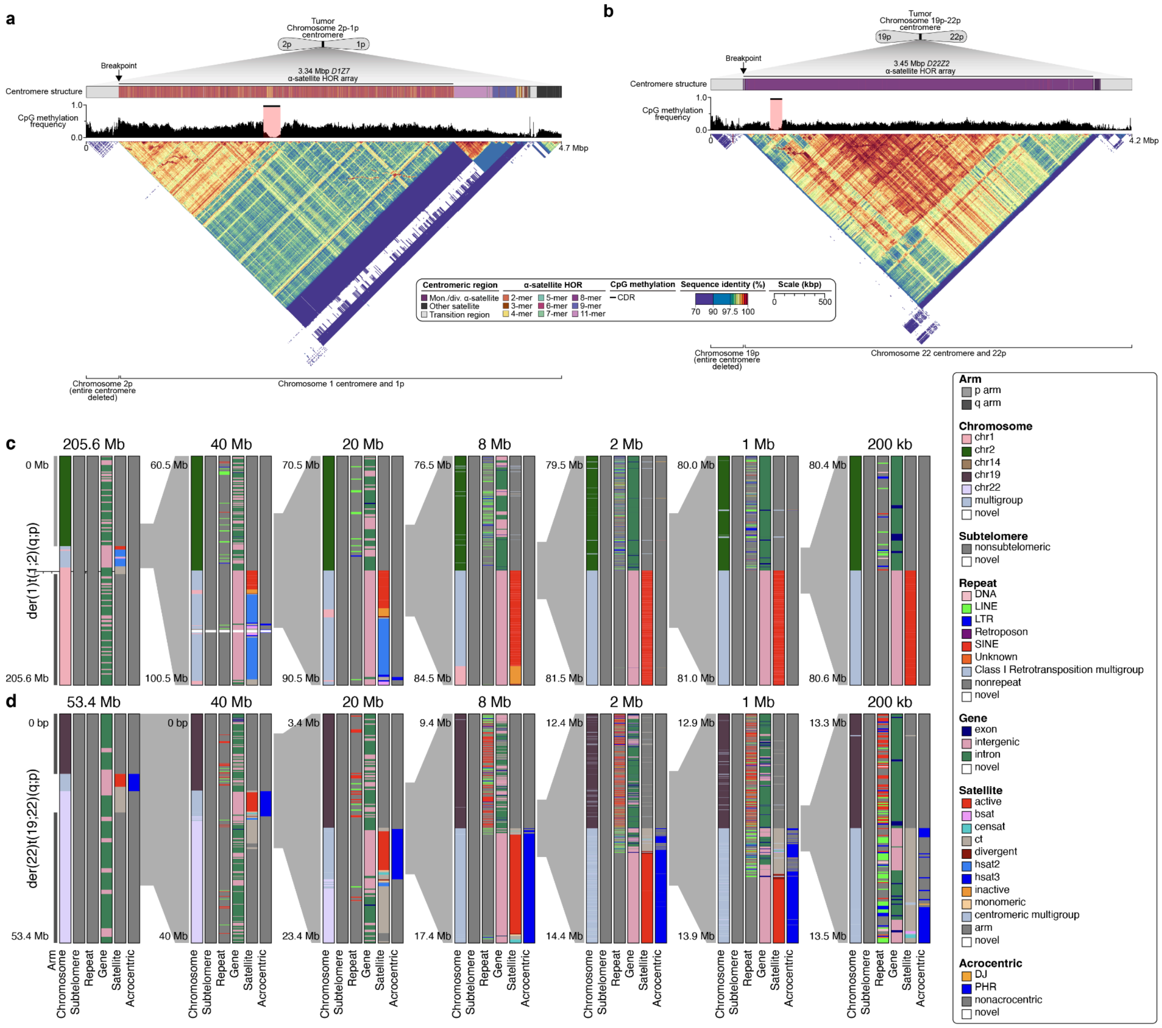

Supplementary Fig. 23. The sequence, structure, and epigenetic landscape of the chromosome 2p-1p and 19p-22p centromeres in the tumor. a) Upon translocation of chromosome 2p to 1p, the chromosome 2 centromere is deleted while the chromosome 1 centromere is retained, albeit truncated on the p arm-proximal side. b) Similarly, upon translocation of 19p to 22p, the chromosome 19 centromere is deleted, while the chromosome 22 centromere is retained, also with a truncation on the p arm-proximal side. c) Karyoscope's chromosome, subtelomere, repeat, gene, satellite, and acrocentric annotations are shown across the entire chromosomes and at different resolutions around each interchromosomal breakpoint.

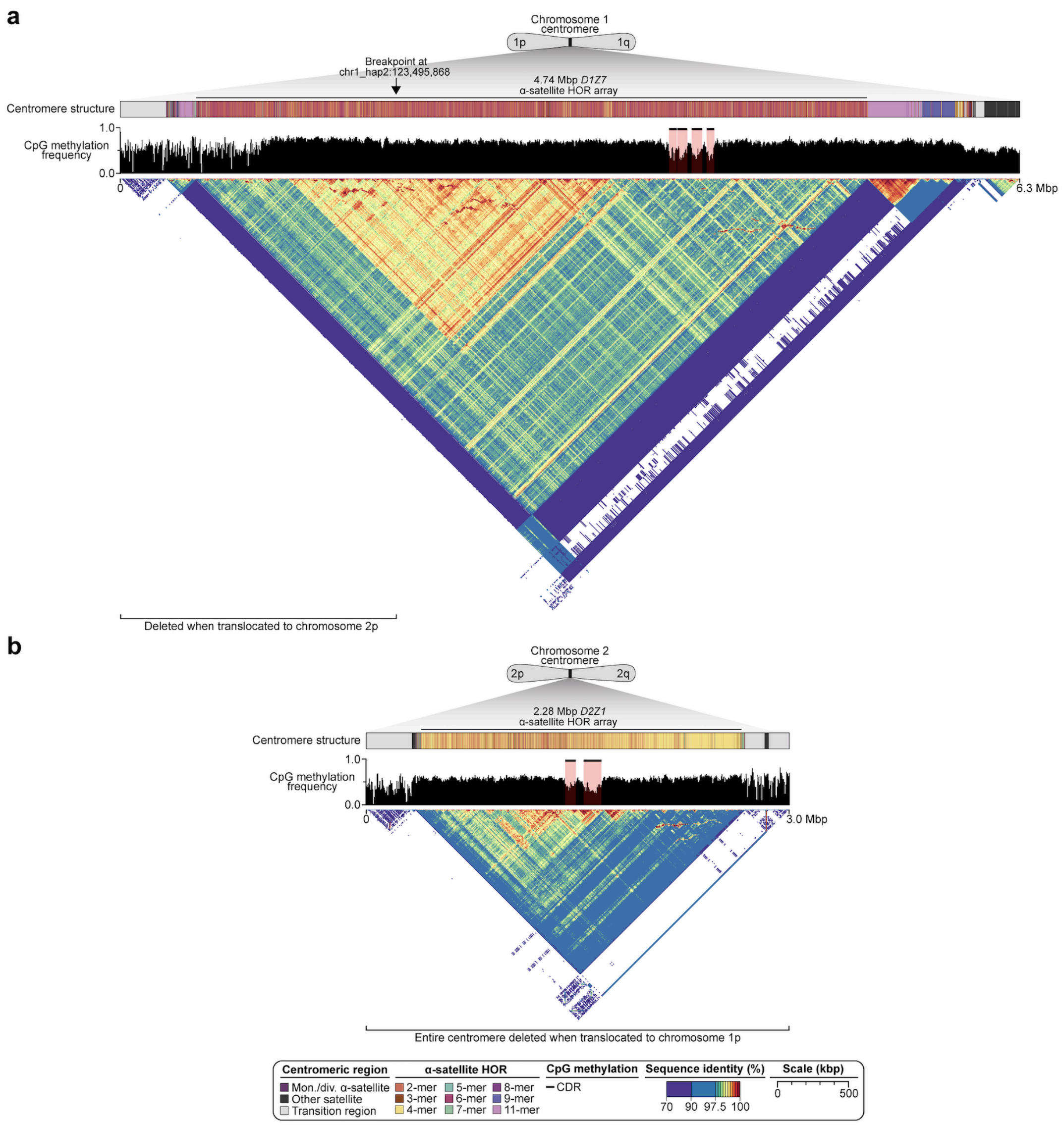

Supplementary Fig. 24. The sequence, structure, and epigenetic landscape of the chromosome 2 and 1 centromeres in normal cells. a) For chromosome 1, the CDR site is retained upon translocation. b) For chromosome 2, however, the entire centromeric α-satellite HOR array, including the CDR, is deleted upon translocation.

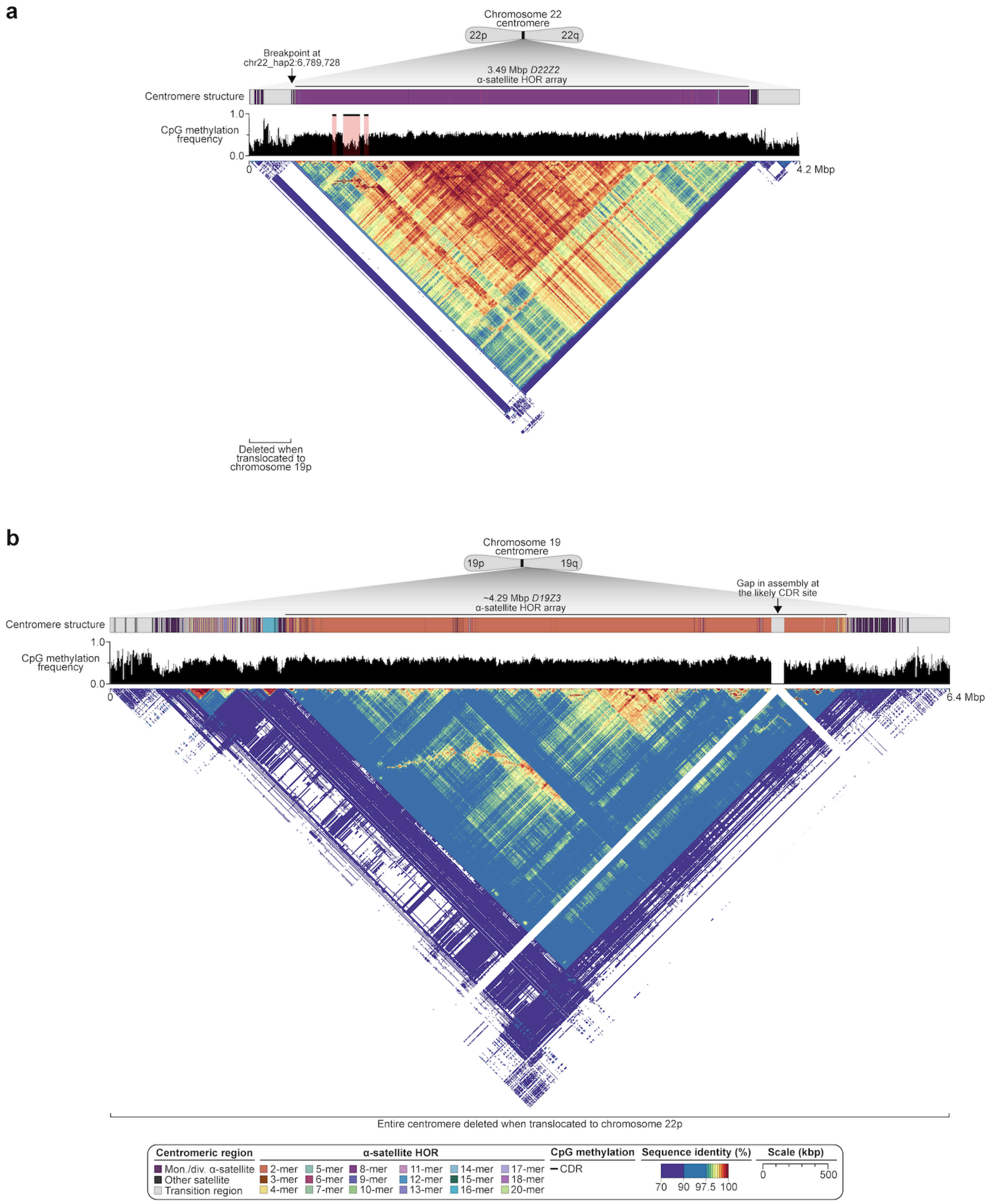

Supplementary Fig. 25. The sequence, structure, and epigenetic landscape of the chromosome 22 and 19 centromeres in normal cells. a) For chromosome 22, the CDR site is retained upon translocation. b) For chromosome 19, however, the entire centromeric α-satellite HOR array, including the CDR, is deleted upon translocation. The location of the CDR site likely resides in a gap in the assembly.

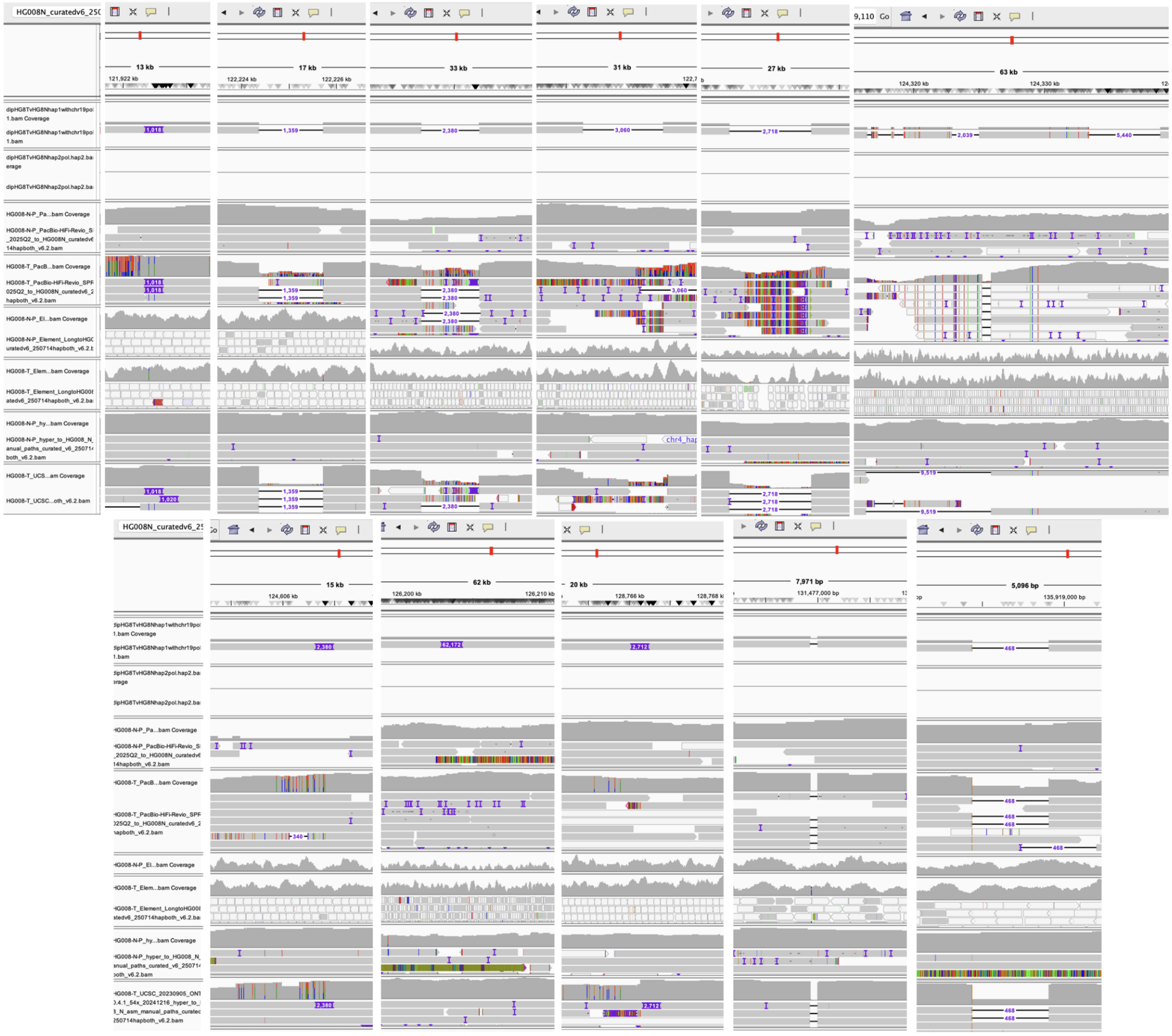

Supplementary Fig. 26: Complete chromosome 1 centromere comparison reveals four times more somatic SVs than in entire GRCh38 chromosome 1. While only three truncal SVs were identified on chromosome 1 haplotype 1 in GRCh38, the tumor vs normal assembly alignment of these haplotypes identified 12 additional likely truncal SVs in the CenSat region (as well as one in a small non-centromeric HSat3 array and one in the 5S rDNA array). We examined variants in the ~17 Mbp CenSat region of chromosome 1 haplotype 1, which is complete in both the tumor and normal. In the α-satellite HOR array, there are 4 insertions (1,018, 2,380, 12,240, and 62,172 bp in length) and 5 deletions (1,359, 2,039, 2,380, 2,718, 3,060, and 9,519 bp in length). There's also a 69 bp deletion of one β-satellite monomer, and a 2,712 bp insertion and 468 and 1,776 bp deletions in the HSat2 array. Four of the SVs have phased SNVs near the breakpoints, with many deletions having a SNV at the base immediately before the start of the deletion. In addition, there are 66 confirmed SNVs and 5 confirmed 1 bp indels in this ~17 Mbp CenSat region though some true variants may not have been confirmed due to mapping challenges. See support for SVs in https://github.com/jzook/HG008SVcuration/issues/327-338. A CenSat subclonal DEL is in https://github.com/jzook/HG008SVcuration/issues/340. While chr1 has a particularly large CenSat region with 12 SVs, this CenSat region is highly enriched for somatic SVs, while having similar SNV rate and lower indel rate than the rest of the chromosome. While acrocentric rDNA arrays are not resolved in the assemblies, the ~700 kbp 5S rDNA array on chr1 haplotype 1 (but not haplotype 2) is resolved in the tumor and normal assemblies, and it has a truncal somatic 2,232 bp deletion of one monomer unit (which includes a ~120 bp 5S rRNA gene and a 2.1 kbp intergenic spacer). The region near the middle of the array with the deletion is methylated in both tumor and normal, so it probably is inactive and unlikely to have an effect. Interestingly, the normal assembly enables us to see that while most of the array is nearly fully methylated, the ~40 kbp on the right side of the array is unmethylated, similarly in both tumor and normal https://github.com/jzook/HG008SVcuration/issues/339.

(a)

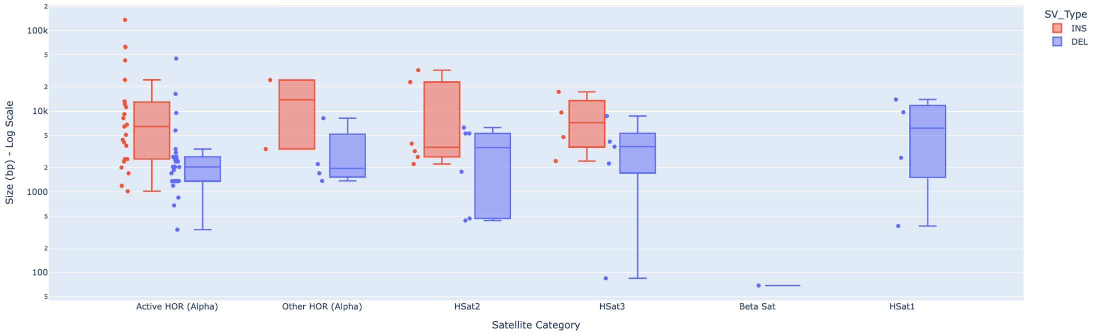

(b)

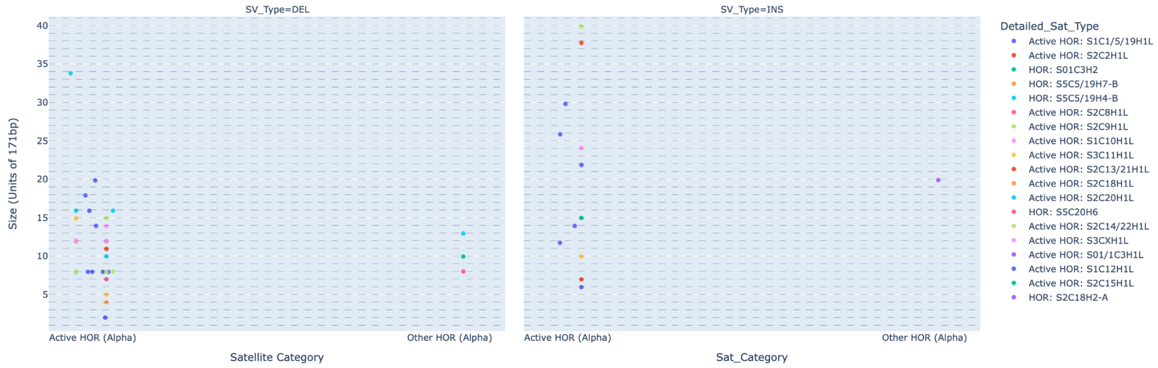

Supplementary Fig. 27: Centromere satellite SVs vary by repeat type and follow expected alpha satellite periodicity. (a) Analysis of 103 somatic insertions and deletions in centromeric and satellite DNA regions of HG008-T revealed two distinct patterns. First, somatic insertions were significantly larger than deletions across all satellite types (median: 5,776 bp vs 2,053 bp; Mann-Whitney U test, p = 1.20×10^{7}), with this size asymmetry particularly pronounced in α-satellites (median: 6,636 bp vs 1,952 bp; p = 1.09×10^{6}). The four largest confirmed somatic expansions of 61 kbp to 136 kbp are larger than germline or somatic de novo centromeric expansions seen in recent studies. This pattern may suggest that large insertions in HG008-T arise through tandem duplication mechanisms similar to those observed in non-repetitive regions. However, these are also challenging to detect without assembling both tumor and normal reads so may have been invisible to mapping-based approaches used in previous somatic studies. (b) Smaller somatic SVs (50 bp to 5 kb) in active higher-order repeat (HOR) α-satellites showed strong periodicity at 171 bp intervals, with 54.3% of variants falling within ±10 bp of an exact 171 bp multiple—a 4.6-fold enrichment over the 11.7% expected by chance (χ^2 = 81.03, p = 2.23×10^{19}). This periodicity was specific to α-satellites, as non-α-satellite variants showed no such pattern (11.1% near 171 bp multiples), consistent with replication slippage or unequal sister chromatid exchange involving whole α-satellite monomers (171 bp) or their higher-order multiples. These findings demonstrate that somatic structural variation in satellite DNA follows mechanistic constraints imposed by the underlying repeat structure.

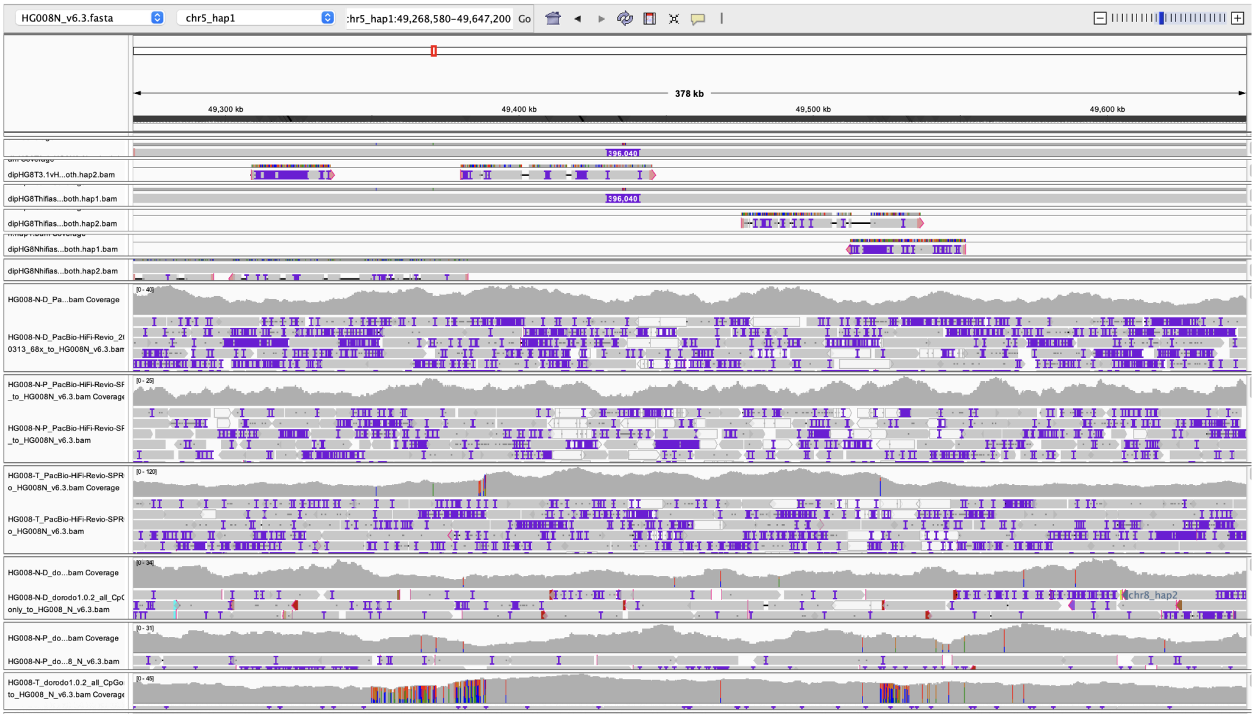

Supplementary Fig. 28: Large CenSat insertions. The largest CenSat insertion is a 136kbp tandem duplication on chr5_hap1 (https://github.com/jzook/HG008SVcuration/issues/351). The hifiasm assemblies match the v3.1 tumor and v6.3 normal assemblies, and it is also supported by the ~2 times higher coverage of the 136kb region in the middle, and clipped ONT reads in the tumor and not normal. A 375kb ONT read traverses one copy of the duplicated region and aligns ~60kb into the second copy since it noisily aligns ~60kb past the left breakpoint. Other large CenSat insertions >30kb include https://github.com/jzook/HG008SVcuration/issues/445, https://github.com/jzook/HG008SVcuration/issues/396, https://github.com/jzook/HG008SVcuration/issues/381, https://github.com/jzook/HG008SVcuration/issues/379, and https://github.com/jzook/HG008SVcuration/issues/334.

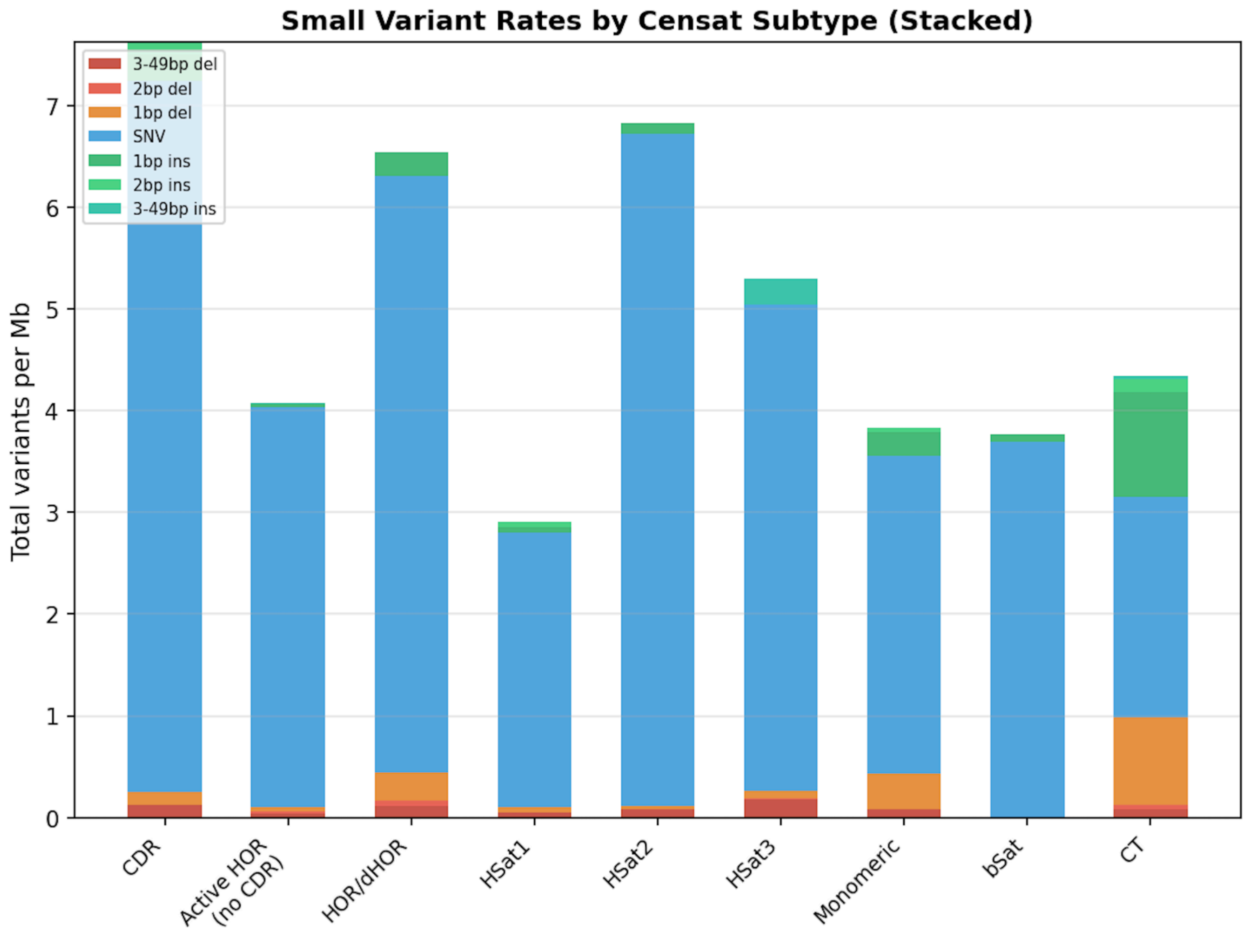

Supplementary Fig. 29: Centromere satellite small variant rates vary by type and are mostly SNVs

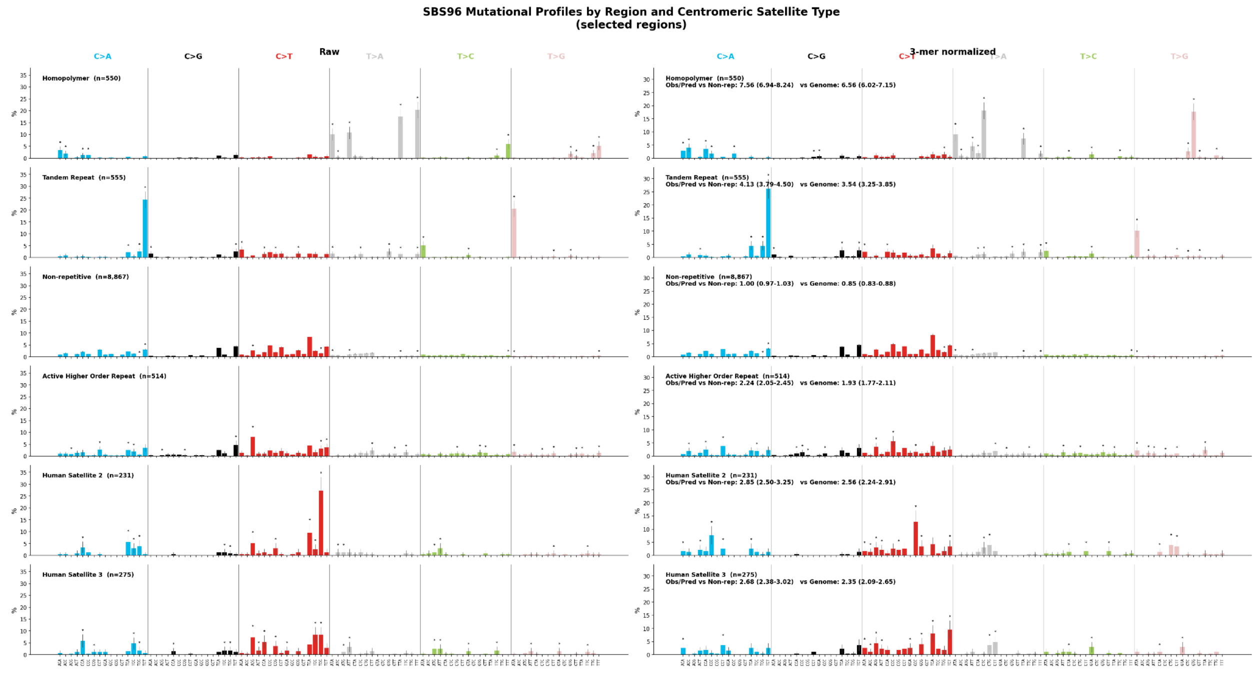

Supplementary Fig. 30: SBS96 mutational spectra across genomic regions, with and without trinucleotide frequency normalization. Mutational spectra also vary across repetitive regions, even after correcting for trinucleotide frequencies. Homopolymer regions show the largest excess (Obs/Pred = 7.56 vs non-repetitive), driven primarily by T>A and T>G mutations in adjacent homopolymers and homopolymers next to dinucleotide TRs. Human Satellite 2 (Obs/Pred = 2.85) is dominated by C>T transitions at CpG dinucleotides, indicative of 5-methylcytosine deamination in hypermethylated heterochromatin. Active Higher Order Repeats (Obs/Pred = 2.24) and Human Satellite 3 (Obs/Pred = 2.68) display broader mutational spectra suggesting contributions from both CpG deamination and replication-associated errors, possibly due to less efficient repair in late-replicating heterochromatin. All repeat regions show mutation rates significantly exceeding predictions from trinucleotide composition. Each row shows the SBS96 profile for a genomic region, with raw counts (left) and 3-mer normalized counts (right). Normalization corrects for differences in trinucleotide composition between regions by scaling each channel by the ratio of genome-wide to region-specific 3-mer frequency, preserving total mutation count. Error bars indicate 95% confidence intervals (binomial approximation). Asterisks denote channels where the per-base pair mutation rate is significantly different from the whole-genome rate (Poisson rate ratio z-test, 99% confidence). Panel annotations show the Obs/Pred ratio relative to non-repetitive and whole-genome baselines, where Observed is the measured SNV rate per bp and Predicted is the expected rate based on trinucleotide composition alone.

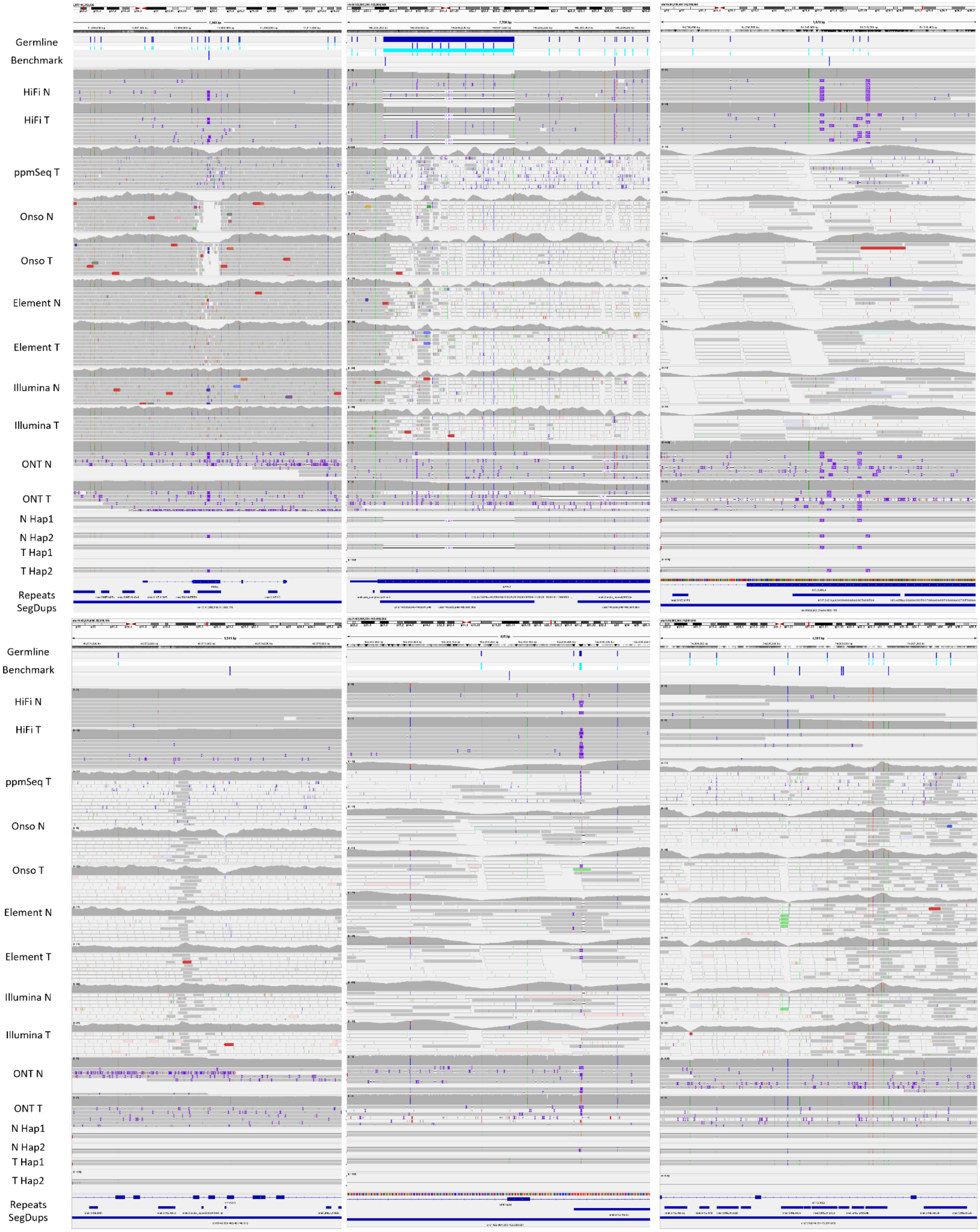
Germline
Benchmark
HiFi N
HiFi T
ppmSeq T
Onso N
Onso T
Element N
Element T
Illumina N
Illumina T
ONT N
ONT T
N Hap1
N Hap2
T Hap1
T Hap2
Repeats
SegDups

Supplementary Fig. 31: Assemblies resolve true somatic variants in segmental duplications: These regions typically have high false positive and false negative rates with mapping-based approaches. There are TR-associated variants in *PRB4*, *EPPK1*, *GOLGA6L4*, and as well as non-TR coding variants in PPIP5K1 (chr15:43573416), SPDYE2B exon 5, and a cluster of SNVs/indels in GTF2IRD2 (chr7:75074151-75217878). A particularly interesting TR example illuminated by the personalized reference is a stop-gain somatic SNV near the end of a 63 bp germline insertion in a VNTR in *PRB4* (BCSQ annotation of tumor insertion: stop_gained&inframe_insertion|PRB4|ENST00000279575|protein_coding|-|122R>122G*|11308619T>TTTCTGGCTTTCCTGGATGAGGTGGGGGACCTTGGGACTGGTTGCCTCCTTGTGGGGGTCATCC). The second codon from the end changes from CGA (arginine) in the normal to TGA (stop) in the reverse complement. While *PRB4* has only rarely been associated with cancer, this type of change would be very difficult to detect without long reads and a personalized reference. Relatedly, a coding VNTR in *EPPK1* has a synonymous SNV inside a 12 bp germline insertion (chr8:143863761 G>GGGCAGCGGCGGC), and a coding VNTR in *GOLGA6L4* has a synonymous SNV that causes a 21 bp germline insertion to shift alignment by 24 bp (chr15:84240066 G>GAACAGGAGGAGAGGCTGTGTA), both of which are difficult to detect with standard methods. *SPDYE2B* SNV in exon 5 that appears nonsynonymous but has no coverage in gnomAD due to low mappability.

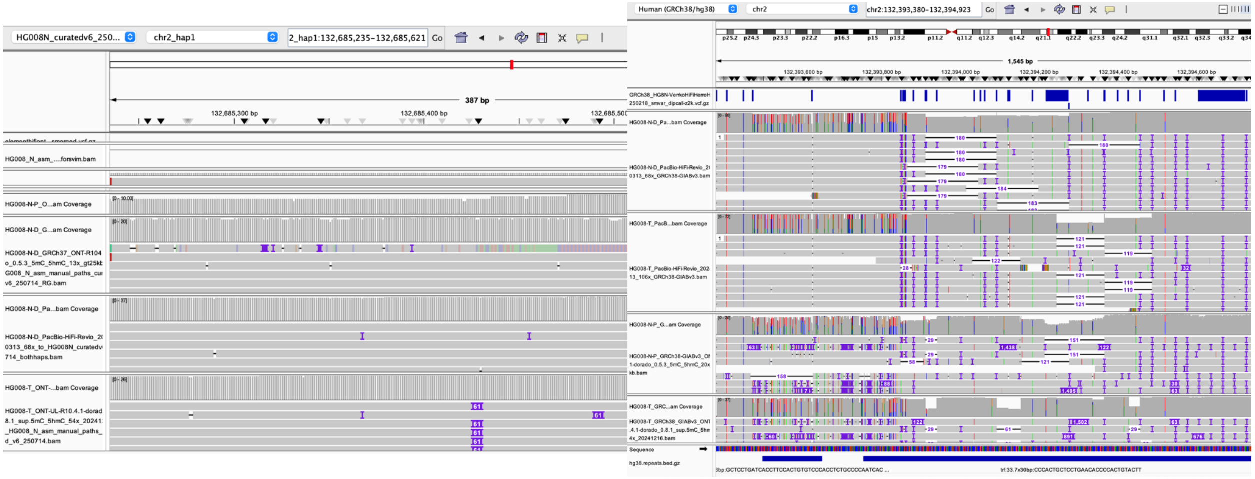

Supplementary Fig. 32 Large STR and VNTR Expansions and Contractions: These appear as simple insertions and deletions with respect to the normal assembly, but became complex with respect to GRCh38 due to overlap with germline variation. Most somatic variants in STRs and VNTRs were not identified by mapping-based approaches because all had germline SVs and/or large indels as well. Due to a 42bp germline insertion in one VNTR, a 70bp somatic deletion, which falls above the typical SV size cutoff, appears as a 28bp deletion with respect to GRCh38, so that somatic SV callers would typically ignore this. There's also a 60bp expansion of a VNTR around chr2:132394152 that contains very complex germline variation making it challenging to understand or represent the variant with respect to GRCh38, but it is very clear on the normal reference (https://github.com/jzook/HG008SVcuration/issues/323). In addition to VNTR expansions and contractions, breakpoints on one end of a complex 35Mbp somatic inversion on chr7 fall inside a 611bp VNTR that also has germline variation, which complicates detection and precise representation of the breakpoints on GRCh38, but breakpoints are clear when using the normal as the reference (https://github.com/jzook/HG008SVcuration/issues/199).

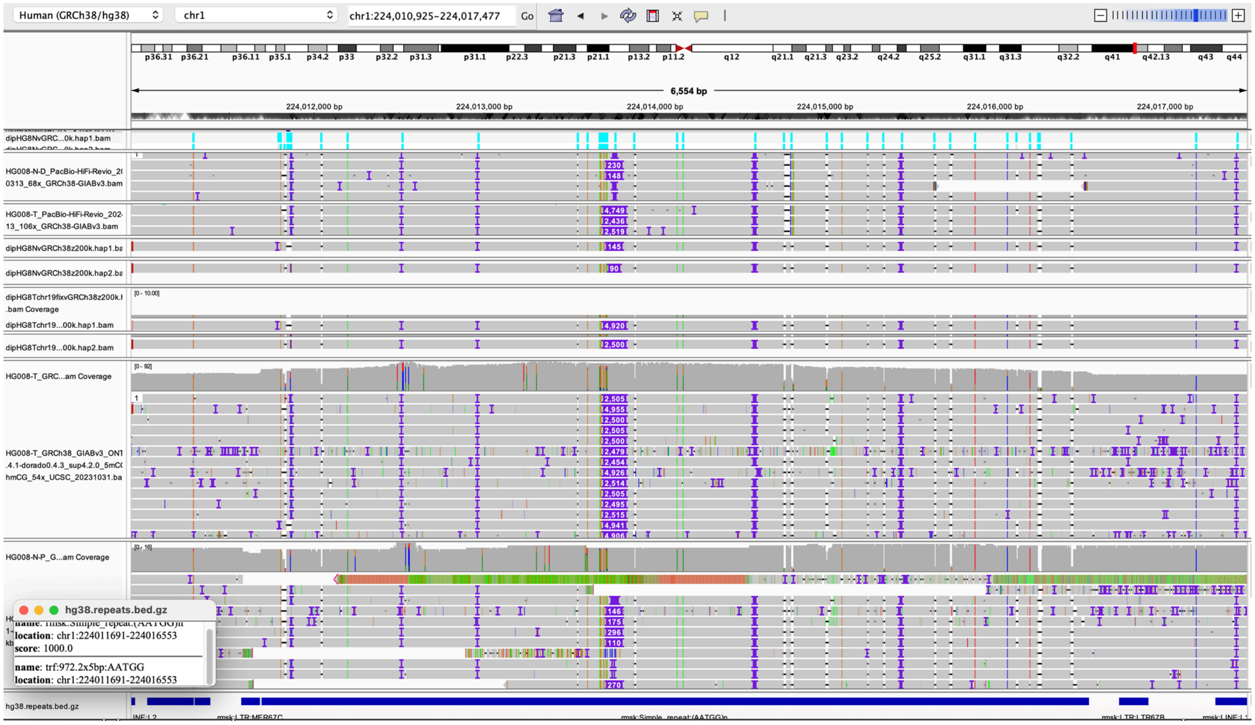

Supplementary Fig. 33: Large intronic tandem repeats. The assemblies resolve an intronic TR in the *SEPTIN7P13* segdup, which has ~4800bp somatic expansion on hap1 and ~2400bp somatic expansion on hap2 of the high-identity repeat in the middle of an imperfect repeat (https://github.com/jzook/HG008SVcuration/issues/326) we subsequently identified in a CenSat track as a small HSAT array. It also appears to have subclonal variability in both normal tissues whereas the tumor cell line has much less mosaicism even though the repeat is longer, so it may be a particularly unstable TR in normal tissues (similar to a TR on chr4 https://github.com/jzook/HG008SVcuration/issues/386). This TR is also challenging because extra HiFi reads mismap here on GRCh38 that match a segdup at the beginning of chr1 on the normal assembly. The assemblies help both in minimizing mismapped reads as well as enabling resolution of the different expansions on each haplotype. There is an interesting CT-rich VNTR at chr6_hap2:101427602 that seems to have a somatic 60bp INS supported by almost all tumor reads, but the normal pancreas HiFi and ONT have a mix of lengths ranging from no INS to ~60bp. Most insertions and many deletions 4 to 49 bp in size also fall in TRs, as described in the small variants section.

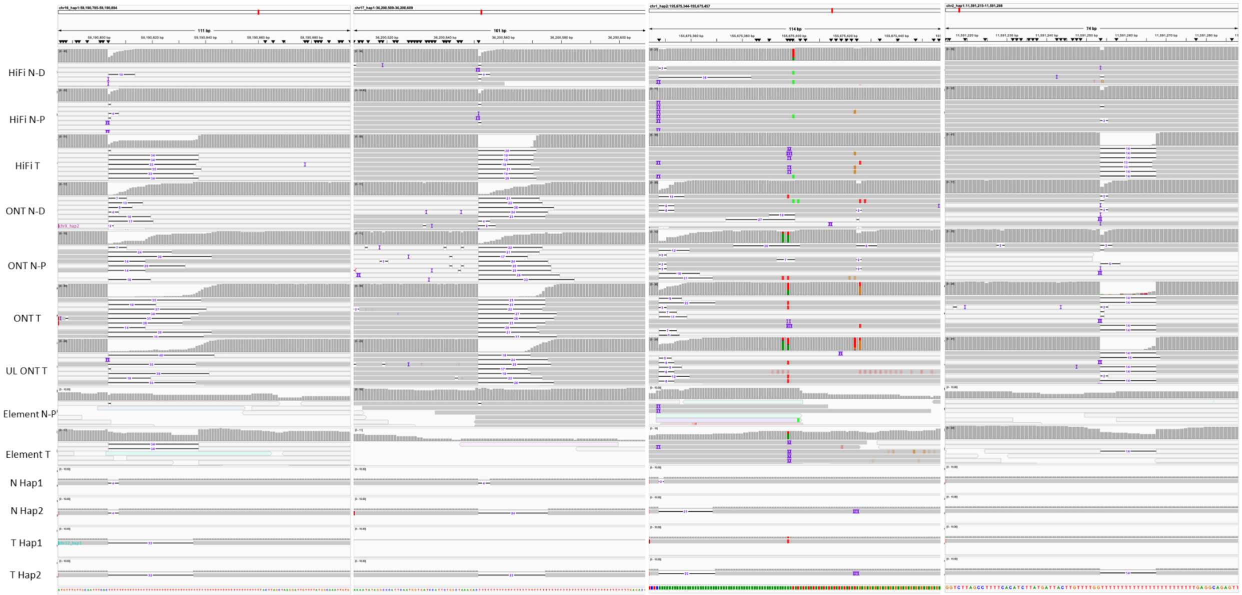

Supplementary Fig. 34: Large Indels in Homopolymers and diTRs. When aligning to both haplotypes of HG008-N, several deletions and insertions are evident. Deletion examples include a somatic 34bp contraction of 58bp homopolymer at chr16_hap1:59190801 and a 19bp contraction of 52bp homopolymer at chr17_hap1:36200559. Also, 7 contractions of 10-30bp in homopolymers longer than 50bp are present in the benchmark. Further, there is a 7bp INS at a 52bp homopolymer 27bp diTR junction at chr1_hap2:155675392. For deletions, there are 14 deletions >=10bp in homopolymers <50bp, with the shortest being a 14bp contraction of a 24bp homopol at chr2_hap1:11591252. In contrast, all 5 insertions >10bp in homopolymers appeared likely to be assembly errors, mostly haplotype switches in the normal assembly.

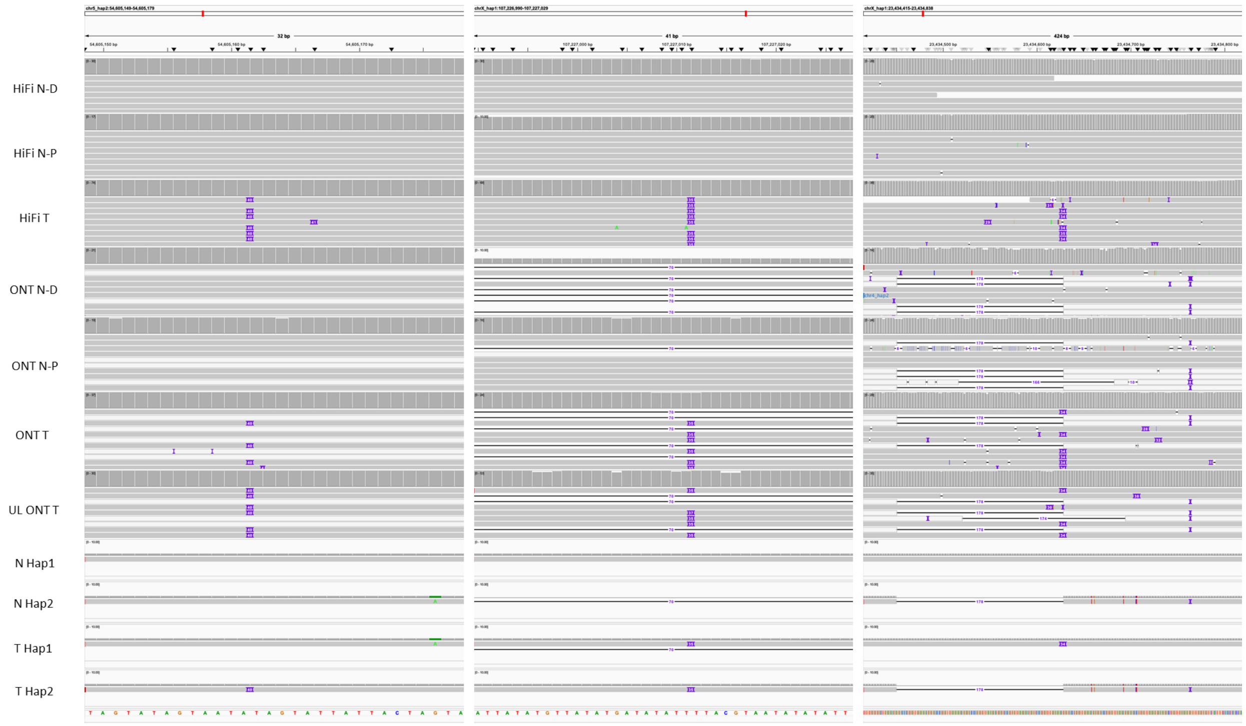

Supplementary Fig. 35: 30-40 bp insertions in VNTRs. The following VNTRs are evident in the benchmark: a 40bp INS at chr5_hap2:54605160, a 35bp INS at chrX_hap1:107227010, and a 34bp INS at chrX_hap1:23434627.

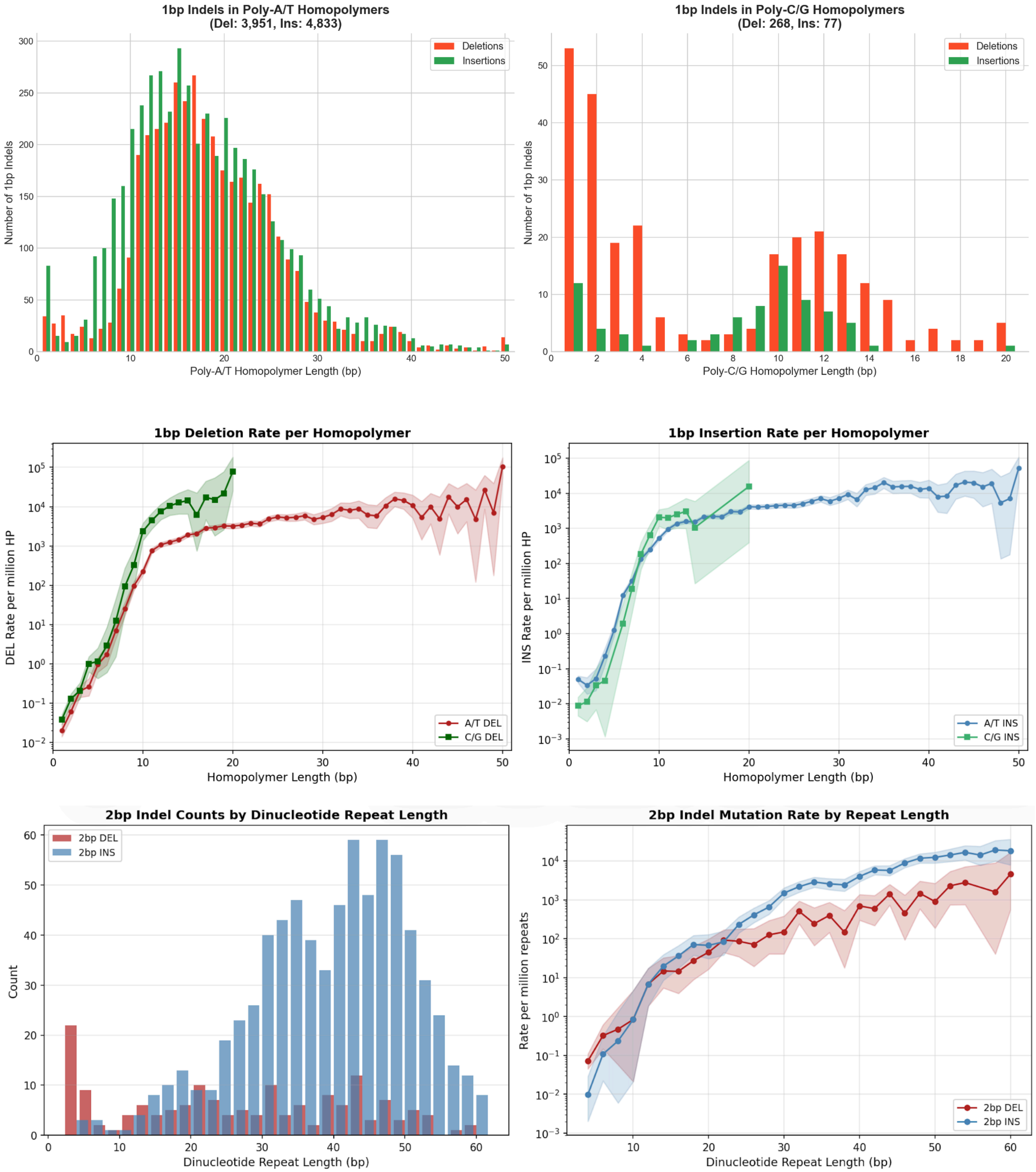
1bp Indels in Poly-A/T Homopolymers
(Del: 3,951, Ins: 4,833)
Deletions
Insertions
Number of 1bp Indels
Poly-A/T Homopolymer Length (bp)
1bp Indels in Poly-C/G Homopolymers
(Del: 268, Ins: 77)
Deletions
Insertions
Number of 1bp Indels
Poly-C/G Homopolymer Length (bp)
1bp Deletion Rate per Homopolymer
DEL Rate per million HP
A/T DEL
C/G DEL
Homopolymer Length (bp)
1bp Insertion Rate per Homopolymer
INS Rate per million HP
A/T INS
C/G INS
Homopolymer Length (bp)
2bp Indel Counts by Dinucleotide Repeat Length
2bp DEL
2bp INS
Count
Dinucleotide Repeat Length (bp)
2bp Indel Mutation Rate by Repeat Length
Rate per million repeats
2bp DEL
2bp INS
Dinucleotide Repeat Length (bp)

Supplementary Fig. 36: Somatic indel rates increase with homopolymer and dinucleotide TR length. Indel mutation rates per homopolymer base or stretch both increase with homopolymer length, with exponential increase from 1 to 11 bp A/T and G/C homopolymers. At the same length of homopolymers, mutation rates are higher for G/C than A/T, particularly for 1 bp deletions, though far fewer G/C homopolymers are in the human genome. A total of 345 1 bp indels occurred in C/G homopolymers (268 deletions, 77 insertions; overall DEL/INS = 3.48, 95% CI [2.70-4.48]). The relationship between homopolymer length and indel type was non-monotonic. While 10bp C/G homopolymers showed balanced insertion and deletion counts (DEL/INS = 1.13, 95% CI [0.57-2.27]), shorter homopolymers (<7bp) showed a strong deletion bias (DEL/INS = 6.73, 95% CI [4.30-10.53]), and longer homopolymers (>10bp) also showed a strong deletion bias (DEL/INS = 4.09, 95% CI [2.59-6.45]). The 7-9bp length range showed a trend toward insertion bias that was not statistically significant (INS/DEL = 1.89, 95% CI [0.84-4.24]). A total of 8,784 1bp indels occurred in A/T homopolymers (3,951 deletions, 4,833 insertions; overall INS/DEL = 1.22, 95% CI [1.17-1.28]). The insertion/deletion ratio varied substantially with homopolymer length. Short A/T homopolymers (≤13bp) showed a significant insertion bias (INS/DEL = 1.70, 95% CI [1.57-1.84]), with the strongest bias at 7bp (INS/DEL = 4.55, 95% CI [2.87-7.21]). In contrast, longer A/T homopolymers (≥15bp) showed only a slight insertion bias (INS/DEL = 1.07, 95% CI [1.02-1.13]). Interestingly, homopolymers of 33-37bp showed an unexpected insertion excess (INS/DEL = 1.93, 95% CI [1.46-2.55]), with individually significant excesses at lengths 35bp (INS/DEL = 3.30, 95% CI [1.63-6.70]) and 36bp (INS/DEL = 2.60, 95% CI [1.25-5.39]).

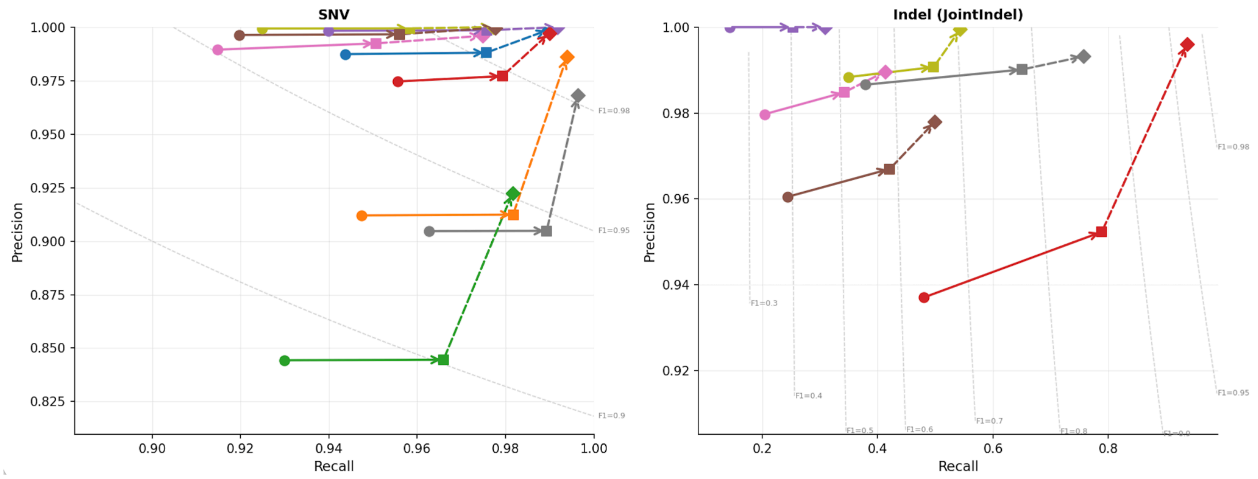

Supplementary Fig. 37 Accuracy is lower for somatic variants near germline variants. (a) Performance across variant callers appears better when somatic variants near germline variants are excluded (diamonds), or somatic variants whose representation is affected by germline variants are excluded (squares), vs. all GRCh38 benchmark somatic variants (circles).

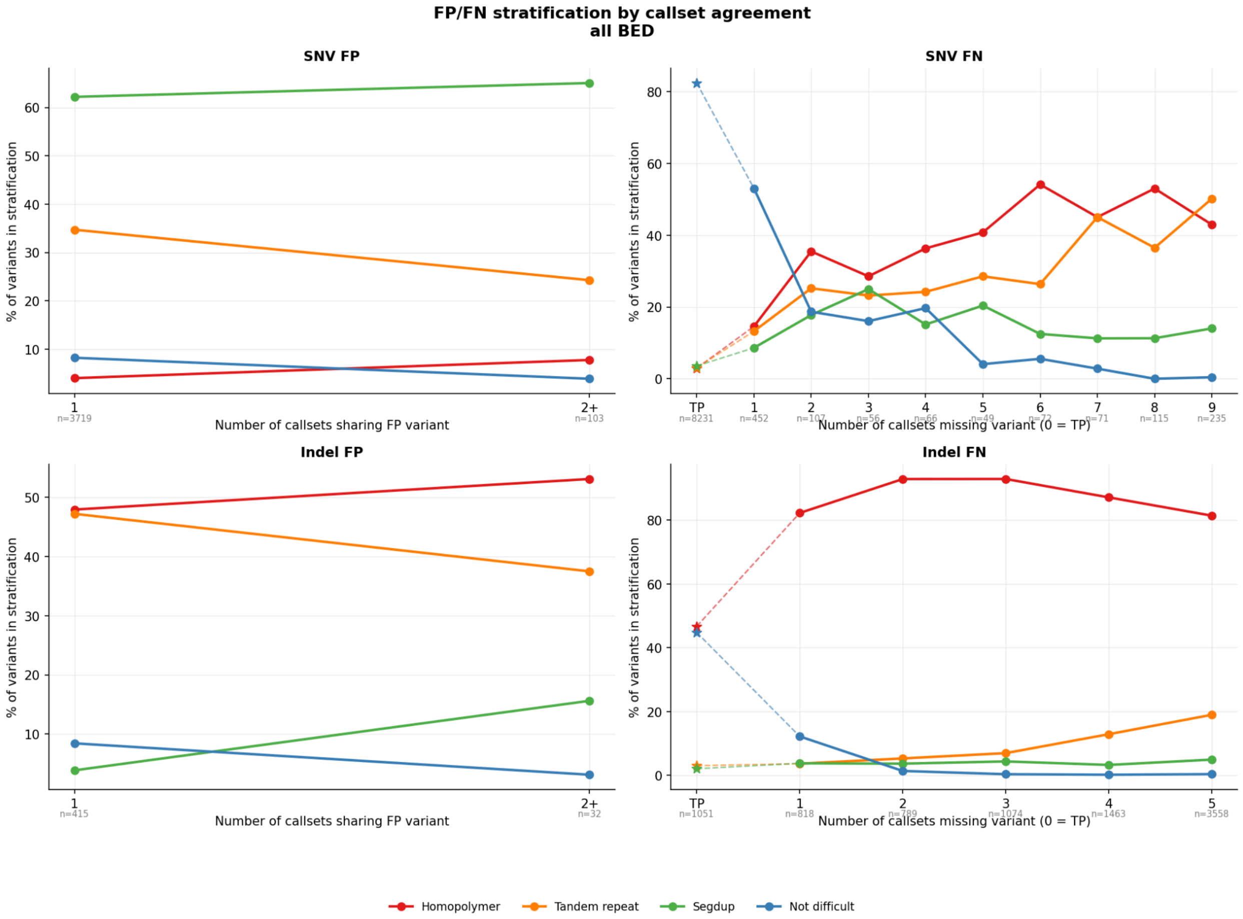

Supplementary Fig. 38 sources of FPs and FN. Plotting the SNV and Indel FPs and FNs across different stratifications shows the impact of genomic context such as repetitive regions on variant calling performance.

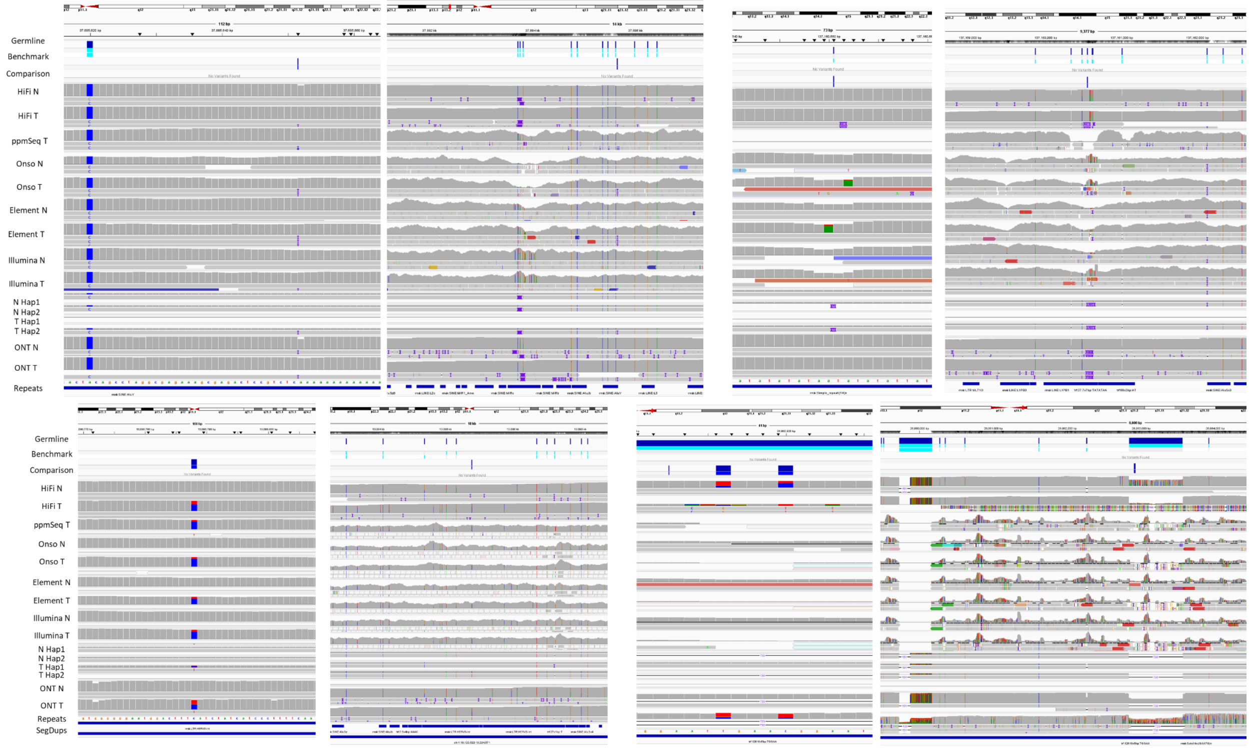

Supplementary Fig. 39: False Positive and False Negatives Commonly Occur in Repetitive Regions From visually inspecting over 180 discrepant small variants when comparing callsets to the draft benchmark, we identified that most of the discrepant calls are in repetitive regions such as homopolymers, SINEs, LINEs, tandem repeats, and segmental duplications. In this process, we considered a draft benchmark variant correct if the variant was supported by alignments of the tumor but not normal assemblies, and by tumor but not normal reads. We focused on reads from technologies with no evidence of bias or errors at the variant position, such as long reads in low mappability regions and segmental duplications or short reads that map across the repetitive unit in homopolymers or tandem repeats. Commonly, we identified that comparison callsets contained false positive somatic variants that matched or were near a germline variant in the normal sample. As a result of this process, we confirmed the benchmark reliably identified false positives and false negative SNVs and INDELs across comparison callsets. Examples include: (upper left) Homopolymers and SINEs - false negative 1bp insertion located in a SINE that is representative of many homopolymer FNs being in the polyA of Alus; (upper right) Tandem Repeats - false positive matches germline 14bp insertion in a tandem repeat and an LOH region; (lower left) Segmental Duplications - false negative due to difficult read mapping in Segmental Duplication; and (lower right) Small Variants within germline SVs – false positive SNV due to misaligned reads in germline 730bp deletion in a TR.

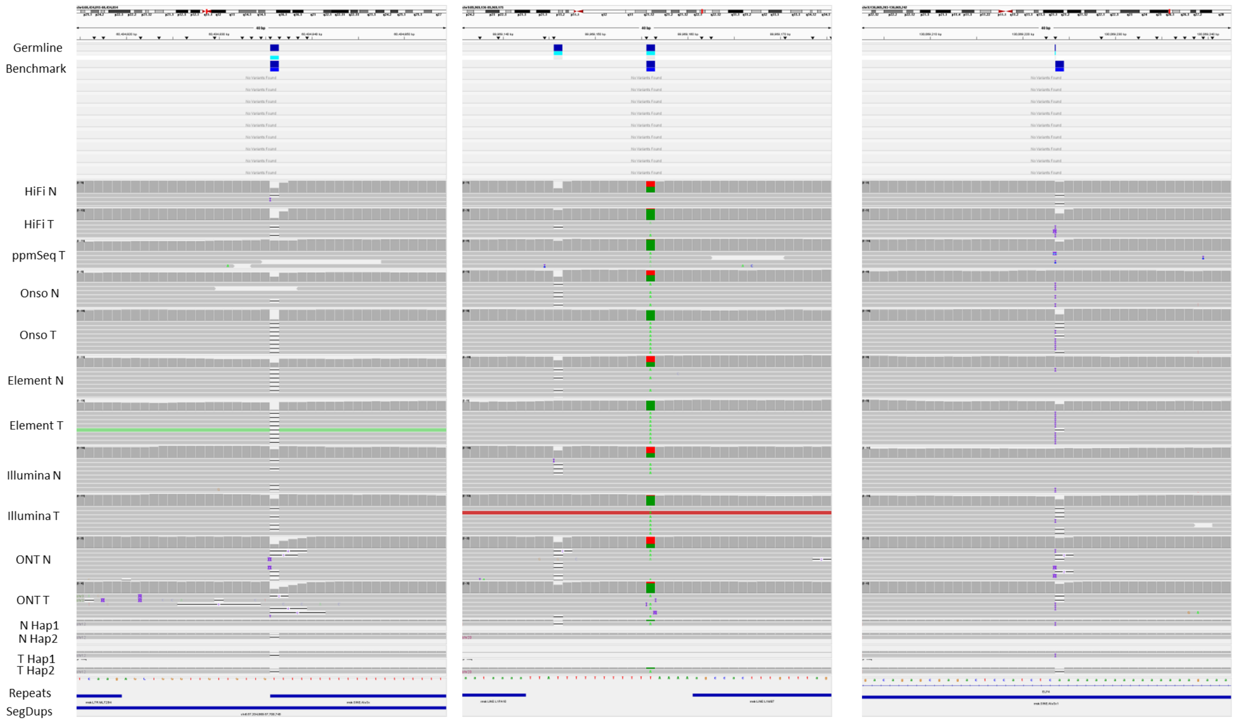

Supplementary Fig. 40 Variants Missed All Mapping-Based Methods. A class of somatic variants that are commonly missed in comparison between mapping-based methods and the somatic variant benchmark are those that modify germline variants, which usually occur in homopolymers and tandem repeats. These include the following types of modifications: those loci that appear to not be a variant in the tumor due to reversion of a germline variant to reference; those changing a germline variant to different variant; those that convert germline heterozygous to homozygous variant that looks similar to LOH (these appear in the current VCF file as heterozygous calls due to haplotype-specific calling); and other germline variation that makes it difficult to see true somatic change. There are also a small number of sites with somatic variants on both haplotypes.

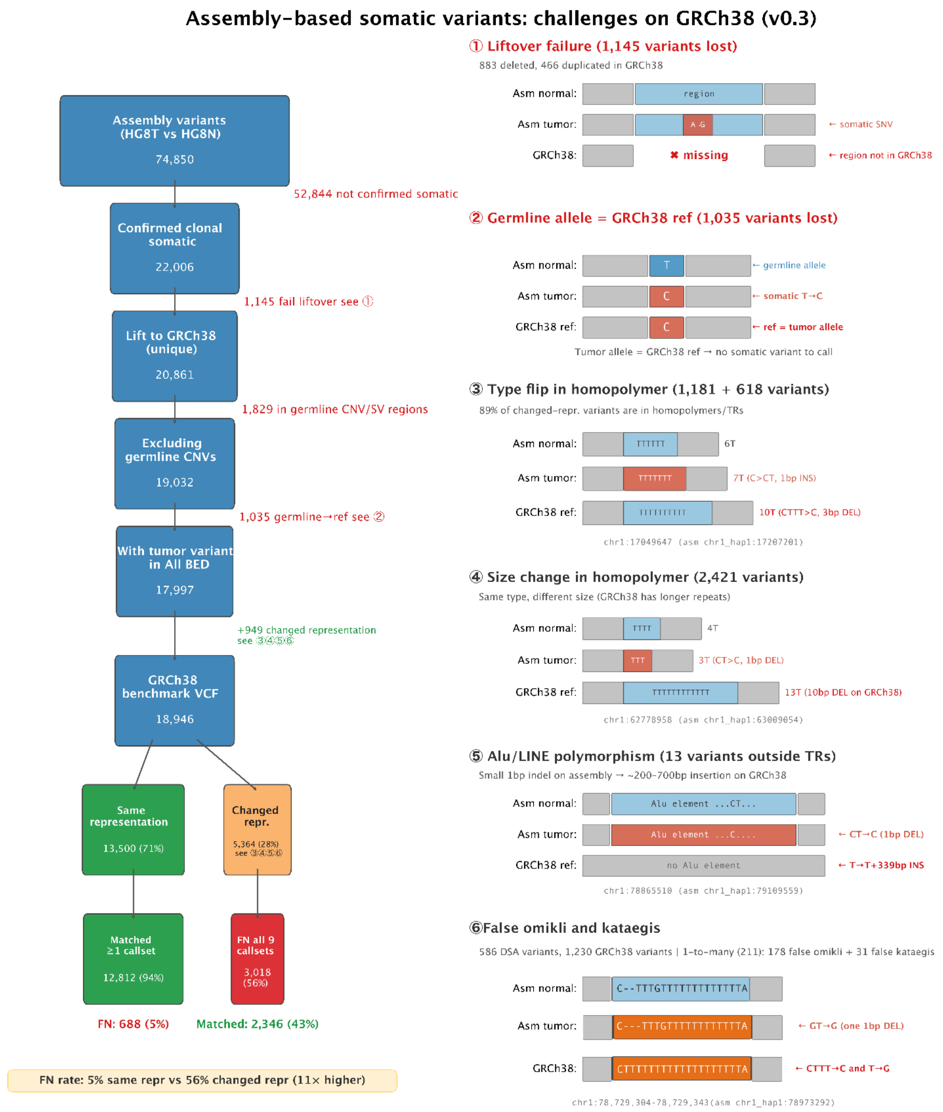

Supplementary Fig. 41: Small variants identified in tumor to normal assembly comparison are frequently challenging on GRCh38. The left side of the figure depicts the process by which the tumor and normal assemblies were compared yielding a set of clonal variants that were lifted over uniquely to GRCh38 and excluded germline CNVs. Highlights from the right side of the figure include 1) 1,359 variants were lost during liftover to GRCh38 and 2) 1,035 somatic variants have no tumor variant on GRCh38. While loss of germline variants is typically presumed to result from large deletions of the allele containing the variant, the phased tumor to normal assembly comparison shows these are actually small somatic variants causing reversion of a germline variant to the reference, frequently in homopolymers, similar to stochastic events in MSH6 and MSH3 homopolymers.[91] Related to the high germline and somatic mutation rates at homopolymers and TRs, 19 locations have different somatic indels at the same position on each haplotype at sites that also have germline multiallelic indels and were confirmed by curation.

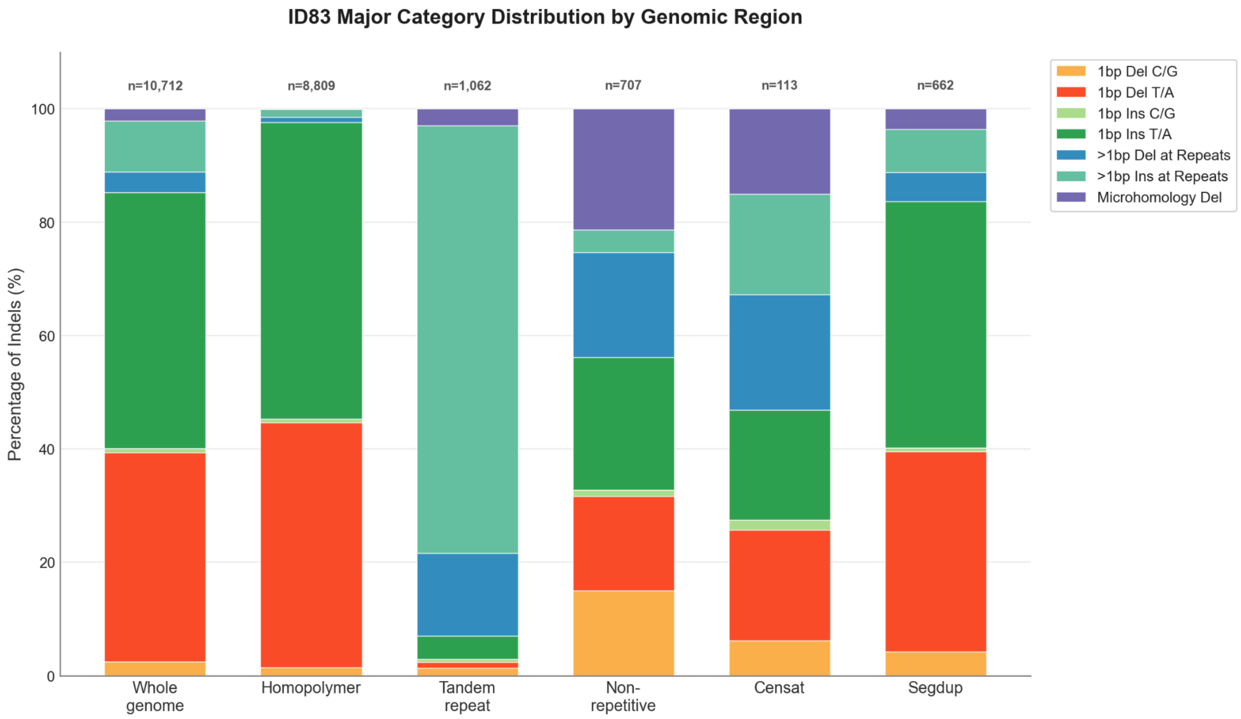

Supplementary Fig. 42: ID83 mutational spectra across genomic regions Indel signatures also vary across repetitive regions. In particular, homopolymers are highly enriched for 1 bp indels and STRs and VNTRs are highly enriched for >1 bp indels, particularly insertions. Because centromeric satellites have fewer homopolymers, STRs, and VNTRs, they are enriched for larger indels and microhomology-mediated deletions, similar to non-repetitive regions. The signatures can also change on a donor-specific reference vs GRCh38 because 1 bp somatic indels can change representation to larger indels or SNVs on GRCh38 due to germline variation.

.

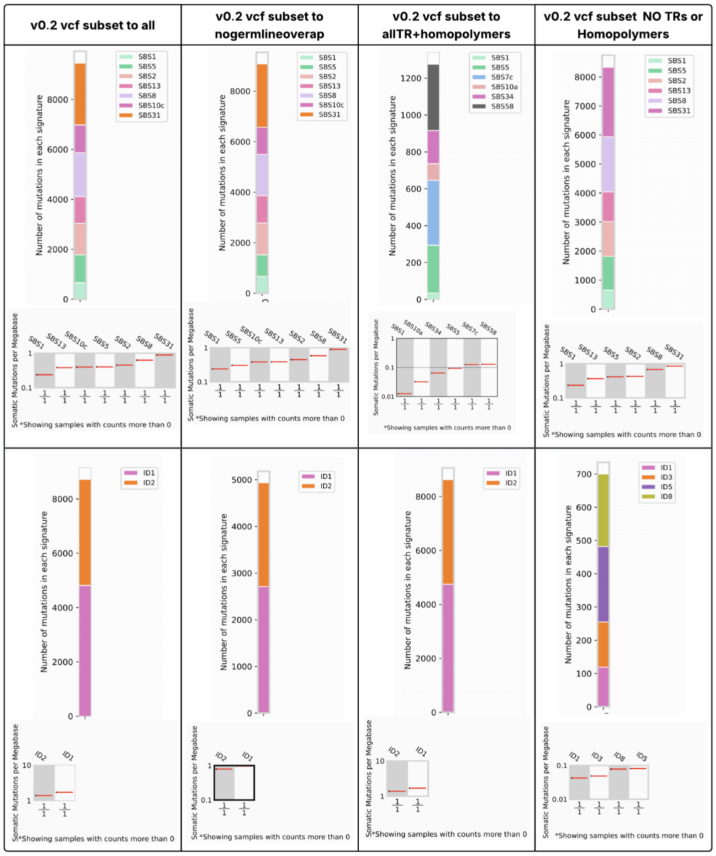

Supplementary Fig. 43: Mutational Signatures in GRCh38 benchmark. Using SigProfilerAssignment with the v0.2 small variant vcf subset to all benchmark regions (all), regions excluding somatic variants that alter germline variants (nogermlineoverlap), Tandem Repeats and Homopolymers (allTR+homopolymers), and outside Tandem Repeats and Homopolymers (No TRs or homopolymers). Single base substitutions (SBS) results for all.vcf seem consistent with PDAC (SBS1, 2, 5, 8, 13), and SBS31 is associated with platinum chemotherapy treatment which matches HG008-T intervention history. When subsetting to homopolymers and TRs, all of the signatures except SBS1 and SBS5 are enriched for motifs that occur at homopolymer/homopolymer, homopolymer/diTR, and diTR/diTR junctions where SNVs in these repeats often seem to occur. Small insertions and deletions (ID) mutational signature results show different patterns than other PDAC samples that are likely the result of more of these types of variants in the v0.2 small variant benchmark. ID1/ID2 median somatic mutations per megabase are around 0.1 and 0.09, respectively for the 240 PDAC samples while HG008-T exhibits rates closer to 1 for ID1 and ID2. Examining the entire vcf, subset to benchmark regions, and subset to TR/homopolymers as well as outside TR/homopolymers shows this is almost entirely due to the large number of variants in TR/homopolymers detected by our approach. Beyond ID1 and ID2, after removing the vast majority of indels that occur in homopol/TRs, increased rates of ID3 and ID5 are probably mostly homopolymers <7bp, which are not in the homopol/TR stratification bed files, and ID8 is larger deletions outside repeats.

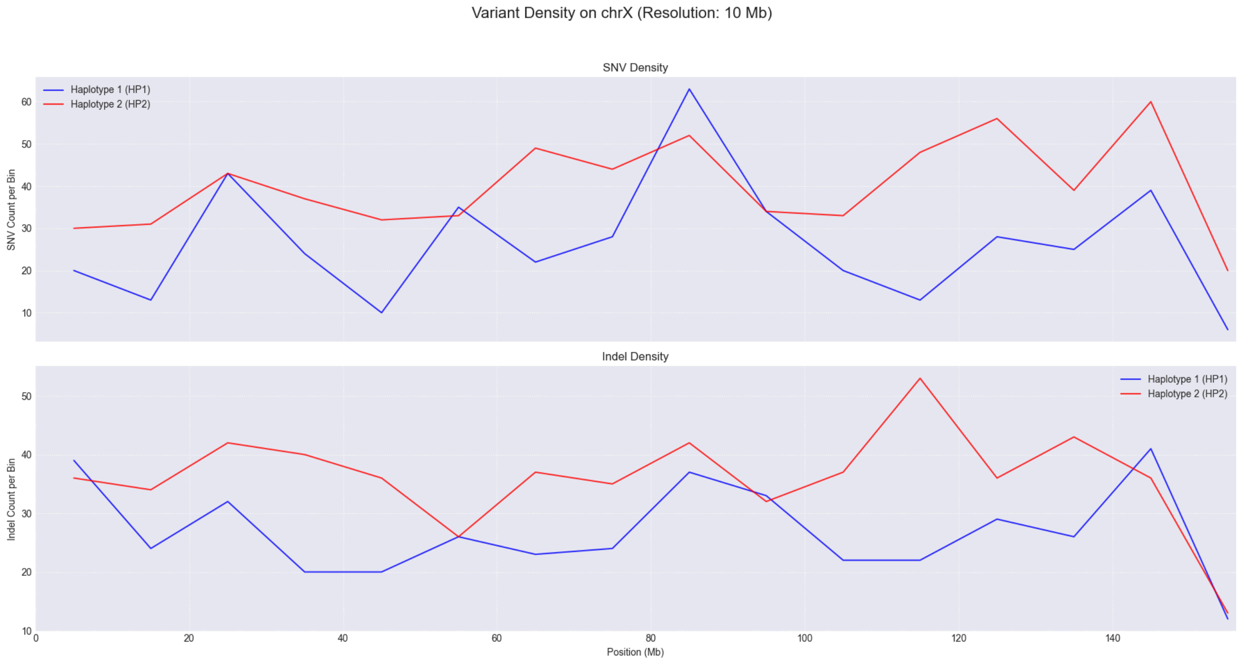

Supplementary Fig. 44: chrX Variant Density. Somatic variant density of Chromosome X HG008Nv6.3 assembly. Somatic SNVs and indels are generally evenly distributed across haplotypes and chromosomes that remain in the tumor, with a few notable exceptions. The inactive hap2 X has more SNVs and indels across most of the chromosome, consistent with previous studies showing hypermutation of inactive X is common in cancer.[25] Variant density is plotted as counts of SNVs and INDELs per bin across the chromosome position with the blue line for variants on Haplotype 1 and red line for those on Haplotype2. The red line is higher than the blue line for a majority of the chromosome and shows higher density on inactive Haplotype 2 of chrX.

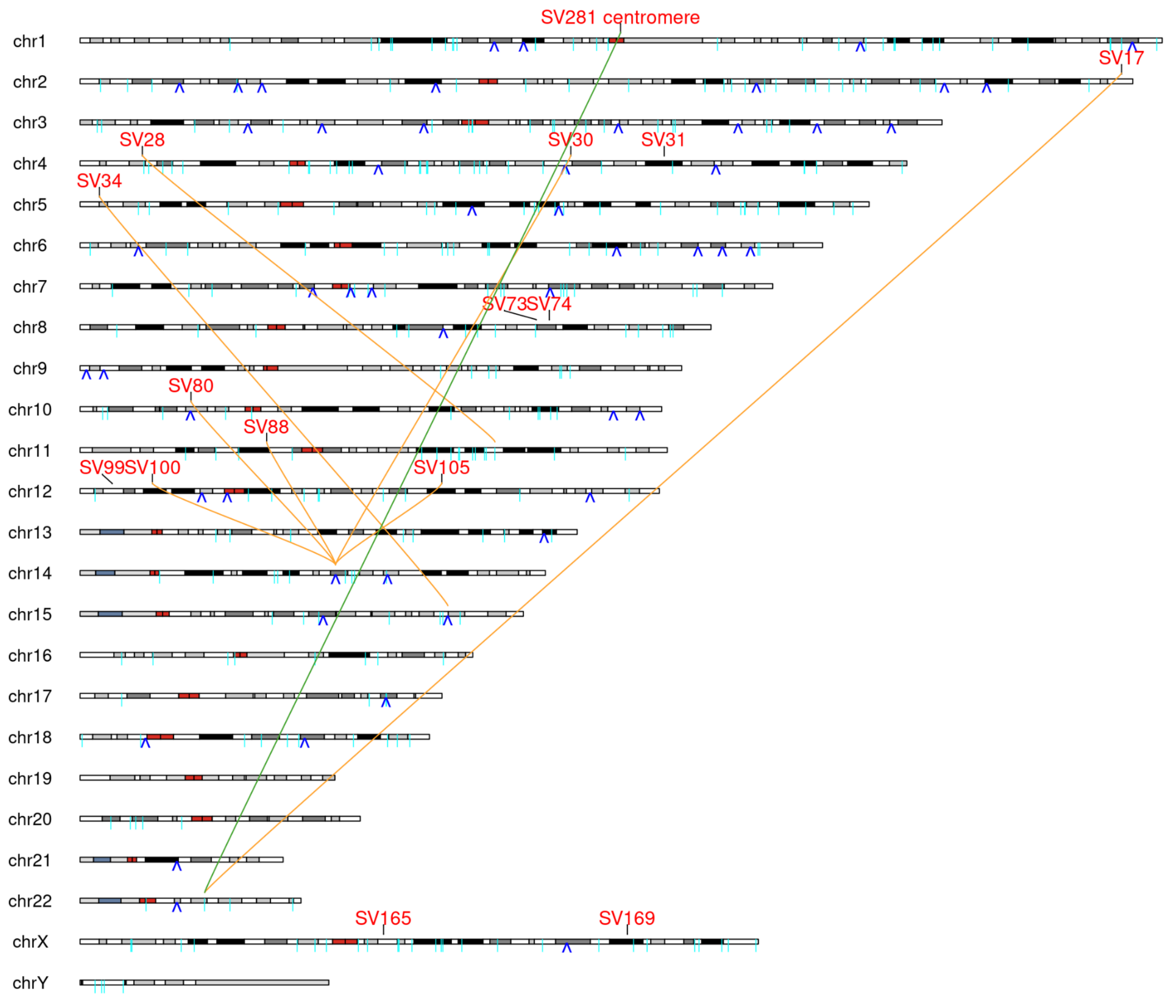

Supplementary Fig. 45: Location of LINE retrotranspositions and their source (where determined) yellow and green lines. Green line indicates insertion into chr1_hap2:124339632 (HG008Nv6.3). Cytoband data: http://hgdownload.cse.ucsc.edu/goldenpath/hg38/database/cytoBand.txt.gz. Cyan line markers: GRCh38 LINE1 locations taken from https://www.repeatmasker.org/genomes/hg38/RepeatMasker-rm406-dfam2.0/hg38.fa.out.gz. Blue inverted V markers: LINE1 locations from HG008-N-P HiFi pbsv Normal VCF.

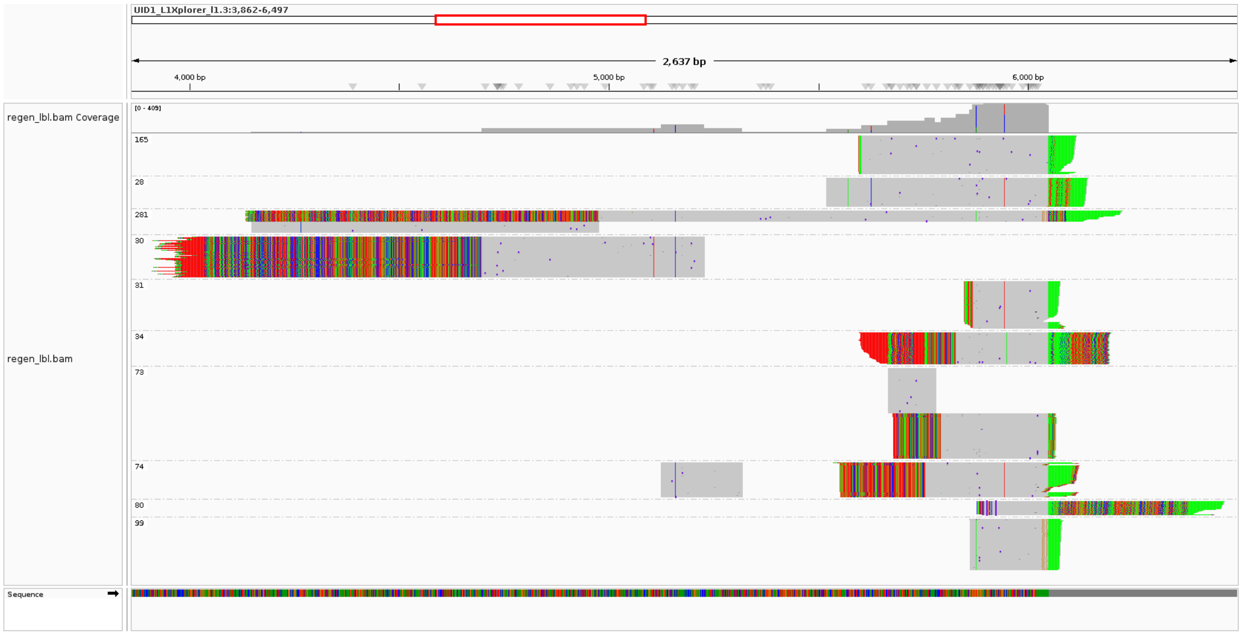

Supplementary Fig. 46: Somatic L1 insertions mapped to a reference L1 sequence. poly-A tails visible as green or red at end of insertions. Numbers are SV IDs from Supplementary Tables S8 and S9. Twin-priming events are also visible on 30, 34, 73, 74 and 281.

Normal pancreatic tissue ONT-based telomere lengths:

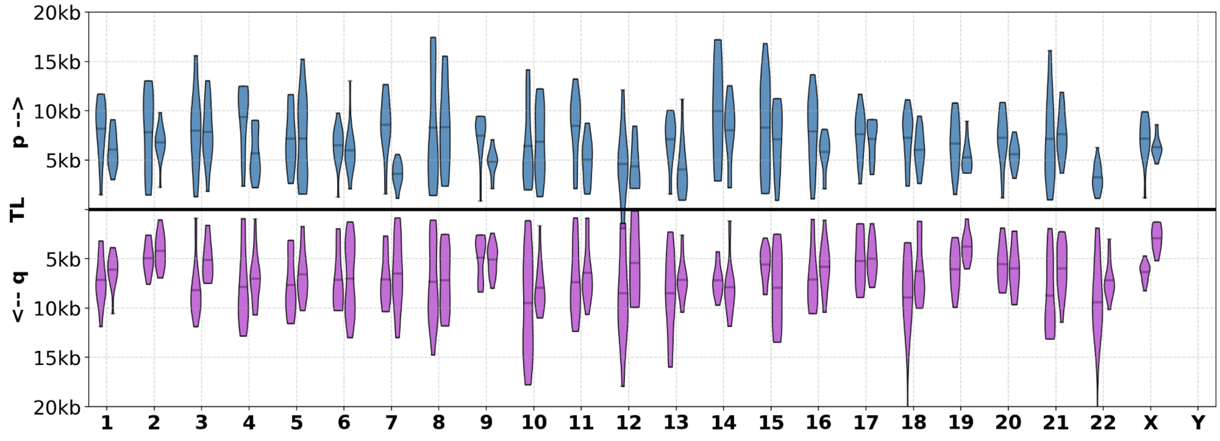

Tumor cell line ONT-based telomere lengths:

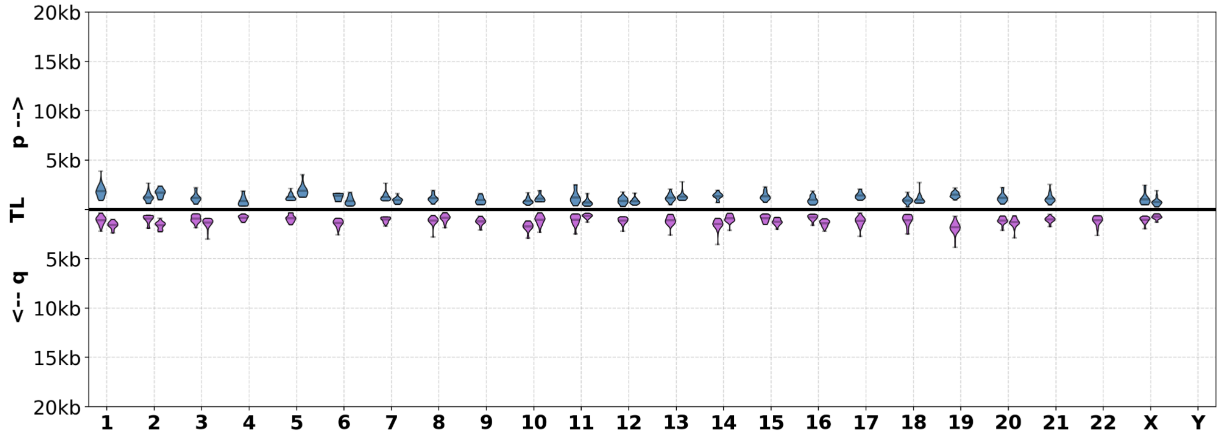

Supplementary Fig. 47: Estimated lengths of telomeres in normal pancreatic tissue and tumor cell line using Telogator with telomeres assigned based on alignments to the assemblies. (a) Telomere lengths of all individual alleles (except unresolved 21/22p) in HG008-N-P, as measured from ONT long reads. (b) Telomere lengths of 66 alleles identified in HG008-T, as measured from ONT ultralong reads. The absent alleles correspond to telomere ends that are not present in the tumor genome due to deletions or chromosome fusions.

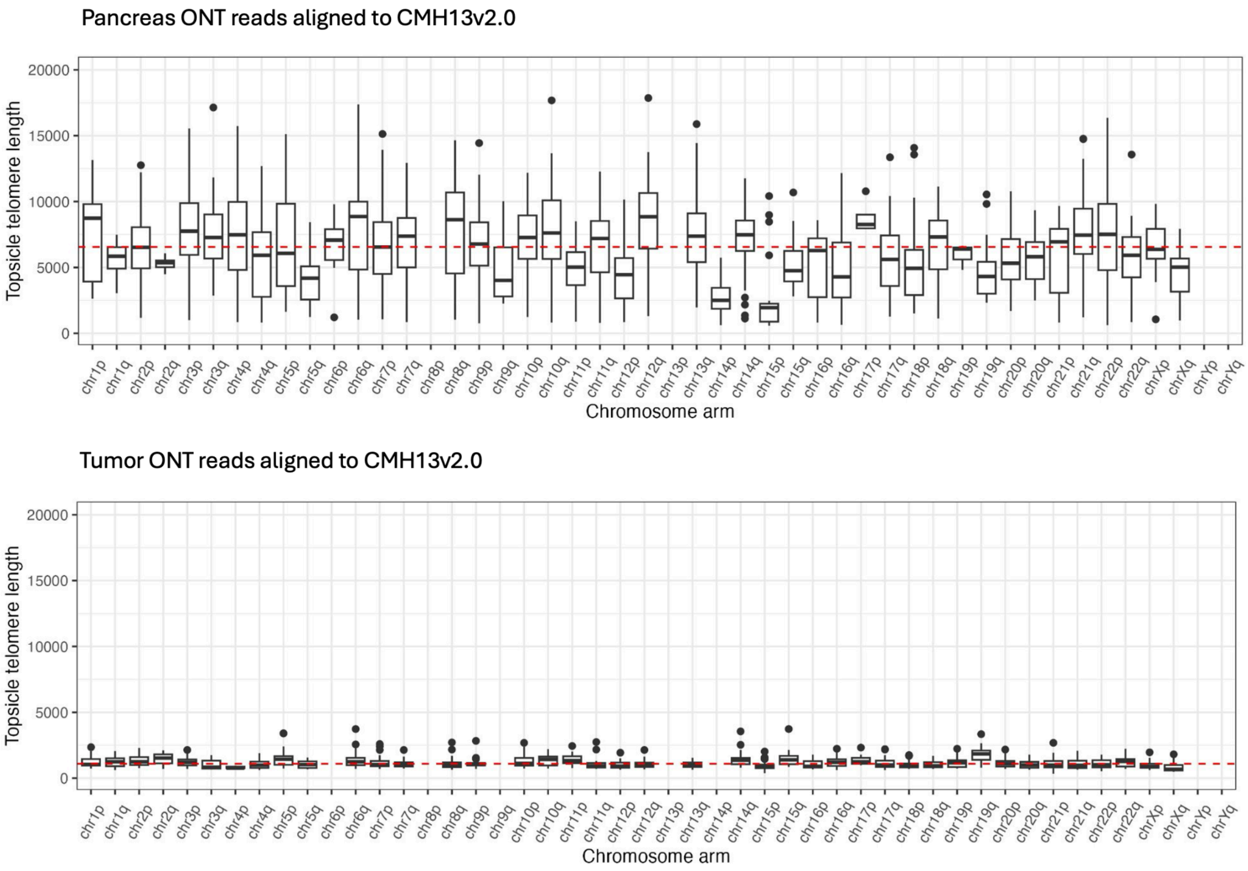

Supplementary Fig. 48: Estimated lengths of telomeres in normal pancreatic tissue and tumor cell line using Topsicle with telomeres assigned based on alignments to GRCh38. (a) Telomere lengths of all individual alleles (except unresolved 21/22p) in HG008-N-P, as measured from ONT long reads. (b) Telomere lengths of 66 alleles identified in HG008-T, as measured from ONT long reads. The absent alleles correspond to telomere ends that are not present in the tumor genome due to deletions or chromosome fusions.

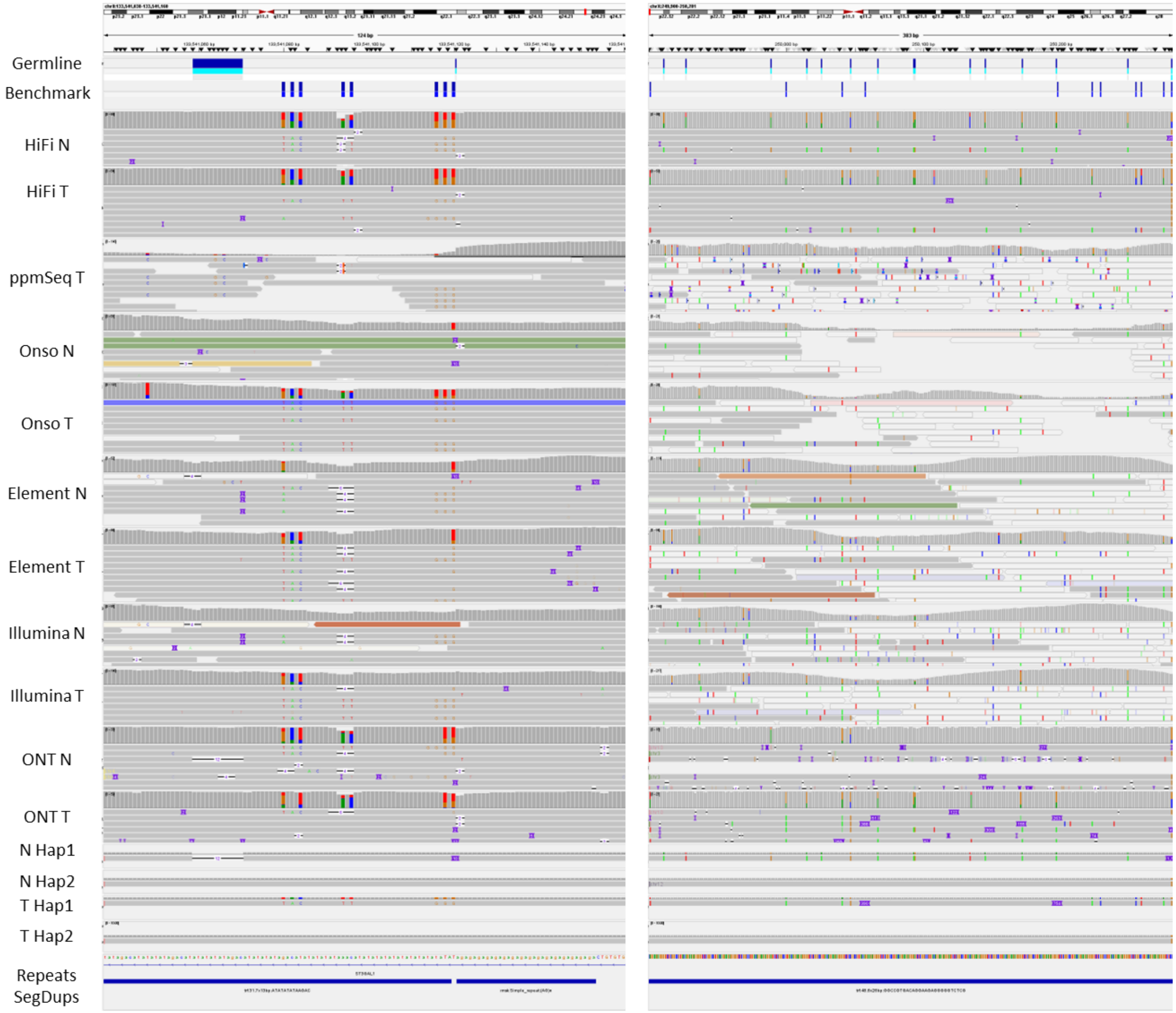

Supplementary Fig. 49: Somatic variation frequently alters germline variants. Looking at variants lifted to GRCh38 in homopolymers and tandem repeats, somatic indels can change in size or type or appear as SNVs in the tumor reads aligned to GRCh38. As an extreme example, in chr8:133541079-133541120, a 2bp somatic insertion causes a 12bp deletion and 10bp insertion in the normal to change to 8 SNVs in adjacent diTRs. Another example is chrX:249900-250281, a 26bp deletion in a VNTR causes 9 SNVs and 2 large insertion SVs. When clustering variants within 1kbp on GRCh38, 47 of 104 clusters of >= 3 somatic variants are actually caused by a single somatic variant. These 47 somatic variants cause 204 apparent somatic variants on GRCh38, indicating that variant clusters appearing as omikli (diffuse hypermutation) or kataegis on GRCh38 are often actually a simple single somatic change.

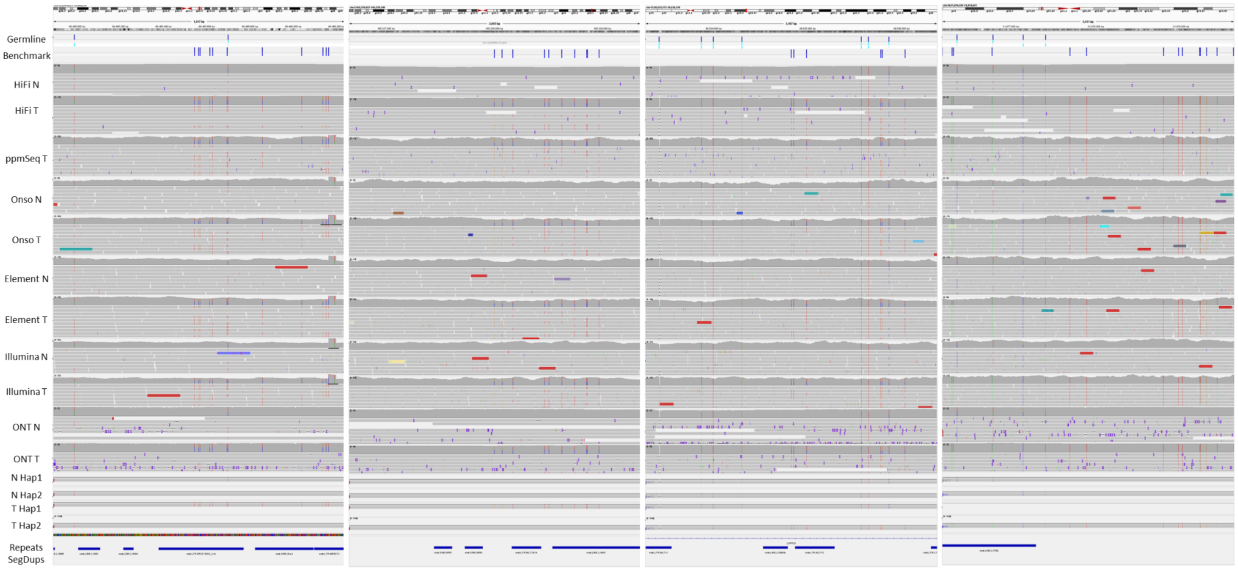

Supplementary Fig. 50: Kaetagis and Omikli events. There are some real clusters of somatic variants that appear to be typical kaetagis and omikli events (cluster size of 4+ for kataegis and 2-3 for omikli[92]). From left to right, chr3:99465564 has ~10 C>T and 3 C>G within 500bp and similar clusters in chr3:165228003, both on hap1. There are also 6 SNVs around chr13:39534827 on hap2 and 14 SNVs in chr20:21976192-21979621.

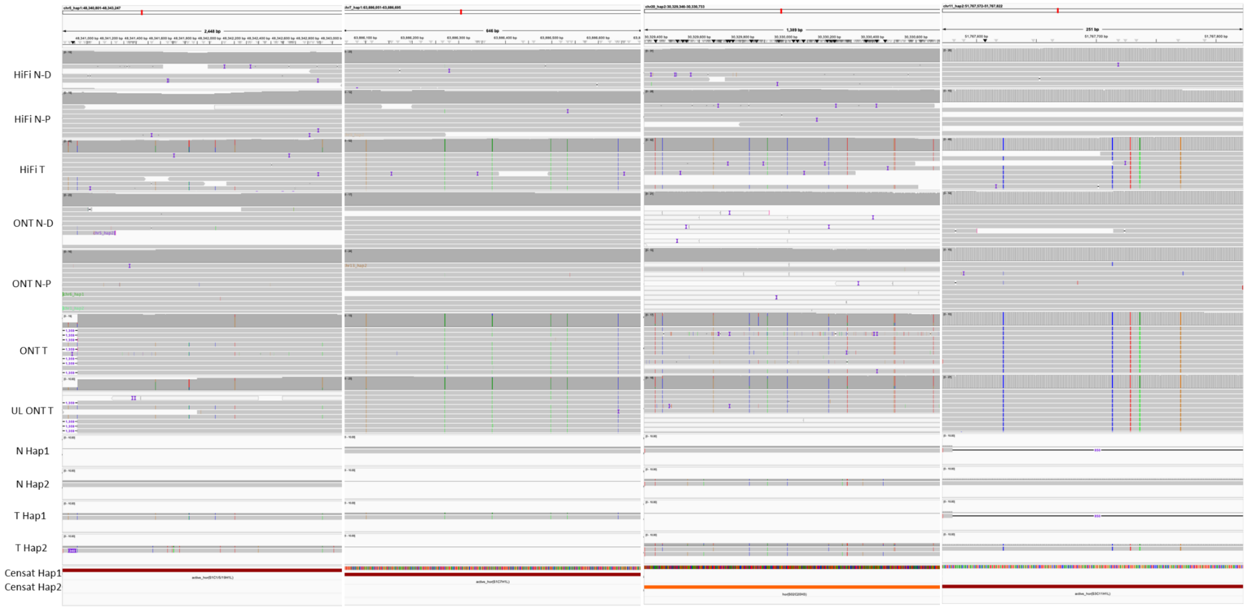

Supplementary Fig. 51: Clusters of Variants in Centromeres. Examining clusters of SNVs, several appear to be in or near centromeres which were confirmed by centromere annotation including chr5_hap1:48340801-48343247 that seems to be due to suboptimal alignment of SV in centromere. A cluster of 6 SNVs in chr7_hap1:63886051-63886695 that is in the centromere track. Another cluster of 11 SNVs in chr20_hap2:30329346-30330733 between 2kb and 11kb deletions which is not an obvious suboptimal alignment since it has evidence in mapped reads too. Finally, a cluster of 5 SNVs in chr11_hap2:51767572-51767822 that is also within a centromere track.

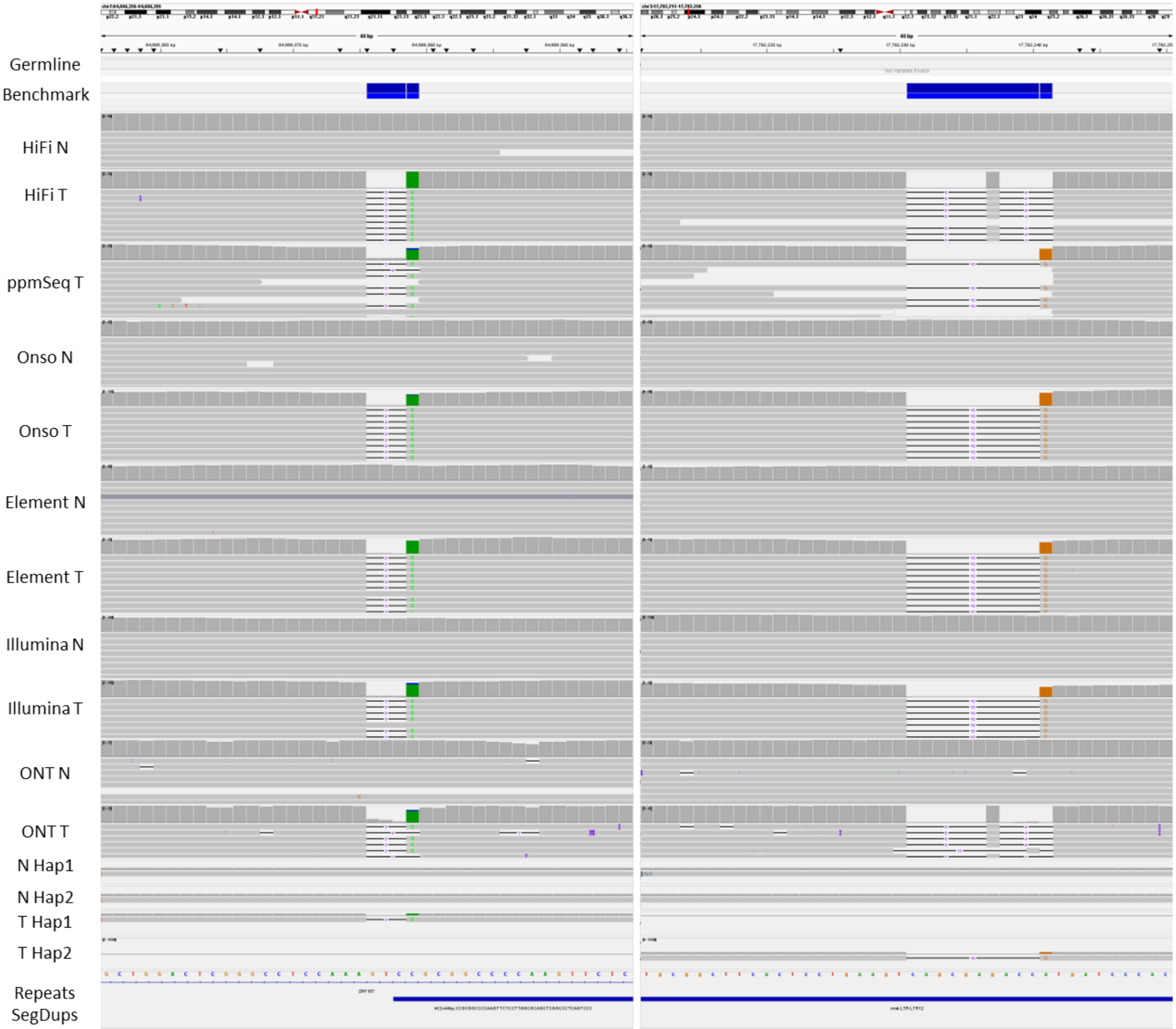

Supplementary Fig. 52: Other types of complex variants. These are two examples of complex variants though other events include MNVs. First, chr7:64666476 shows 4 bp replaced by 1 bp as seen in the alignments and assembly. Another example is at chr3:17782231 that has 11 bp replaced by 1 bp.

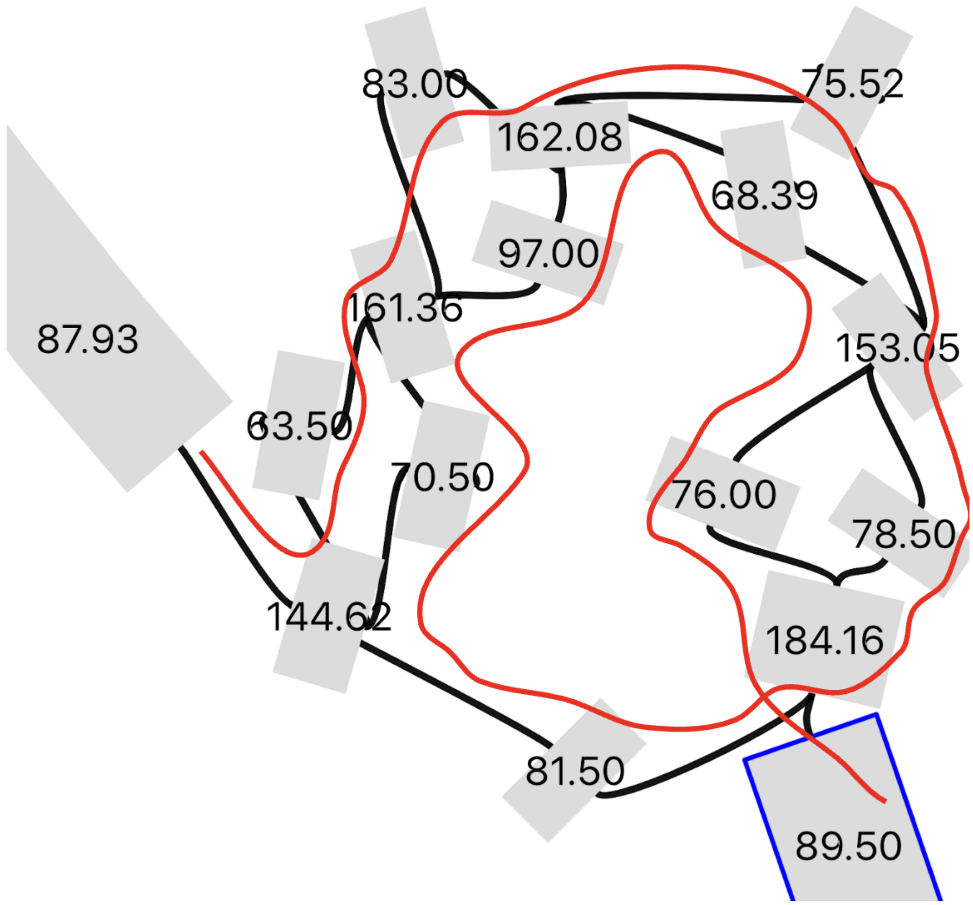

Supplementary Fig. 53: Example Assembly Graph Manual Resolution. Example of manual resolution of a 2.98 Mbp tandem duplication at chr7:4824648 (GRCh38), where numbers correspond to node coverage and red is the manually chosen path.

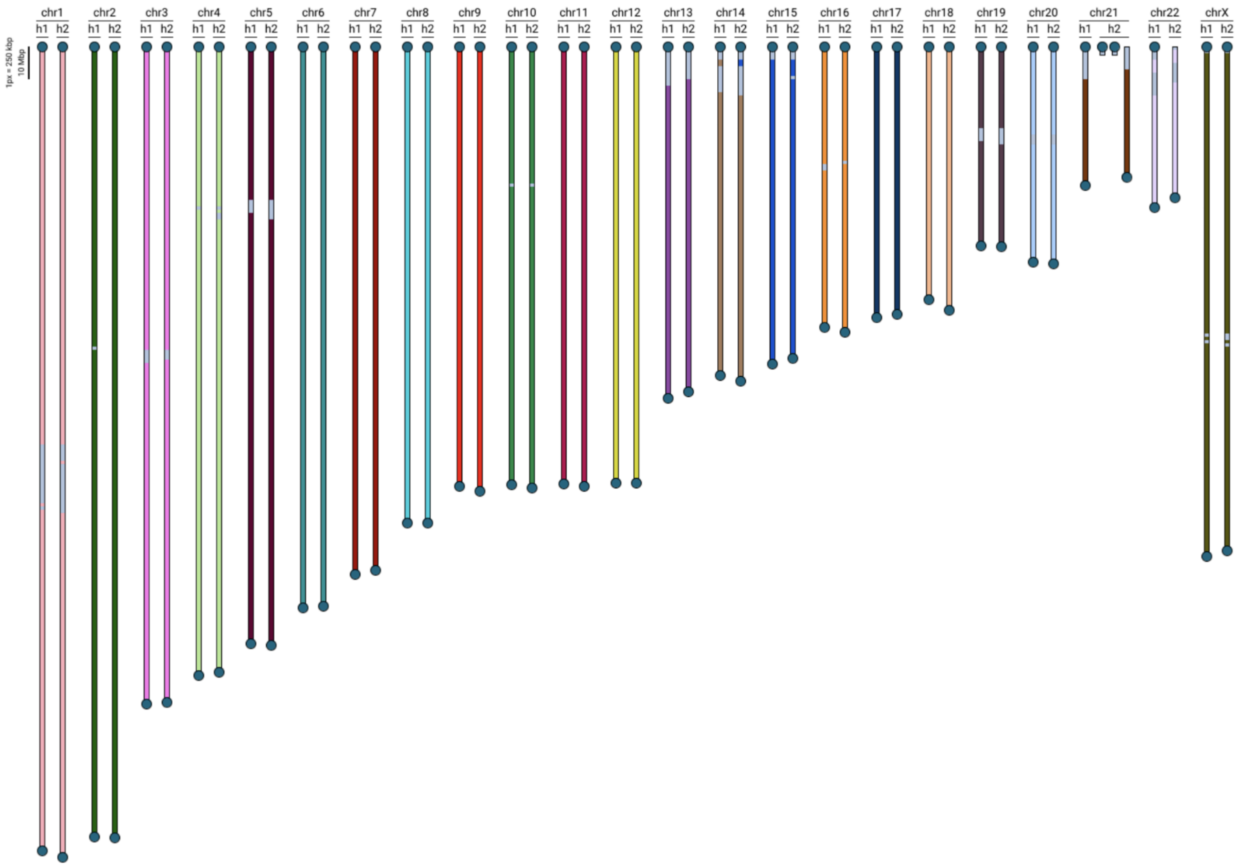

Supplementary Fig. 54: Karyoscope orientation of HG008Nv6.3 large contigs based on CHM13 kmers.
